# Toward Understanding the Role of miRNAs in Cleft Palate Only: Observations from Patient Tissues and In Vitro Assays

**DOI:** 10.3390/ijms27052088

**Published:** 2026-02-24

**Authors:** Annalisa Palmieri, Luca Scapoli, Agnese Pellati, Federico Apolloni, Valerio Zanchi, Giuseppe Spinelli, Rossella Sgarzani, Francesco Carinci, Marcella Martinelli

**Affiliations:** 1 Department of Medical and Surgical Sciences, University of Bologna, 40126 Bologna, Italy; annalisa.palmieri@unibo.it (A.P.); luca.scapoli2@unibo.it (L.S.); rossella.sgarzani2@unibo.it (R.S.); 2 Department of Translational Medicine and for Romagna, University of Ferrara, 44121 Ferrara, Italy; agnese.pellati@unife.it (A.P.); crc@unife.it (F.C.); 3 Regional Center for Diagnosis and Treatment of Cranio-Maxillofacial Malformation, Maxillofacial Unit, Ospedale San Bortolo, 36100 Vicenza, Italy; federico.apolloni@aulss8.veneto.it; 4 Department of Neurosurgery, Meyer Children’s Hospital IRCCS, 50139 Florence, Italy; valerio.zanchi14@gmail.com; 5 Department of Otorhinolaringology, ASST Valtellina e Alto Lario, 23100 Sondrio, Italy; 6 Maxillo-Facial Unit, Azienda Ospedaliero Universitaria Careggi, 50134 Florence, Italy; info@giuseppespinelli.it

**Keywords:** cleft palate only, microRNAs, palatogenesis, gene expression, craniofacial development

## Abstract

Cleft palate only (CPO) is a multifactorial craniofacial malformation with significant genetic and epigenetic contributions. Among these, microRNAs (miRNAs) have emerged as key regulators of palate development, although their alterations in CPO remain incompletely characterized. In this study, we performed a comprehensive miRNA expression analysis on palatal tissues from an Italian cohort of non-syndromic CPO patients, compared with a human embryonic palatal mesenchymal (HEPM) cell line. Using the NanoString^®^ nCounter^®^ platform for miRNA profiling, we identified significant deregulation of several miRNAs, notably the upregulation of miR-205-5p and miR-200c-3p and the downregulation of miR-125a-5p in CPO tissues. Based on these expression changes, a functional analysis was carried out to identify potential target genes. Validation in primary cell cultures derived from patient tissues confirmed these expression patterns. Functional analyses and target predictions implicated PAX9, a key transcription factor essential for palatogenesis, as a probable target of miR-205-5p, while miR-125a-5p was associated with the regulation of PRTG and PRSS35—genes involved in neural crest cell biology and extracellular matrix remodeling, respectively. Although modulation of certain predicted targets of miR-200c-3p was observed, in vitro inhibition experiments did not show significant changes in gene expression, highlighting the complexity of miRNA regulatory networks and the need for further studies to unravel these interactions. These findings identify miRNA alterations associated with CPO tissue and fibroblasts, highlighting novel candidate pathways for further mechanistic and therapeutic investigation.

## 1. Introduction

Orofacial clefts are common craniofacial anomalies with an average prevalence of approximately 1 in 1000 live births. Hundreds of syndromes may include a cleft as a feature, but among isolated cases (~70%), clinical assessment recognizes at least three distinct phenotypic groups: cleft lip only (CLO), cleft lip with or without palate (CL/P), and cleft palate only (CPO) [1]. Although there is still much to be understood, it seems clear that these heterogeneous phenotypes, with different underlying etiological mechanisms, have complex, multifactorial causes. Indeed, genetic mutations, environmental factors, and epigenetic imbalances have been shown to play a role, often in concert, in the occurrence of these common defects of orofacial development [2].

In this paper, we focus on CPO, the least frequent and least investigated form of orofacial clefts. While syndromic forms of cleft palate are often associated with specific genetic mutations and additional anomalies, most cases are non-syndromic and occur sporadically without other overt clinical features. Proper human palatal closure, occurring between weeks 6 and 12 of gestation, depends on coordinated proliferative, migratory, and fusional processes involving two distinct palatal shelves. This tightly regulated process requires the sequential activation of specific genes [3]. In recent years, genome-wide association analyses have been performed to identify genes involved in the non-syndromic form of cleft palate (nsCPO); however, so far, only a single common variant associated with nsCPO has been identified using this approach: the rs41268753 at the grainyhead-like 3 gene (GRHL3 [MIM 608317]) [4]. The identification of GRHL3 supports the importance of genetic factors in the etiology of nsCPO; however, GRHL3 polymorphism does not account for the high heritability of CPO. This evidence highlights the need for further research into epigenetic mechanisms and regulatory elements, such as miRNAs. An increasing number of studies have focused in the last decade on the role of miRNAs in the deregulation of gene expression during normal and abnormal orofacial embryogenesis [5]. These small non-coding RNAs modulate critical morphogenetic processes necessary for palatal shelf fusion, such as cell proliferation, differentiation, migration, and epithelial–mesenchymal transition [5].

Despite significant advances in understanding, the role of miRNAs in the pathogenesis of CPO remains insufficiently characterized. This study aims to identify and characterize specific miRNAs involved in nsCPO pathogenesis, hypothesizing that deregulated miRNA expression contributes to abnormal palatal development. We identified at least one miRNA potentially implicated in the pathogenesis of human cleft palate. We validated the correlation between miRNA and target gene expression in primary cultures derived from the palatal mucosa of very young CPO patients (all under one year of age) undergoing their first palate reconstruction surgery. To our knowledge, this study is the first to report transfection of fibroblast cell lines from CPO patients with microRNA mimics (to upregulate expression) or inhibitors (to downregulate expression).

## 2. Results

### 2.1. Identification of Deregulated miRNAs in CPO Patients

**Figure 1:**
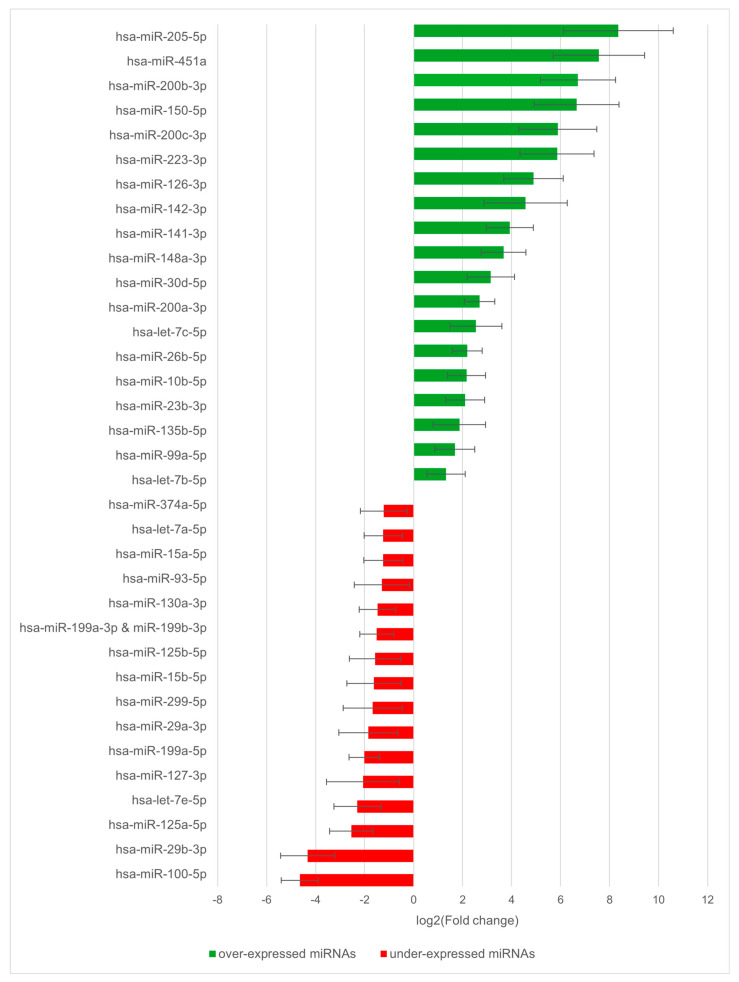
miRNAs significantly deregulated in palatal mucosa of CPO patients.

A preliminary screening experiment was performed to identify miRNAs deregulated in patients with cleft palate only (CPO) malformation. The miRNA expression profiles of fresh palatal tissues collected during the initial corrective surgery from 11 CPO patients were compared to those of HEPM cells, a human embryonic palatal mesenchymal cell line representing normal palate development. Using the NanoString^®^ nCounter^®^ miRNA Expression platform, a panel comprising 827 miRNAs was analyzed. The comprehensive list of miRNAs along with the raw expression data is provided as Supplementary Material (Table S1). Based on the nCounter^®^ Differential Expression Call, which evaluates changes exceeding the 95% threshold of technical variability, a total of 35 miRNAs were identified as differentially expressed candidates. Specifically, 19 miRNAs showed an increase in expression, while 16 were decreased in CPO samples relative to the HEPM control. These miRNAs were considered consistently deregulated within the conservative parameters of the platform’s error model and were prioritized for further biological validation (Figure 1).

Data are expressed as mean log2 fold change calculated from 11 individual CPO patient biopsies relative to the HEPM control cell line. Error bars represent the standard deviation across the 11 biological replicates, reflecting inter-individual variability. Statistical significance was determined using the nSolver™ Differential Expression Call (confidence threshold equivalent to *p* < 0.05), a specialized error model that evaluates whether observed count differences exceed platform technical noise and biological background.

### 2.2. Validation of miRNA Dysregulation and Target Gene Expression in Primary CPO Cell Cultures

Following the identification of deregulated miRNAs in the preliminary screening, primary cell cultures were established from seven patient specimens to further investigate the regulatory mechanisms involved in CPO development. Real-time PCR was performed to analyze the expression of the most dysregulated miRNAs and some of their predicted target genes. Specifically, the expression levels of the five most upregulated and three most downregulated miRNAs identified in Figure 1 were evaluated. miRNA expression in patient-derived cell cultures was compared to that of HEPM cells, with the results summarized in Table 1.

Consistent with the preliminary screening data from fresh tissue, expression levels of miR-205-5p and miR-200c-3p were significantly elevated in CPO cells, while miR-125a-5p expression was significantly reduced. Conversely, miR-100-5p and miR-29b-3p did not show significant differences compared to the control. Three of the original eight targets (miR-150-5p, miR-223-3p, and miR-142-3p) were excluded from final analysis due to failed qPCR amplification across all samples (seven CPO patient-derived cultures and HEPM control). This uniform lack of signal, irrespective of cell type, indicates technical issues with the primer sets rather than biological variability or sample degradation.

Potential target genes of miR-205-5p, miR-200c-3p, and miR-125a-5p were selected based on prediction scores from miRDB (mirdb.org/) and TargetScan 7.2 databases [6], and their expression levels were analyzed in cultured cells. Since miRNAs generally act as negative regulators of gene expression, the predicted target genes of miR-205-5p and miR-200c-3p were expected to be downregulated, whereas the predicted target genes of miR-125a-5p were expected to be upregulated in CPO cells. Most of the genes investigated showed expression changes consistent with these expectations, although not all changes reached statistical significance or were fully concordant, as detailed in Table 2.

The inhibition of overexpressed miR-205-5p and miR-200c-3p in CPO patient-derived cells did not result in increased expression of their target genes (Table 3).

Indeed, the expression levels of all investigated genes remained largely unchanged following miRNA inhibition. In contrast, the positive control assay (transfection with a let-7c-5p inhibitor) significantly upregulated the HMGA2 reporter gene (fold change 8.98, *p* = 0.010 for miR-205-5p experiments and fold change 5.74, *p* < 0.001 for miR-200c-3p experiments).

Cell transfection with oligonucleotides mimicking the under-expressed miR-125a-5p, as expected, resulted in decreased expression of its potential target genes (Table 4), with PRSS35 and PRTG showing significant inhibition. The miR-1 positive control confirmed the inhibition protocol’s effectiveness, downregulating the target gene PTK9 (fold change 0.163, *p* < 0.001).

## 3. Discussion

A growing body of evidence implicates miRNA–mRNA regulatory networks in both normal craniofacial development and the pathogenesis of craniofacial anomalies, including cleft palate [7,8]. Epidemiological studies have reported considerable variability in the prevalence of CPO across different ethnic groups and geographical regions, reflecting the interplay between genetic heterogeneity and environmental factors [9]. This heterogeneity underscores the importance of geographically focused studies to identify population-specific molecular mechanisms involved in CPO development.

In this context, the present study aimed to identify deregulated miRNAs in palatal tissue from Italian CPO patients by comparison with a commercially available human embryonic palatal mesenchymal (HEPM) cell line, which is widely used as an in vitro model of palatal mesenchyme. Using NanoString^®^ Technologies, miRNA expression profiles were assessed from palatal tissue samples of 11 patients and compared with HEPM cells. We identified 19 significantly upregulated and 16 downregulated miRNAs. Expression profiling in patient-derived primary cell cultures confirmed the differential expression of key miRNAs, providing consistency between tissue and cellular models. Based on fold change and statistical significance, three miRNAs (two upregulated and one downregulated) were selected for functional analysis. Target genes of the upregulated miRNAs miR-205-5p and miR-200c-3p, as well as the downregulated miRNA miR-125a-5p, were selected based on their known involvement in CPO development [10] and their predicted regulatory relationships with these miRNAs, with the aim of pinpointing the underlying molecular mechanisms. Expression analyses of these target genes in primary cells revealed patterns generally consistent with expected miRNA regulatory effects, although not all changes were statistically significant or directionally concordant (Table 2). Finally, to evaluate miRNA-target gene interactions, miR-205-5p and miR-200c-3p downregulation and miR-125a-5p overexpression were induced in primary cell lines from CPO patients using miRNA inhibitors and mimics, respectively.

Notably, miR-205-5p has not been previously implicated in palatogenesis or the etiology of cleft palate. However, a study by Wang et al. [11] reported miR-205-5p as the most deregulated miRNA in non-syndromic cleft lip with or without cleft palate (nsCL/P) tissues. It is important to highlight that their findings were based on a limited number of samples and mixed tissue types (two lip mucosa and two palatal mucosa), which warrants caution when extrapolating these results specifically to isolated cleft palate cases. This underscores the need for further investigation of miR-205-5p in the specific context of CPO.

In our cohort of 7 primary CPO cell lines, PAX9, a predicted high-confidence target of miR-205-5p, was significantly downregulated. It is a transcription factor member of the paired box family, which has been shown to play a critical role in human palate development [12] and has also been implicated in the etiology of cleft palate in mouse models [13,14,15]. Despite significant PAX9 downregulation in CPO samples, miR-205-5p inhibition failed to rescue its expression, suggesting that this miRNA may not be the primary repressor in this cellular context. Although miR-205-5p and PAX9 show inverse correlation, their functional interaction may be stage-specific (e.g., limited to early embryogenesis) or overshadowed by dominant regulatory pathways in patient-derived fibroblasts.

The upregulation of miR-200c-3p observed in cultures of cells derived from our CPO patients corroborates the findings of Won et al. [16], who demonstrated, using a mouse palate culture model, that miR-200c plays a crucial role in palate development by regulating E-cadherin expression, cell death, and palate shelf motility. Consistently, we observed significant downregulation of HIPK3, NR5A2, and VASH2, each with a top prediction score of 100 (miRDB; https://mirdb.org/), and TBK1, with a prediction score of 93, in our primary CPO cell lines as miR-200c-3p targets. For HIPK3 and VASH2, downregulation showed nominal significance in primary cell line expression analysis but lost significance after multiple testing corrections; TBK1 remained significantly downregulated in primary lines but lost significance after miR-200c-3p inhibition. The NR5A2 gene encodes the transcription factor nuclear receptor subfamily 5, group A member 2 (NR5A2). Its function is vital to several physiological processes, including normal physiology, homeostasis, embryonic development, lipid metabolism and anti-inflammatory activities [17]. VASH2 belongs to the VASH family together with VASH1; it activates actin-binding, metallocarboxypeptidase and microtubule-binding activities and has been shown to promote angiogenesis in the process of injury repair [18]. TBK1, a kinase implicated in innate immunity and development, has also been associated with increased CPO risk in maternal smoking cases in a multiple single-nucleotide polymorphism association study [19]. HIPK3, encoding a homeodomain-interacting protein kinase involved in diverse cellular functions, has a less clear role in craniofacial development and merits further study. However, the inhibition of miR-200c-3p in our cell model did not lead to a significant upregulation of these targets. Thus, they represent candidate genes whose downregulation in CPO may be independent of miR-200c-3p or modulated by additional epigenetic/transcriptional controls unaffected by transient inhibition.

Among downregulated miRNAs, miR-125a-5p showed the most prominent under-expression. Several predicted targets, including PRTG, MAP6, and DVL3, were upregulated. Transfection of primary CPO cell lines with a miR-125a-5p mimic confirmed that PRTG is a target gene of this miRNA. Interestingly, protogenin (PRTG), as well as DVL3, is present in the neural crest differentiation pathway (PathCards.genecards.org), as it enhances the migration and survival of cephalic neural crest cells, which are responsible for facial and oral development [20]. Previous studies have linked low-frequency coding variants in PRTG to nsCL/P [21]. More recently, Siewert et al. investigated the expression pattern of PRTG in specific cell populations from the pharyngeal arches, which are responsible for mesenchyme development and cell migration, to explore its potential role in nsCL/P etiology [22]. Based on these insights, we hypothesize that PRTG upregulation may impair the survival and function of cranial neural crest cells, which are essential for palatogenesis. Such dysregulation could lead to failure of palatal shelf fusion during embryogenesis, resulting in cleft palate. Importantly, our study is the first to implicate PRTG in a well-defined, homogeneous cohort of CPO patients, thereby extending the gene’s suspected involvement from nsCL/P to isolated cleft palate. These findings provide valuable insights into PRTG alterations associated with CPO patient-derived fibroblasts. Similarly, we identified PRSS35 as a predicted target of miR-125a-5p (confirmed by mimic transfection), representing the first report linking PRSS35 to CPO. PRSS35 encodes a serine protease that belongs to a group of proteins with critical roles in fundamental biological processes. In a case–control study, single-nucleotide polymorphisms mapping to the PRSS35 gene were originally found to be associated with CL/P in several subgroups of the main cohort [23]. More recently, the association has been confirmed by de Araujo’s group, although still limited to a study group of CL/P patients [24]. This finding aligns with previous evidence demonstrating Prss35 expression in craniofacial and palatal tissues during critical windows of murine embryogenesis, particularly at embryonic days 12 and 13, corresponding to the stages of palatal shelf growth and fusion [23]. Given the role of proteases in extracellular matrix remodeling and cell migration, dysregulation of PRSS35 may impair palatogenesis by disrupting these processes. Therefore, we hypothesize that disturbed expression of PRSS35 may contribute to CPO pathogenesis by interfering with the molecular mechanisms governing palatal shelf elevation and fusion. This hypothesis underscores the need for mechanistic studies to clarify the functional consequences of PRSS35 perturbation during human palate development and its contribution to CPO. MAP6, a microtubule-associated protein involved in stabilizing cytoskeletal architecture, emerged as another miR-125a-5p target of interest, though its precise role in CPO remains to be elucidated.

Overall, the strong concordance observed between miRNA expression profiles in fresh tissues and corresponding primary fibroblast cultures supports the involvement of miR-205-5p, miR-200c-3p, and miR-125a-5p in CPO pathogenesis, suggesting that primary fibroblasts serve as a relevant model for studying miRNA-mediated mechanisms in CPO development.

However, study limitations include the use of a single, commercially available control cell line (HEPM) rather than patient-matched healthy tissue, primarily due to ethical and practical constraints. Moreover, primary cultures derived from palatal tissues predominantly comprised fibroblasts; while these cells are key mesenchymal components in palatogenesis, the absence of other cell types may influence miRNA expression profiles and regulatory interactions. Furthermore, it is important to note that our findings are based on expression profiling and miRNA mimic/inhibitor assays. In the absence of direct binding evidence, such as 3′UTR luciferase reporter assays (not feasible in our current laboratory setup), we cannot exclude the possibility that the observed expression changes result from indirect regulatory effects rather than direct miRNA–mRNA interactions. Future studies employing biochemical validation of these targets (e.g., PAX9, PRTG, and PRSS35) are necessary to confirm the mechanisms of these networks. Despite these constraints, our study offers novel insights by combining unbiased miRNA discovery in a homogeneous cohort of very young Italian CPO patients (mean age 8 months ± 4) with functional validation in patient-derived cells, thereby bridging in vivo observations with mechanistic interrogation.

## 4. Materials and Methods

### 4.1. Specimen Collection

For the present study, mucosal tissue samples (a mixture of an indeterminate amount of lining epithelium and lamina propria) were collected from surgical excisions that were not used for palate reconstruction in patients affected by CPO. In the operating theater, a portion of each fragment was immersed in PBS for rapid culturing to isolate fibroblasts, while another portion was preserved in RNAlater (Sigma-Aldrich, Inc., St. Louis, MO, USA) for subsequent RNA extraction. Informed consent was obtained from the parents of patients who were included in the study, and the protocol was approved by the relevant ethics committees for the Meyer Hospital in Florence and the San Bortolo Hospital in Vicenza, both in Italy. A thorough medical history was conducted on all patients, ruling out the presence of features attributable to specific syndromes, neurological disorders, or metabolic diseases. Additionally, the presence of recurrent diseases within the family and the potential impact of known teratogenic substances were excluded. The clinical data of the patients are summarized in Table 5.

### 4.2. Isolation and Primary Culture of Human Palatal Fibroblasts

Primary human fibroblast cells were isolated from ex vivo palatal mucosa fragments obtained from surgical resections. The specimen was cut into small pieces and placed in a 24-well plate. Each fragment was covered with a drop of DMEM/F12 medium (Sigma-Aldrich) supplemented with 50% fetal bovine serum (FBS), 2% penicillin/streptomycin, and 1% L-glutamine (all from Sigma-Aldrich). The plates were incubated at 37 °C in a humidified atmosphere of 5% CO_2_ to facilitate cell adhesion. Although the starting material contained both epithelial and mesenchymal components, fibroblasts rapidly predominated in culture as epithelial cells failed to adhere and proliferate. Subsequently, additional medium was added, and fibroblast outgrowth was typically observed after approximately 10 days. The medium was then replaced with DMEM/F12 supplemented with 10% FBS and antibiotics, and cells were cultured until they reached confluence. Once confluent, fibroblasts were transferred to T25 flasks and expanded to obtain a sufficient quantity for experimental analyses. Seven of the 11 primary cultures were selected for downstream experiments due to superior tissue quality, efficient cell adhesion, robust outgrowth, and consistent cellular morphology. Notably, the seven selected cell lines were derived from patients who had undergone surgery at an age no greater than 10 months.

### 4.3. RNA Isolation

Ex vivo surgical tissue fragments were homogenized utilizing Potter–Elvehjem tissue grinders (Bellco Glass Inc., Vineland, NJ, USA). Cultured cells were harvested using trypsin/EDTA and subsequently pelleted by centrifugation at 500× *g* for 1 min. In both cases, total RNA was extracted and purified employing the Quick-RNA Microprep Kit (Zymo Research, Irvine, CA, USA). Briefly, samples were lysed with RNA Lysis Buffer and purified using a silica filter column, following the manufacturer’s instructions. The quantity of RNA and absence of contaminants were assessed using a Nanodrop 2000 spectrophotometer (Thermo Scientific, Waltham, MA, USA).

### 4.4. miRNA Expression Profiling

RNAs isolated from the palatal mucosa of 11 patients and from a commercial fibroblast cell line derived from embryonic palatal shelves (HEPM; LGC Standards, Milan, Italy; Lot/Batch No: 58798172) were processed for miRNA expression profiling using NanoString^®^ Technology nCounter^®^ Human v3 miRNA Expression Assay (NanoString, Seattle, WA, USA) at the manufacturer’s facility (Diatech Lab Line, Jesi, Italy). The HEPM cell line was utilized as a standardized biological baseline. To maximize the detection of clinical variance, we prioritized the analysis of 11 independent biological replicates from patient biopsies over technical replicates of the cell line, as the latter would only reflect platform-related technical noise rather than biological heterogeneity. The NanoString^®^ nCounter^®^ raw data were analyzed with the nSolver™ software 4.0. Geometric mean of negative control probe counts was used to perform background subtraction. Raw expression data was normalized in a two-step process; the first normalization factor was calculated using geometric mean of the positive controls that were spiked into every sample, while geometric mean of all genes was used for CodeSet content normalization. Expression levels of miRNA in CPO biopsies were compared to HEPM cell line through fold change ratio calculation and the differential expression call (DE Call), a test developed for nCounter^®^ exploratory studies data. The nSolver™ DE Call error model evaluates whether the ratio between groups exceeds the platform’s technical noise. This model is based on an empirical mapping of raw counts to an estimated 95% level of technical variability (calculated on a log scale). miRNAs were considered significant only when flagged as ‘YES’ by the DE Call, indicating that the observed deregulation is demonstrably different from the technical background. To further ensure biological stringency, results were filtered by fold change (>2) and validated via RT-qPCR.

### 4.5. Reverse Transcription

All miRNAs were reverse transcribed from 1000 ng of total RNA in a final reaction volume of 10 µL. This reaction contained 1 µL of 10X poly(A) polymerase buffer, 1 mM ATP (both from New England Biolabs, Ipswich, MA, USA), 0.1 mM of each deoxynucleotide (dATP, dCTP, dGTP, and dTTP) (Sigma-Aldrich), 100 units of MuLV reverse transcriptase (New England Biolabs), and 1 µM reverse transcription (RT) primer (biomers.net GmbH, Ulm, Germany). The sequence of the RT primer was 5′-CAGGTCCAGTTTTTTTTTTTTTTTVN-3′, where V represents A, C, or G and N represents A, C, G, or T [25]. The reaction was incubated at 42 °C for 1 h, followed by a 5 min inactivation at 95 °C. For target gene expression analysis, cDNA synthesis was performed using 500 ng of total RNA and the PrimeScript RT Master Mix kit (Takara Bio, Kusatsu, Japan), following the manufacturer’s protocol. The thermal profile for this reaction included incubation at 37 °C for 15 min, followed by inactivation at 85 °C for 5 s.

### 4.6. Real-Time PCR Amplifications

Quantitative PCR assays were designed to investigate both miRNAs and their potential target genes. Primer sequences for miRNA expression analysis were designed using the miRprimer software [26], while primer sequences for gene expression analysis were designed with the aid of primerBlast tool on the NCBI website [27]. All miRNA and target gene primer sequences are provided in Table S2 (Supplementary Materials). Quantitative real-time PCR (qPCR) was performed in a final volume of 20 μL, consisting of 10 μL Power SYBR Green Master Mix (Life Technologies, Foster City, CA, USA), 100 nM each of forward and reverse primers, and 300 nM cDNA. Reactions were conducted on a QuantStudio™ 5 Real-Time PCR System (Thermo Fisher Scientific). The cycling conditions comprised an initial denaturation at 95 °C for 2 min, followed by 40 cycles of 15 s at 95 °C and 60 s at 60 °C. A melt curve analysis was performed at the end of each assay to verify amplification specificity. All reactions were carried out in analytical duplicates. Negative controls were included in each experiment to rule out biological contamination. For normalization, expression levels of target genes were referenced to RPL13, while miRNA quantification utilized RNU44 as the endogenous control.

The relative gene expression was quantified with the ΔΔCt calculation method [28], using RNU44 as reference gene for miRNAs and RPL13 as reference gene for target genes. In order to compare the ΔCt values between CPO and HEPM cells, normality was assessed using the Shapiro–Wilk test; subsequently, Welch’s *t*-test was employed to account for unequal variances, as confirmed by Levene’s test. A paired sample *t*-test was used to compare the ΔCt values between transfected CPO cells and their vehicle-treated controls. Expression level differences were calculated as fold changes using the formula 2^−ΔΔCt^. Due to the exploratory nature of this study and the multiple molecular targets analyzed, we addressed the risk of false discoveries by applying the Benjamini–Hochberg False Discovery Rate (FDR) procedure to control for Type I errors. This correction was applied independently within each experiment. Results were considered statistically significant when the FDR-adjusted *p* value (*q* value) was <0.05.

### 4.7. Transfection with miRNA Inhibitors and Mimics

Primary human fibroblasts were seeded into 6-well plates to achieve approximately 60% confluence at the time of transfection. Oligonucleotides for mirVana™ miRNA mimic (hsa-miR-125a-5p) and miRNA inhibitors (hsa-miR-205-5p and hsa-miR-200c-3p) (Thermo Fisher Scientific) were reconstituted in sterile, nuclease-free water to obtain stock solutions at 100 μM. Working solutions at 10 μM were freshly prepared by diluting the stock solutions in sterile nuclease-free water immediately prior to use. Lipofectamine™ RNAiMAX (Thermo Fisher Scientific) was diluted in Opti-MEM™ reduced-serum medium (Thermo Fisher Scientific) in accordance with the manufacturer’s protocol. For each well, 30 pmol of miRNA mimic or inhibitor was diluted in 125 μL of Opti-MEM™. The diluted miRNA solution was mixed 1:1 with the diluted Lipofectamine™ RNAiMAX and incubated at room temperature for 5 min to allow complex formation. The culture medium was gently removed to eliminate non-adherent and dead cells and replaced with fresh DMEM/F12 serum-free medium. The miRNA–Lipofectamine™ complex was then added to each well, and cells were incubated at 37 °C for 48 h. To assess transfection efficiency and specificity, both positive and negative controls were included. The mirVana™ miRNA inhibitor let-7c positive control (Thermo Fisher Scientific) was used as an inhibitory control, with successful inhibition validated by upregulation of its target gene HMGA2, as measured by RT-qPCR. For mimic transfection, the mirVana™ miRNA mimic miR-1 positive control (Thermo Fisher Scientific) was employed, assessing downregulation of its target gene PTK9. A negative transfection control, mirVana™ miRNA mimic/inhibitor Negative Control #1 (Thermo Fisher Scientific), was included to establish baseline expression levels of the tested target genes. This negative control was transfected using the same methodology as the experimental mirVana™ miRNA mimic or inhibitors, as well as the positive controls.

## 5. Conclusions

Our results reveal miRNA–mRNA interactions associated with CPO in patient palatal tissue and primary fibroblast cultures. Interestingly, our findings on miR-205-5p deregulation in CPO are particularly striking given its previously unreported role in palatogenesis. This highlights a potential new avenue for understanding this developmental process. Specifically, PAX9 emerged as a key regulatory target of miR-205-5p, while miR-125a-5p appears to modulate genes such as PRTG and PRSS35, which are involved in neural crest cell function and extracellular matrix remodeling. These findings deepen our understanding of the genetic and epigenetic mechanisms underlying cleft palate and provide a solid foundation for future investigations aimed at unraveling the complex molecular pathways governing craniofacial development.

## Figures and Tables

**Table 1:** Expression levels in cultured cells from 7 CPO patients of the microRNAs identified as most deregulated by the NanoString^®^ assay.

miRNA	N	ΔΔCt	Welch’s *t*-Test	df	*p* Value	FDR *q* Value	Fold Change	95% CI	
mir205-5p	14	−5.5	−6.04	9.35	0.0001 *	0.001 *	45.39	10.96	187.96
mir200c-3p	14	−2.46	−3.44	12	0.005 *	0.013 *	5.51	1.87	16.22
mir125a-5p	14	7.47	2.95	6.02	0.025 *	0.042 *	0.01	7.70 × 10^−5^	0.41
mir29b-3p	14	−0.76	−1.1	10.93	0.293	0.366	1.69	0.59	4.84
mir100-5p	14	−0.47	−0.91	8.13	0.386	0.386	1.38	0.61	3.13

* *p* or *q* value < 0.05; df: degrees of freedom; FDR: False Discovery Rate.

**Table 2:** Expression analysis of putative target genes of selected miRNAs. Gene expression levels in cultured cells from CPO patients were compared to those in the commercial HEPM cell line.

Gene	Potential Regulator	Score (miRDB)	N	ΔΔCt	Welch’s *t*-Test	df	*p* Value	FDR *q* Value	Fold Change	95% CI	
AXIN2	miR-205-5p	89	14	0.96	1.64	11.8	0.126	0.183	0.51	0.21	1.24
CCNJ	miR-205-5p	97	14	−0.07	−0.11	8.43	0.911	0.911	1.05	0.41	2.66
CDK19	miR-205-5p	99	14	1.19	2.05	6.11	0.085	0.151	0.44	0.17	1.17
JADE1	miR-205-5p	89	14	0.23	0.49	8.51	0.633	0.723	0.86	0.42	1.76
PAX9	miR-205-5p	91	14	3.65	3.03	11.65	0.011 *	0.035 *	0.08	0.01	0.50
HIPK3	miR-200c-3p	100	14	0.36	2.28	11.53	0.042 *	0.084	0.78	0.62	0.99
NR5A2	miR-200c-3p	100	14	2.58	3.26	6.35	0.016 *	0.043 *	0.17	0.04	0.63
RECK	miR-200c-3p	99	14	0.81	1.79	7.62	0.112	0.179	0.57	0.27	1.18
TBK1	miR-200c-3p	93	14	2.68	4.28	7.72	0.003 *	0.024 *	0.16	0.06	0.43
VASH2	miR-200c-3p	100	14	1.29	2.45	10.81	0.033 *	0.075	0.41	0.18	0.91
ZEB1	miR-200c-3p	100	14	0.12	0.61	9.46	0.554	0.682	0.92	0.67	1.26
DVL3	miR-125a-5p	64	14	−2.97	−7.82	9.15	<0.001 *	<0.001 *	7.82	4.32	14.16
MAP6	miR-125a-5p	87	14	−1.36	−3.27	8.86	0.010 *	0.040 *	2.56	1.33	4.91
PRSS35	miR-125a-5p	86	14	0.33	0.41	8.16	0.690	0.736	0.79	0.22	2.87
PRTG	miR-125a-5p	95	14	−1.51	−3.29	10.3	0.008 *	0.043 *	2.85	1.41	5.79
TNFSF4	miR-125a-5p	90	14	−1.11	−1.14	8.27	0.288	0.384	2.16	0.46	10.19

* *p* or *q* value < 0.05; df: degrees of freedom; FDR: False Discovery Rate.

**Table 3:** Gene expression analysis of potential target genes after inhibition of specific miRNAs.

Inhibited miRNA	Gene	N (Pairs)	ΔΔCt	Paired t	df	*p* Value	FDR *q* Value	Fold Change	95% CI	
**miR-205-5p**	AXIN2	2	−0.20	−1.11	1	0.467	0.701	1.15	0.23	5.88
	CCNJ	2	0.19	0.36	1	0.779	0.876	0.87	0.01	96.44
	CDK19	2	−0.001	−0.004	1	0.997	0.997	1.00	0.29	3.42
	JADE1	2	−0.42	−0.75	1	0.592	0.761	1.34	0.01	182.88
	PAX9	2	0.40	1.91	1	0.307	0.553	0.76	0.12	4.77
**miR-200c-3p**	HIPK3	7	0.44	1.76	6	0.129	0.581	0.74	0.48	1.13
	NR5A2	7	0.50	1.36	6	0.222	0.666	0.71	0.38	1.32
	TBK1	7	0.76	2.84	6	0.030 *	0.270	0.59	0.38	0.93
	VASH2	7	−0.21	−1.13	6	0.301	0.677	1.16	0.85	1.58

* *p* or *q* value < 0.05; df: degrees of freedom; FDR: False Discovery Rate.

**Table 4:** Gene expression analysis of potential target genes after miR-125a-5p mimic transfection.

Gene	N (Pairs)	ΔΔCt	Paired t	df	*p* Value	FDR *q* Value	Fold Change	95% CI	
DLV3	5	0.34	1.04	4	0.356	0.445	0.79	0.42	1.48
MAP6	7	−0.09	−0.42	6	0.689	0.689	1.06	0.74	1.53
PRSS35	7	0.56	2.61	6	0.040 *	0.1	0.68	0.47	0.98
PRTG	7	0.59	4.88	6	0.003 *	0.015 *	0.67	0.54	0.82
TNFSF4	7	0.69	2.12	6	0.078	0.13	0.62	0.35	1.08

* *p* or *q* value < 0.05; df: degrees of freedom; FDR: False Discovery Rate.

**Table 5:** Clinical and demographic characteristics of patients included in the study.

Patient	Gender	Age (Months)	Case Classification	NanoString^®^	Validation
**1**	Female	12	Sporadic	X	
**2**	Male	12	Sporadic	X	
**3**	Male	11	Sporadic	X	
**4**	Female	11	Sporadic	X	
**5**	Female	7	Sporadic	X	X
**6**	Male	10	Sporadic	X	X
**7**	Male	5	Family history of CPO	X	X
**8**	Male	4	Sporadic	X	X
**9**	Male	6	Sporadic	X	X
**10**	Male	4	Sporadic	X	X
**11**	Male	6	Sporadic	X	X

## Data Availability

The raw data are viewable in the table uploaded as Supplementary Material (Table S1). All miRNA and target gene primer sequences are provided in Table S2.

## Supplementary Materials

The following supporting information can be downloaded at https://www.mdpi.com/article/10.3390/ijms27052088/s1.

## Abbreviations

The following abbreviations are used in this manuscript:

CL/P	Cleft Lip with or without Cleft Palate
CLO	Cleft Lip Only
CPO	Cleft Palate Only
DE	Differential Expression
EMT	Epithelial-to-Mesenchymal Transition
FBS	Fetal Bovine Serum
HEPM	Human Embryonic Palatal Mesenchymal (cell line)
miRNA	microRNA
nsCL/P	Non-syndromic Cleft Lip with or without Cleft Palate
nsCPO	Non-syndromic form of Cleft Palate Only
RT-qPCR	Reverse Transcription–quantitative Polymerase Chain Reaction
SNP	Single-Nucleotide Polymorphism
