# Supplementary material for: Toward Understanding the Role of miRNAs in Cleft Palate Only: Observations from Patient Tissues and In Vitro Assays
Source: Int J Mol Sci. 2026 Feb 24;27(5):2088. doi: 10.3390/ijms27052088 (PMC12985306; doi:10.3390/ijms27052088)
Supplement: Supplementary file 1 [file ijms-27-02088-s001.zip › Table S1.pdf]

**Table S1.** Raw data from NanoString® nCounter® miRNA Expression platform.

| Probe name | Accession # | Analyte type | % Samples above threshold | HEPM | Patient 1 | Patient 2 | Patient 3 | Patient 4 | Patient 5 | Patient 6 | Patient 7 | Patient 8 | Patient 9 | Patient 10 | Patient 11 |
|---|---|---|---|---|---|---|---|---|---|---|---|---|---|---|---|
| hsa-let-7a-5p | MIMAT0000062 | miRNA | 100 | 9515 | 12911 | 6598 | 8643 | 4811 | 8523 | 2378 | 1558 | 7751 | 3705 | 4370 | 11386 |
| hsa-let-7b-5p | MIMAT0000063 | miRNA | 100 | 888 | 6792 | 3023 | 7369 | 2517 | 5254 | 1674 | 1227 | 4307 | 1521 | 1764 | 4693 |
| hsa-let-7c-5p | MIMAT0000064 | miRNA | 100 | 128 | 2171 | 798 | 1485 | 679 | 2149 | 342 | 309 | 2499 | 671 | 893 | 2335 |
| hsa-let-7d-5p | MIMAT0000065 | miRNA | 100 | 244 | 554 | 170 | 268 | 156 | 416 | 75 | 90 | 402 | 182 | 202 | 590 |
| hsa-let-7e-5p | MIMAT0000066 | miRNA | 100 | 510 | 352 | 116 | 311 | 95 | 333 | 36 | 67 | 276 | 81 | 109 | 223 |
| hsa-let-7f-5p | MIMAT0000067 | miRNA | 100 | 133 | 188 | 66 | 70 | 77 | 127 | 28 | 19 | 118 | 80 | 85 | 250 |
| hsa-let-7g-5p | MIMAT0000414 | miRNA | 100 | 982 | 2902 | 1336 | 1268 | 869 | 1893 | 506 | 309 | 1630 | 669 | 801 | 2632 |
| hsa-let-7i-5p | MIMAT0000415 | miRNA | 67 | 354 | 510 | 1 | 134 | 1 | 320 | 1 | 1 | 149 | 4 | 60 | 408 |
| hsa-miR-1-3p | MIMAT0000416 | miRNA | 100 | 4 | 55 | 59 | 1255 | 24 | 15 | 6 | 606 | 25 | 17 | 16 | 24 |
| hsa-miR-1-5p | MIMAT0031892 | miRNA | 92 | 2 | 3 | 5 | 4 | 2 | 2 | 3 | 1 | 4 | 2 | 3 | 6 |
| hsa-miR-100-5p | MIMAT0000098 | miRNA | 100 | 4038 | 475 | 166 | 168 | 169 | 436 | 100 | 90 | 466 | 154 | 247 | 438 |
| hsa-miR-101-3p | MIMAT0000099 | miRNA | 100 | 3 | 10 | 5 | 3 | 7 | 5 | 4 | 11 | 8 | 10 | 7 | 6 |
| hsa-miR-103a-3p | MIMAT0000101 | miRNA | 100 | 6 | 7 | 5 | 7 | 8 | 7 | 4 | 10 | 6 | 6 | 5 | 9 |
| hsa-miR-105-5p | MIMAT0000102 | miRNA | 100 | 5 | 14 | 14 | 5 | 4 | 6 | 11 | 12 | 4 | 10 | 13 | 8 |
| hsa-miR-106a-5p & miR-17-5p | MIMAT0000103 | miRNA | 100 | 316 | 154 | 113 | 27 | 87 | 129 | 37 | 15 | 136 | 87 | 91 | 238 |
| hsa-miR-106b-5p | MIMAT0000680 | miRNA | 100 | 70 | 120 | 47 | 18 | 40 | 63 | 35 | 30 | 55 | 42 | 54 | 92 |
| hsa-miR-107 | MIMAT0000104 | miRNA | 100 | 87 | 112 | 86 | 53 | 65 | 157 | 49 | 55 | 158 | 77 | 67 | 170 |
| hsa-miR-10a-5p | MIMAT0000253 | miRNA | 100 | 14 | 17 | 29 | 20 | 31 | 36 | 15 | 17 | 24 | 16 | 13 | 33 |
| hsa-miR-10b-5p | MIMAT0000254 | miRNA | 100 | 20 | 155 | 151 | 93 | 113 | 265 | 61 | 52 | 247 | 87 | 123 | 193 |
| hsa-miR-1178-3p | MIMAT0005823 | miRNA | 92 | 1 | 8 | 15 | 9 | 10 | 8 | 3 | 8 | 5 | 7 | 7 | 8 |
| hsa-miR-1180-3p | MIMAT0005825 | miRNA | 100 | 66 | 46 | 25 | 26 | 19 | 60 | 8 | 9 | 37 | 11 | 9 | 35 |
| hsa-miR-1183 | MIMAT0005828 | miRNA | 100 | 7 | 14 | 12 | 6 | 14 | 7 | 14 | 12 | 11 | 18 | 13 | 18 |
| hsa-miR-1185-1-3p | MIMAT0022838 | miRNA | 83 | 5 | 5 | 8 | 5 | 7 | 9 | 1 | 4 | 4 | 3 | 4 | 1 |
| hsa-miR-1185-2-3p | MIMAT0022713 | miRNA | 100 | 24 | 20 | 13 | 17 | 16 | 17 | 8 | 19 | 16 | 24 | 15 | 28 |
| hsa-miR-1185-5p | MIMAT0005798 | miRNA | 100 | 14 | 13 | 15 | 9 | 14 | 12 | 23 | 19 | 11 | 25 | 18 | 18 |
| hsa-miR-1193 | MIMAT0015049 | miRNA | 100 | 6 | 16 | 4 | 15 | 11 | 10 | 7 | 6 | 7 | 10 | 4 | 13 |
| hsa-miR-1197 | MIMAT0005955 | miRNA | 100 | 16 | 16 | 21 | 26 | 24 | 22 | 19 | 34 | 20 | 25 | 18 | 23 |
| hsa-miR-1200 | MIMAT0005863 | miRNA | 100 | 7 | 9 | 14 | 15 | 18 | 15 | 14 | 9 | 10 | 25 | 14 | 16 |
| hsa-miR-1202 | MIMAT0005865 | miRNA | 100 | 3 | 10 | 5 | 5 | 7 | 10 | 7 | 4 | 10 | 5 | 7 | 6 |
| hsa-miR-1203 | MIMAT0005866 | miRNA | 100 | 3 | 9 | 11 | 8 | 11 | 5 | 6 | 6 | 10 | 5 | 5 | 6 |
| hsa-miR-1204 | MIMAT0005868 | miRNA | 83 | 2 | 1 | 2 | 7 | 9 | 4 | 3 | 5 | 4 | 1 | 3 | 2 |
| hsa-miR-1205 | MIMAT0005869 | miRNA | 92 | 6 | 4 | 7 | 2 | 7 | 6 | 4 | 9 | 5 | 1 | 6 | 5 |
| hsa-miR-1206 | MIMAT0005870 | miRNA | 100 | 3 | 8 | 5 | 4 | 10 | 5 | 8 | 3 | 9 | 5 | 8 | 12 |
| hsa-miR-122-5p | MIMAT0000421 | miRNA | 100 | 11 | 15 | 14 | 9 | 10 | 18 | 9 | 14 | 13 | 13 | 6 | 6 |
| hsa-miR-1224-3p | MIMAT0005459 | miRNA | 100 | 6 | 11 | 13 | 4 | 8 | 13 | 11 | 9 | 6 | 15 | 13 | 13 |
| hsa-miR-1224-5p | MIMAT0005458 | miRNA | 92 | 3 | 6 | 7 | 5 | 3 | 5 | 4 | 7 | 7 | 3 | 1 | 2 |
| hsa-miR-1226-3p | MIMAT0005577 | miRNA | 100 | 6 | 10 | 9 | 9 | 5 | 7 | 6 | 14 | 6 | 5 | 8 | 11 |
| hsa-miR-1228-3p | MIMAT0005583 | miRNA | 100 | 8 | 21 | 20 | 13 | 23 | 24 | 21 | 27 | 14 | 22 | 22 | 21 |
| hsa-miR-1233-3p | MIMAT0005588 | miRNA | 100 | 5 | 6 | 3 | 11 | 2 | 4 | 8 | 5 | 5 | 6 | 2 | 4 |

| | | | | | | | | | | | | | | |
|---|---|---|---|---|---|---|---|---|---|---|---|---|---|---|
| hsa-miR-1234-3p | MIMAT0005589 | miRNA | 100 | 6 | 13 | 15 | 13 | 11 | 9 | 14 | 10 | 10 | 13 | 5 | 17 |
| hsa-miR-1236-3p | MIMAT0005591 | miRNA | 100 | 4 | 2 | 13 | 6 | 8 | 8 | 6 | 3 | 3 | 4 | 5 | 3 |
| hsa-miR-124-3p | MIMAT0000422 | miRNA | 92 | 1 | 5 | 9 | 2 | 3 | 4 | 6 | 3 | 4 | 5 | 2 | 2 |
| hsa-miR-1244 | MIMAT0005896 | miRNA | 83 | 1 | 9 | 8 | 1 | 10 | 5 | 8 | 7 | 6 | 4 | 5 | 7 |
| hsa-miR-1245a | MIMAT0005897 | miRNA | 100 | 12 | 23 | 17 | 12 | 16 | 12 | 13 | 12 | 14 | 15 | 19 | 13 |
| hsa-miR-1245b-3p | MIMAT0019951 | miRNA | 92 | 2 | 5 | 6 | 3 | 3 | 5 | 2 | 1 | 6 | 3 | 2 | 5 |
| hsa-miR-1245b-5p | MIMAT0019950 | miRNA | 100 | 6 | 10 | 12 | 7 | 8 | 10 | 2 | 9 | 12 | 10 | 8 | 9 |
| hsa-miR-1246 | MIMAT0005898 | miRNA | 100 | 46 | 67 | 38 | 7 | 33 | 23 | 144 | 13 | 45 | 182 | 20 | 27 |
| hsa-miR-1247-5p | MIMAT0005899 | miRNA | 92 | 4 | 5 | 7 | 7 | 3 | 3 | 6 | 1 | 5 | 4 | 5 | 5 |
| hsa-miR-1248 | MIMAT0005900 | miRNA | 75 | 2 | 8 | 6 | 5 | 8 | 2 | 4 | 1 | 2 | 1 | 2 | 1 |
| hsa-miR-1249-3p | MIMAT0005901 | miRNA | 100 | 11 | 15 | 16 | 14 | 26 | 19 | 23 | 20 | 19 | 20 | 14 | 19 |
| hsa-miR-1249-5p | MIMAT0032029 | miRNA | 92 | 5 | 7 | 8 | 1 | 13 | 5 | 2 | 11 | 5 | 6 | 2 | 7 |
| hsa-miR-1250-5p | MIMAT0005902 | miRNA | 100 | 2 | 5 | 5 | 4 | 2 | 3 | 4 | 3 | 5 | 3 | 2 | 8 |
| hsa-miR-1252-5p | MIMAT0005944 | miRNA | 83 | 1 | 1 | 6 | 4 | 4 | 3 | 4 | 2 | 5 | 7 | 2 | 2 |
| hsa-miR-1253 | MIMAT0005904 | miRNA | 100 | 23 | 15 | 21 | 25 | 9 | 43 | 11 | 17 | 26 | 8 | 17 | 24 |
| hsa-miR-1254 | MIMAT0005905 | miRNA | 100 | 6 | 6 | 4 | 5 | 5 | 7 | 7 | 6 | 3 | 3 | 5 | 8 |
| hsa-miR-1255a | MIMAT0005906 | miRNA | 100 | 11 | 24 | 28 | 22 | 23 | 16 | 20 | 20 | 19 | 15 | 19 | 15 |
| hsa-miR-1255b-5p | MIMAT0005945 | miRNA | 92 | 6 | 9 | 11 | 6 | 3 | 15 | 7 | 7 | 9 | 8 | 1 | 9 |
| hsa-miR-1257 | MIMAT0005908 | miRNA | 100 | 3 | 9 | 15 | 9 | 9 | 7 | 10 | 14 | 10 | 9 | 2 | 11 |
| hsa-miR-1258 | MIMAT0005909 | miRNA | 100 | 10 | 17 | 16 | 12 | 16 | 15 | 13 | 7 | 15 | 6 | 12 | 11 |
| hsa-miR-125a-3p | MIMAT0004602 | miRNA | 100 | 9 | 7 | 7 | 8 | 6 | 10 | 7 | 9 | 9 | 9 | 9 | 9 |
| hsa-miR-125a-5p | MIMAT0000443 | miRNA | 100 | 511 | 342 | 95 | 218 | 104 | 299 | 48 | 46 | 198 | 64 | 70 | 189 |
| hsa-miR-125b-5p | MIMAT0000423 | miRNA | 100 | 5134 | 5573 | 1422 | 2625 | 1412 | 6457 | 729 | 821 | 7179 | 1406 | 2535 | 4765 |
| hsa-miR-126-3p | MIMAT0000445 | miRNA | 100 | 24 | 1782 | 1096 | 751 | 945 | 1495 | 438 | 364 | 1388 | 732 | 1038 | 2413 |
| hsa-miR-1260a | MIMAT0005911 | miRNA | 100 | 76 | 140 | 50 | 55 | 42 | 95 | 23 | 21 | 158 | 52 | 51 | 113 |
| hsa-miR-1260b | MIMAT0015041 | miRNA | 100 | 11 | 7 | 18 | 8 | 11 | 9 | 9 | 12 | 16 | 10 | 4 | 14 |
| hsa-miR-1261 | MIMAT0005913 | miRNA | 92 | 4 | 10 | 10 | 1 | 6 | 11 | 7 | 6 | 6 | 5 | 9 | 9 |
| hsa-miR-1262 | MIMAT0005914 | miRNA | 100 | 8 | 15 | 18 | 13 | 20 | 17 | 22 | 27 | 15 | 16 | 16 | 17 |
| hsa-miR-1264 | MIMAT0005791 | miRNA | 100 | 13 | 16 | 15 | 9 | 12 | 13 | 12 | 9 | 5 | 11 | 6 | 12 |
| hsa-miR-1266-5p | MIMAT0005920 | miRNA | 100 | 5 | 4 | 13 | 9 | 12 | 8 | 10 | 10 | 9 | 6 | 9 | 8 |
| hsa-miR-1268a | MIMAT0005922 | miRNA | 100 | 6 | 9 | 5 | 6 | 4 | 7 | 12 | 3 | 5 | 3 | 5 | 4 |
| hsa-miR-1268b | MIMAT0018925 | miRNA | 100 | 9 | 17 | 26 | 25 | 23 | 21 | 28 | 27 | 25 | 25 | 17 | 17 |
| hsa-miR-1269a | MIMAT0005923 | miRNA | 100 | 6 | 12 | 11 | 6 | 11 | 12 | 5 | 9 | 14 | 8 | 4 | 7 |
| hsa-miR-1269b | MIMAT0019059 | miRNA | 100 | 4 | 7 | 6 | 10 | 10 | 11 | 5 | 14 | 7 | 9 | 2 | 6 |
| hsa-miR-127-3p | MIMAT0000446 | miRNA | 100 | 373 | 524 | 47 | 582 | 32 | 171 | 83 | 386 | 207 | 36 | 52 | 105 |
| hsa-miR-127-5p | MIMAT0004604 | miRNA | 92 | 2 | 7 | 4 | 2 | 1 | 8 | 4 | 3 | 2 | 5 | 5 | 7 |
| hsa-miR-1270 | MIMAT0005924 | miRNA | 100 | 7 | 17 | 13 | 16 | 6 | 23 | 9 | 16 | 9 | 13 | 6 | 12 |
| hsa-miR-1271-3p | MIMAT0022712 | miRNA | 100 | 6 | 6 | 7 | 2 | 2 | 6 | 4 | 3 | 4 | 4 | 3 | 8 |
| hsa-miR-1271-5p | MIMAT0005796 | miRNA | 100 | 3 | 6 | 4 | 8 | 5 | 9 | 8 | 4 | 8 | 2 | 5 | 6 |
| hsa-miR-1272 | MIMAT0005925 | miRNA | 100 | 10 | 13 | 18 | 13 | 19 | 22 | 8 | 20 | 11 | 12 | 9 | 20 |
| hsa-miR-1273c | MIMAT0015017 | miRNA | 100 | 5 | 8 | 12 | 7 | 12 | 10 | 6 | 9 | 15 | 4 | 7 | 4 |
| hsa-miR-1275 | MIMAT0005929 | miRNA | 100 | 8 | 6 | 9 | 5 | 4 | 3 | 7 | 13 | 6 | 3 | 8 | 9 |
| hsa-miR-1276 | MIMAT0005930 | miRNA | 100 | 6 | 8 | 6 | 3 | 6 | 5 | 4 | 4 | 5 | 7 | 5 | 6 |
| hsa-miR-1277-3p | MIMAT0005933 | miRNA | 100 | 3 | 4 | 8 | 4 | 5 | 6 | 7 | 6 | 7 | 8 | 4 | 7 |
| hsa-miR-1278 | MIMAT0005936 | miRNA | 100 | 13 | 14 | 11 | 10 | 20 | 14 | 10 | 13 | 11 | 13 | 17 | 13 |

| | | | | | | | | | | | | | | |
|---|---|---|---|---|---|---|---|---|---|---|---|---|---|---|
| hsa-miR-1279 | MIMAT0005937 | miRNA | 100 | 6 | 3 | 6 | 7 | 9 | 4 | 5 | 8 | 11 | 10 | 8 | 10 |
| hsa-miR-128-1-5p | MIMAT0026477 | miRNA | 92 | 7 | 16 | 6 | 6 | 8 | 3 | 2 | 3 | 5 | 1 | 7 | 5 |
| hsa-miR-128-2-5p | MIMAT0031095 | miRNA | 83 | 1 | 6 | 7 | 4 | 6 | 4 | 9 | 7 | 4 | 6 | 3 | 1 |
| hsa-miR-128-3p | MIMAT0000424 | miRNA | 100 | 11 | 21 | 13 | 15 | 13 | 7 | 9 | 7 | 8 | 11 | 9 | 16 |
| hsa-miR-1281 | MIMAT0005939 | miRNA | 100 | 13 | 10 | 14 | 11 | 15 | 12 | 16 | 15 | 18 | 12 | 11 | 16 |
| hsa-miR-1283 | MIMAT0005799 | miRNA | 33 | 1 | 19 | 1 | 1 | 1 | 35 | 1 | 1 | 6 | 1 | 1 | 19 |
| hsa-miR-1285-3p | MIMAT0005876 | miRNA | 100 | 7 | 12 | 13 | 11 | 11 | 12 | 5 | 13 | 10 | 9 | 4 | 10 |
| hsa-miR-1285-5p | MIMAT0022719 | miRNA | 100 | 20 | 38 | 31 | 37 | 47 | 86 | 48 | 32 | 34 | 27 | 49 | 38 |
| hsa-miR-1286 | MIMAT0005877 | miRNA | 100 | 7 | 20 | 11 | 12 | 24 | 15 | 13 | 19 | 17 | 20 | 17 | 15 |
| hsa-miR-1287-3p | MIMAT0026738 | miRNA | 100 | 6 | 8 | 13 | 7 | 9 | 9 | 6 | 9 | 7 | 7 | 5 | 10 |
| hsa-miR-1287-5p | MIMAT0005878 | miRNA | 100 | 7 | 9 | 13 | 8 | 13 | 10 | 4 | 7 | 16 | 13 | 12 | 12 |
| hsa-miR-1288-3p | MIMAT0005942 | miRNA | 83 | 2 | 5 | 1 | 5 | 4 | 9 | 2 | 4 | 6 | 6 | 1 | 3 |
| hsa-miR-1289 | MIMAT0005879 | miRNA | 100 | 5 | 7 | 11 | 5 | 4 | 5 | 4 | 5 | 3 | 5 | 3 | 8 |
| hsa-miR-129-2-3p | MIMAT0004605 | miRNA | 100 | 12 | 7 | 8 | 5 | 9 | 10 | 8 | 6 | 7 | 8 | 6 | 9 |
| hsa-miR-129-5p | MIMAT0000242 | miRNA | 92 | 1 | 5 | 6 | 2 | 7 | 3 | 5 | 7 | 2 | 6 | 8 | 4 |
| hsa-miR-1290 | MIMAT0005880 | miRNA | 100 | 17 | 19 | 32 | 14 | 29 | 26 | 25 | 28 | 23 | 22 | 15 | 29 |
| hsa-miR-1291 | MIMAT0005881 | miRNA | 92 | 3 | 14 | 8 | 8 | 4 | 7 | 5 | 4 | 7 | 1 | 3 | 6 |
| hsa-miR-1293 | MIMAT0005883 | miRNA | 100 | 10 | 12 | 13 | 8 | 9 | 8 | 4 | 7 | 3 | 10 | 7 | 6 |
| hsa-miR-1295a | MIMAT0005885 | miRNA | 100 | 26 | 32 | 36 | 28 | 29 | 30 | 29 | 26 | 31 | 29 | 29 | 26 |
| hsa-miR-1296-3p | MIMAT0026637 | miRNA | 100 | 13 | 22 | 27 | 15 | 28 | 28 | 23 | 20 | 16 | 26 | 23 | 18 |
| hsa-miR-1296-5p | MIMAT0005794 | miRNA | 100 | 6 | 5 | 10 | 3 | 3 | 2 | 11 | 11 | 2 | 6 | 5 | 6 |
| hsa-miR-1297 | MIMAT0005886 | miRNA | 100 | 9 | 15 | 17 | 7 | 15 | 13 | 9 | 18 | 9 | 9 | 7 | 6 |
| hsa-miR-1298-5p | MIMAT0005800 | miRNA | 100 | 9 | 12 | 8 | 7 | 10 | 6 | 6 | 8 | 10 | 12 | 9 | 11 |
| hsa-miR-1299 | MIMAT0005887 | miRNA | 100 | 6 | 19 | 10 | 13 | 10 | 7 | 14 | 6 | 3 | 11 | 6 | 6 |
| hsa-miR-1301-3p | MIMAT0005797 | miRNA | 92 | 3 | 7 | 6 | 2 | 1 | 5 | 8 | 7 | 5 | 7 | 4 | 3 |
| hsa-miR-1302 | MIMAT0005890 | miRNA | 100 | 20 | 17 | 25 | 23 | 26 | 11 | 19 | 17 | 24 | 34 | 21 | 21 |
| hsa-miR-1303 | MIMAT0005891 | miRNA | 100 | 9 | 6 | 5 | 4 | 2 | 11 | 4 | 8 | 4 | 2 | 2 | 3 |
| hsa-miR-1304-3p | MIMAT0022720 | miRNA | 100 | 4 | 15 | 4 | 6 | 6 | 9 | 9 | 7 | 12 | 12 | 5 | 8 |
| hsa-miR-1304-5p | MIMAT0005892 | miRNA | 100 | 5 | 6 | 10 | 6 | 11 | 17 | 13 | 6 | 5 | 7 | 3 | 9 |
| hsa-miR-1305 | MIMAT0005893 | miRNA | 100 | 10 | 22 | 30 | 20 | 31 | 17 | 26 | 39 | 27 | 28 | 19 | 31 |
| hsa-miR-1306-3p | MIMAT0005950 | miRNA | 100 | 12 | 10 | 9 | 8 | 9 | 18 | 13 | 8 | 9 | 9 | 11 | 4 |
| hsa-miR-1306-5p | MIMAT0022726 | miRNA | 100 | 19 | 12 | 8 | 8 | 4 | 6 | 5 | 3 | 7 | 4 | 6 | 11 |
| hsa-miR-1307-3p | MIMAT0005951 | miRNA | 100 | 8 | 12 | 10 | 9 | 12 | 23 | 6 | 18 | 13 | 12 | 10 | 21 |
| hsa-miR-1307-5p | MIMAT0022727 | miRNA | 100 | 14 | 11 | 15 | 12 | 13 | 20 | 19 | 9 | 19 | 15 | 9 | 13 |
| hsa-miR-130a-3p | MIMAT0000425 | miRNA | 100 | 984 | 1220 | 403 | 512 | 319 | 1233 | 280 | 200 | 855 | 260 | 404 | 806 |
| hsa-miR-130b-3p | MIMAT0000691 | miRNA | 92 | 4 | 2 | 3 | 4 | 5 | 3 | 7 | 1 | 3 | 2 | 9 | 6 |
| hsa-miR-132-3p | MIMAT0000426 | miRNA | 100 | 48 | 54 | 44 | 47 | 48 | 107 | 33 | 23 | 70 | 42 | 38 | 75 |
| hsa-miR-1322 | MIMAT0005953 | miRNA | 100 | 5 | 10 | 6 | 6 | 10 | 15 | 4 | 5 | 6 | 14 | 2 | 7 |
| hsa-miR-1323 | MIMAT0005795 | miRNA | 100 | 16 | 12 | 21 | 14 | 19 | 13 | 19 | 25 | 11 | 22 | 21 | 20 |
| hsa-miR-133a-3p | MIMAT0000427 | miRNA | 100 | 11 | 30 | 37 | 595 | 31 | 23 | 19 | 361 | 39 | 28 | 21 | 27 |
| hsa-miR-133a-5p | MIMAT0026478 | miRNA | 100 | 11 | 15 | 19 | 11 | 15 | 10 | 16 | 16 | 15 | 19 | 19 | 15 |
| hsa-miR-133b | MIMAT0000770 | miRNA | 92 | 3 | 3 | 4 | 9 | 3 | 6 | 5 | 12 | 2 | 4 | 1 | 2 |
| hsa-miR-134-3p | MIMAT0026481 | miRNA | 100 | 18 | 25 | 25 | 16 | 20 | 23 | 23 | 19 | 25 | 30 | 26 | 22 |
| hsa-miR-134-5p & miR-6728-5p | MIMAT0000447 | miRNA | 100 | 3 | 9 | 10 | 5 | 8 | 9 | 5 | 4 | 5 | 6 | 7 | 6 |
| hsa-miR-135a-5p | MIMAT0000428 | miRNA | 100 | 3 | 93 | 9 | 11 | 5 | 5 | 5 | 4 | 8 | 5 | 5 | 8 |

| | | | | | | | | | | | | | | | |
|---|---|---|---|---|---|---|---|---|---|---|---|---|---|---|---|
| hsa-miR-135b-5p | MIMAT0000758 | miRNA | 100 | 26 | 915 | 39 | 51 | 139 | 220 | 49 | 33 | 183 | 160 | 158 | 484 |
| hsa-miR-136-5p | MIMAT0000448 | miRNA | 100 | 63 | 501 | 16 | 50 | 9 | 22 | 9 | 29 | 24 | 20 | 29 | 20 |
| hsa-miR-137 | MIMAT0000429 | miRNA | 100 | 21 | 20 | 23 | 13 | 25 | 12 | 21 | 13 | 16 | 26 | 15 | 19 |
| hsa-miR-138-5p | MIMAT0000430 | miRNA | 100 | 2 | 3 | 12 | 6 | 9 | 15 | 5 | 6 | 6 | 6 | 2 | 4 |
| hsa-miR-139-3p | MIMAT0004552 | miRNA | 100 | 3 | 7 | 10 | 11 | 8 | 13 | 4 | 6 | 13 | 8 | 10 | 12 |
| hsa-miR-139-5p | MIMAT0000250 | miRNA | 92 | 3 | 7 | 11 | 1 | 2 | 11 | 5 | 2 | 6 | 5 | 3 | 5 |
| hsa-miR-140-3p | MIMAT0004597 | miRNA | 100 | 6 | 22 | 12 | 11 | 10 | 13 | 10 | 16 | 14 | 9 | 6 | 18 |
| hsa-miR-140-5p | MIMAT0000431 | miRNA | 100 | 25 | 188 | 31 | 30 | 18 | 63 | 16 | 14 | 45 | 31 | 28 | 67 |
| hsa-miR-141-3p | MIMAT0000432 | miRNA | 100 | 10 | 2372 | 105 | 210 | 144 | 306 | 137 | 55 | 249 | 197 | 177 | 230 |
| hsa-miR-142-3p | MIMAT0000434 | miRNA | 100 | 11 | 1966 | 262 | 71 | 309 | 1089 | 178 | 36 | 544 | 595 | 562 | 960 |
| hsa-miR-142-5p | MIMAT0000433 | miRNA | 83 | 1 | 4 | 4 | 2 | 2 | 3 | 3 | 4 | 5 | 1 | 4 | 2 |
| hsa-miR-143-3p | MIMAT0000435 | miRNA | 100 | 56 | 368 | 20 | 65 | 29 | 37 | 31 | 26 | 38 | 34 | 37 | 56 |
| hsa-miR-144-3p | MIMAT0000436 | miRNA | 100 | 7 | 1776 | 59 | 29 | 43 | 18 | 108 | 27 | 41 | 76 | 21 | 6 |
| hsa-miR-145-5p | MIMAT0000437 | miRNA | 100 | 303 | 2090 | 294 | 1194 | 293 | 686 | 164 | 282 | 605 | 184 | 204 | 430 |
| hsa-miR-1469 | MIMAT0007347 | miRNA | 100 | 4 | 10 | 6 | 11 | 8 | 6 | 4 | 4 | 3 | 8 | 3 | 9 |
| hsa-miR-146a-5p | MIMAT0000449 | miRNA | 100 | 23 | 235 | 37 | 209 | 99 | 305 | 28 | 67 | 73 | 63 | 42 | 100 |
| hsa-miR-146b-3p | MIMAT0004766 | miRNA | 58 | 1 | 1 | 4 | 1 | 7 | 7 | 1 | 1 | 4 | 2 | 5 | 4 |
| hsa-miR-146b-5p | MIMAT0002809 | miRNA | 100 | 4 | 29 | 9 | 6 | 15 | 55 | 10 | 10 | 28 | 16 | 12 | 26 |
| hsa-miR-147a | MIMAT0000251 | miRNA | 100 | 7 | 8 | 11 | 15 | 8 | 13 | 8 | 7 | 9 | 11 | 6 | 13 |
| hsa-miR-147b | MIMAT0004928 | miRNA | 100 | 5 | 11 | 12 | 18 | 17 | 15 | 14 | 15 | 12 | 16 | 15 | 16 |
| hsa-miR-148a-3p | MIMAT0000243 | miRNA | 100 | 14 | 3439 | 72 | 493 | 157 | 159 | 201 | 217 | 139 | 224 | 197 | 246 |
| hsa-miR-148b-3p | MIMAT0000759 | miRNA | 100 | 78 | 604 | 47 | 136 | 68 | 171 | 64 | 48 | 129 | 120 | 107 | 169 |
| hsa-miR-149-5p | MIMAT0000450 | miRNA | 100 | 30 | 25 | 20 | 12 | 15 | 35 | 13 | 15 | 54 | 20 | 21 | 66 |
| hsa-miR-150-5p | MIMAT0000451 | miRNA | 100 | 4 | 535 | 863 | 384 | 729 | 1963 | 203 | 151 | 986 | 319 | 397 | 1645 |
| hsa-miR-151a-3p | MIMAT0000757 | miRNA | 100 | 39 | 78 | 23 | 22 | 20 | 53 | 10 | 11 | 44 | 21 | 25 | 37 |
| hsa-miR-151a-5p | MIMAT0004697 | miRNA | 100 | 7 | 20 | 4 | 5 | 14 | 15 | 6 | 3 | 8 | 11 | 4 | 22 |
| hsa-miR-151b | MIMAT0010214 | miRNA | 100 | 2 | 2 | 5 | 3 | 4 | 4 | 4 | 9 | 3 | 4 | 3 | 9 |
| hsa-miR-152-3p | MIMAT0000438 | miRNA | 100 | 18 | 83 | 14 | 23 | 11 | 21 | 16 | 12 | 14 | 20 | 8 | 19 |
| hsa-miR-152-5p | MIMAT0026479 | miRNA | 67 | 1 | 1 | 3 | 2 | 3 | 2 | 1 | 2 | 3 | 2 | 3 | 1 |
| hsa-miR-153-3p | MIMAT0000439 | miRNA | 92 | 2 | 6 | 8 | 3 | 2 | 6 | 3 | 5 | 4 | 3 | 1 | 10 |
| hsa-miR-1537-3p | MIMAT0007399 | miRNA | 100 | 10 | 19 | 11 | 10 | 10 | 10 | 11 | 11 | 16 | 12 | 17 | 13 |
| hsa-miR-154-5p | MIMAT0000452 | miRNA | 100 | 17 | 109 | 14 | 41 | 12 | 29 | 14 | 23 | 37 | 12 | 14 | 23 |
| hsa-miR-155-5p | MIMAT0000646 | miRNA | 100 | 86 | 16 | 23 | 8 | 11 | 38 | 8 | 12 | 10 | 4 | 9 | 19 |
| hsa-miR-15a-5p | MIMAT0000068 | miRNA | 100 | 629 | 1305 | 355 | 216 | 297 | 678 | 251 | 103 | 438 | 304 | 344 | 690 |
| hsa-miR-15b-5p | MIMAT0000417 | miRNA | 100 | 1585 | 1341 | 1010 | 440 | 649 | 2083 | 333 | 113 | 1826 | 454 | 611 | 1592 |
| hsa-miR-16-5p | MIMAT0000069 | miRNA | 100 | 470 | 1918 | 526 | 367 | 349 | 1143 | 383 | 183 | 954 | 418 | 389 | 1092 |
| hsa-miR-181a-2-3p | MIMAT0004558 | miRNA | 100 | 8 | 28 | 24 | 22 | 23 | 20 | 19 | 28 | 30 | 34 | 15 | 27 |
| hsa-miR-181a-3p | MIMAT0000270 | miRNA | 100 | 27 | 21 | 28 | 14 | 34 | 26 | 15 | 17 | 22 | 23 | 22 | 16 |
| hsa-miR-181a-5p | MIMAT0000256 | miRNA | 100 | 111 | 380 | 131 | 220 | 98 | 570 | 88 | 75 | 436 | 154 | 177 | 322 |
| hsa-miR-181b-2-3p | MIMAT0031893 | miRNA | 100 | 17 | 11 | 14 | 8 | 9 | 12 | 9 | 8 | 11 | 13 | 11 | 17 |
| hsa-miR-181b-5p & miR-181d-5p | MIMAT0000257 | miRNA | 100 | 33 | 16 | 20 | 26 | 12 | 27 | 10 | 12 | 29 | 13 | 14 | 32 |
| hsa-miR-181c-5p | MIMAT0000258 | miRNA | 100 | 14 | 36 | 13 | 31 | 26 | 35 | 13 | 14 | 34 | 12 | 24 | 26 |
| hsa-miR-181d-3p | MIMAT0026608 | miRNA | 100 | 8 | 14 | 26 | 20 | 19 | 8 | 19 | 17 | 22 | 21 | 6 | 19 |
| hsa-miR-182-3p | MIMAT0000260 | miRNA | 100 | 12 | 16 | 15 | 20 | 7 | 8 | 15 | 13 | 19 | 12 | 13 | 21 |
| hsa-miR-182-5p | MIMAT0000259 | miRNA | 100 | 7 | 10 | 8 | 2 | 7 | 5 | 5 | 3 | 4 | 3 | 2 | 10 |

| | | | | | | | | | | | | | | | |
|---|---|---|---|---|---|---|---|---|---|---|---|---|---|---|---|
| hsa-miR-1827 | MIMAT0006767 | miRNA | 100 | 7 | 9 | 14 | 3 | 12 | 13 | 9 | 11 | 7 | 10 | 10 | 11 |
| hsa-miR-183-5p | MIMAT0000261 | miRNA | 100 | 17 | 47 | 38 | 27 | 39 | 41 | 33 | 29 | 45 | 39 | 33 | 66 |
| hsa-miR-184 | MIMAT0000454 | miRNA | 100 | 13 | 24 | 25 | 29 | 27 | 17 | 17 | 29 | 23 | 23 | 18 | 16 |
| hsa-miR-185-5p | MIMAT0000455 | miRNA | 100 | 20 | 25 | 27 | 30 | 24 | 26 | 22 | 27 | 26 | 23 | 28 | 26 |
| hsa-miR-186-5p | MIMAT0000456 | miRNA | 100 | 27 | 111 | 32 | 38 | 33 | 34 | 33 | 29 | 48 | 31 | 25 | 46 |
| hsa-miR-187-3p | MIMAT0000262 | miRNA | 100 | 15 | 29 | 26 | 15 | 19 | 19 | 28 | 27 | 25 | 17 | 22 | 27 |
| hsa-miR-188-3p | MIMAT0004613 | miRNA | 100 | 5 | 10 | 11 | 10 | 14 | 6 | 7 | 10 | 9 | 10 | 12 | 10 |
| hsa-miR-188-5p | MIMAT0000457 | miRNA | 100 | 11 | 20 | 17 | 12 | 14 | 20 | 11 | 12 | 11 | 13 | 8 | 11 |
| hsa-miR-18a-5p | MIMAT0000072 | miRNA | 100 | 25 | 32 | 21 | 18 | 19 | 25 | 20 | 25 | 22 | 24 | 17 | 19 |
| hsa-miR-18b-5p | MIMAT0001412 | miRNA | 100 | 6 | 12 | 9 | 9 | 12 | 12 | 11 | 12 | 14 | 13 | 13 | 15 |
| hsa-miR-1908-3p | MIMAT0026916 | miRNA | 100 | 4 | 6 | 7 | 5 | 8 | 5 | 11 | 7 | 7 | 6 | 3 | 2 |
| hsa-miR-1908-5p | MIMAT0007881 | miRNA | 83 | 4 | 9 | 5 | 4 | 4 | 7 | 6 | 6 | 7 | 1 | 1 | 3 |
| hsa-miR-1909-3p | MIMAT0007883 | miRNA | 100 | 8 | 9 | 7 | 8 | 12 | 5 | 6 | 7 | 10 | 7 | 7 | 5 |
| hsa-miR-190a-3p | MIMAT0026482 | miRNA | 100 | 2 | 10 | 10 | 9 | 4 | 13 | 9 | 7 | 6 | 4 | 7 | 6 |
| hsa-miR-190a-5p | MIMAT0000458 | miRNA | 100 | 12 | 129 | 13 | 15 | 12 | 23 | 25 | 11 | 10 | 27 | 10 | 24 |
| hsa-miR-190b | MIMAT0004929 | miRNA | 100 | 4 | 7 | 10 | 10 | 4 | 6 | 7 | 7 | 9 | 3 | 3 | 5 |
| hsa-miR-191-5p | MIMAT0000440 | miRNA | 100 | 634 | 1358 | 417 | 523 | 419 | 1503 | 247 | 180 | 942 | 245 | 356 | 645 |
| hsa-miR-1910-3p | MIMAT0026917 | miRNA | 83 | 2 | 5 | 5 | 10 | 6 | 6 | 3 | 4 | 1 | 6 | 1 | 6 |
| hsa-miR-1910-5p | MIMAT0007884 | miRNA | 92 | 8 | 46 | 15 | 1 | 10 | 6 | 15 | 7 | 10 | 9 | 4 | 8 |
| hsa-miR-1915-3p | MIMAT0007892 | miRNA | 83 | 1 | 2 | 7 | 4 | 4 | 3 | 7 | 8 | 1 | 8 | 4 | 2 |
| hsa-miR-192-5p | MIMAT0000222 | miRNA | 100 | 11 | 14 | 15 | 9 | 14 | 15 | 8 | 10 | 15 | 7 | 8 | 16 |
| hsa-miR-193a-3p | MIMAT0000459 | miRNA | 100 | 5 | 20 | 9 | 11 | 15 | 18 | 6 | 14 | 11 | 5 | 7 | 14 |
| hsa-miR-193a-5p & miR-193b-5p | MIMAT0004614 | miRNA | 100 | 31 | 32 | 18 | 19 | 9 | 17 | 7 | 12 | 10 | 16 | 4 | 15 |
| hsa-miR-193b-3p | MIMAT0002819 | miRNA | 100 | 3 | 24 | 13 | 6 | 6 | 7 | 4 | 5 | 7 | 9 | 5 | 7 |
| hsa-miR-194-5p | MIMAT0000460 | miRNA | 100 | 6 | 47 | 25 | 14 | 15 | 34 | 8 | 10 | 21 | 13 | 23 | 24 |
| hsa-miR-195-5p | MIMAT0000461 | miRNA | 100 | 6 | 38 | 12 | 23 | 10 | 11 | 12 | 10 | 14 | 15 | 18 | 20 |
| hsa-miR-196a-3p | MIMAT0004562 | miRNA | 100 | 12 | 12 | 19 | 9 | 6 | 14 | 8 | 7 | 7 | 13 | 11 | 9 |
| hsa-miR-196a-5p | MIMAT0000226 | miRNA | 100 | 4 | 20 | 20 | 18 | 14 | 7 | 9 | 12 | 8 | 10 | 6 | 14 |
| hsa-miR-196b-5p | MIMAT0001080 | miRNA | 100 | 3 | 2 | 5 | 4 | 3 | 4 | 4 | 3 | 3 | 5 | 6 | 6 |
| hsa-miR-197-3p | MIMAT0000227 | miRNA | 100 | 24 | 11 | 16 | 8 | 16 | 21 | 12 | 15 | 15 | 11 | 13 | 16 |
| hsa-miR-197-5p | MIMAT0022691 | miRNA | 100 | 16 | 15 | 21 | 17 | 14 | 14 | 23 | 21 | 11 | 18 | 18 | 30 |
| hsa-miR-1972 | MIMAT0009447 | miRNA | 100 | 17 | 20 | 22 | 12 | 16 | 22 | 12 | 19 | 14 | 18 | 9 | 12 |
| hsa-miR-1973 | MIMAT0009448 | miRNA | 100 | 7 | 18 | 23 | 15 | 10 | 19 | 23 | 15 | 8 | 13 | 6 | 16 |
| hsa-miR-1976 | MIMAT0009451 | miRNA | 100 | 9 | 18 | 18 | 18 | 23 | 11 | 19 | 17 | 13 | 26 | 11 | 22 |
| hsa-miR-198 | MIMAT0000228 | miRNA | 100 | 5 | 6 | 7 | 9 | 20 | 15 | 28 | 14 | 6 | 12 | 12 | 8 |
| hsa-miR-199a-3p & miR-199b-3p | MIMAT0000232 | miRNA | 100 | 5259 | 7070 | 1475 | 2472 | 1395 | 3866 | 2111 | 1036 | 4190 | 2448 | 2679 | 3745 |
| hsa-miR-199a-5p | MIMAT0000231 | miRNA | 100 | 259 | 197 | 46 | 96 | 45 | 123 | 69 | 53 | 125 | 97 | 120 | 106 |
| hsa-miR-199b-5p | MIMAT0000263 | miRNA | 100 | 670 | 1481 | 315 | 379 | 228 | 586 | 184 | 196 | 683 | 475 | 833 | 755 |
| hsa-miR-19a-3p | MIMAT0000073 | miRNA | 100 | 46 | 109 | 20 | 8 | 12 | 30 | 13 | 7 | 24 | 26 | 38 | 57 |
| hsa-miR-19b-3p | MIMAT0000074 | miRNA | 100 | 95 | 183 | 60 | 27 | 33 | 70 | 38 | 8 | 65 | 78 | 61 | 101 |
| hsa-miR-200a-3p | MIMAT0000682 | miRNA | 100 | 13 | 838 | 56 | 133 | 107 | 134 | 79 | 60 | 103 | 83 | 78 | 175 |
| hsa-miR-200b-3p | MIMAT0000318 | miRNA | 100 | 3 | 2448 | 236 | 513 | 410 | 814 | 163 | 173 | 703 | 299 | 235 | 742 |
| hsa-miR-200c-3p | MIMAT0000617 | miRNA | 100 | 3 | 641 | 174 | 228 | 217 | 713 | 72 | 64 | 574 | 147 | 216 | 596 |
| hsa-miR-202-3p | MIMAT0002811 | miRNA | 83 | 3 | 6 | 3 | 2 | 3 | 3 | 5 | 1 | 1 | 3 | 7 | 4 |
| hsa-miR-203a-3p | MIMAT0000264 | miRNA | 100 | 2 | 356 | 360 | 6 | 216 | 365 | 150 | 23 | 545 | 408 | 399 | 899 |

| | | | | | | | | | | | | | | | |
|---|---|---|---|---|---|---|---|---|---|---|---|---|---|---|---|
| hsa-miR-203a-5p | MIMAT0031890 | miRNA | 100 | 19 | 25 | 40 | 27 | 29 | 30 | 27 | 36 | 24 | 33 | 32 | 35 |
| hsa-miR-204-5p | MIMAT0000265 | miRNA | 92 | 1 | 8 | 9 | 8 | 7 | 8 | 5 | 6 | 11 | 8 | 6 | 11 |
| hsa-miR-205-5p | MIMAT0000266 | miRNA | 100 | 6 | 4148 | 3618 | 502 | 2956 | 7556 | 1853 | 351 | 7795 | 2808 | 2705 | 9214 |
| hsa-miR-2053 | MIMAT0009978 | miRNA | 100 | 7 | 15 | 17 | 22 | 13 | 30 | 7 | 9 | 14 | 15 | 9 | 14 |
| hsa-miR-206 | MIMAT0000462 | miRNA | 100 | 4 | 26 | 67 | 3037 | 24 | 39 | 2 | 1762 | 114 | 20 | 29 | 7 |
| hsa-miR-208a-3p | MIMAT0000241 | miRNA | 100 | 11 | 22 | 14 | 11 | 17 | 14 | 31 | 16 | 23 | 19 | 15 | 20 |
| hsa-miR-208b-3p | MIMAT0004960 | miRNA | 100 | 14 | 33 | 41 | 55 | 25 | 30 | 31 | 39 | 17 | 23 | 24 | 23 |
| hsa-miR-208b-5p | MIMAT0026722 | miRNA | 100 | 3 | 9 | 8 | 5 | 2 | 7 | 3 | 2 | 5 | 6 | 8 | 5 |
| hsa-miR-20a-5p & miR-20b-5p | MIMAT0000075 | miRNA | 100 | 96 | 113 | 63 | 16 | 42 | 94 | 24 | 11 | 86 | 66 | 61 | 151 |
| hsa-miR-21-5p | MIMAT0000076 | miRNA | 100 | 178 | 538 | 68 | 52 | 112 | 334 | 56 | 18 | 149 | 197 | 139 | 436 |
| hsa-miR-210-3p | MIMAT0000267 | miRNA | 100 | 17 | 29 | 35 | 20 | 25 | 38 | 24 | 17 | 37 | 31 | 18 | 39 |
| hsa-miR-210-5p | MIMAT0026475 | miRNA | 100 | 16 | 28 | 31 | 13 | 23 | 19 | 21 | 19 | 19 | 22 | 31 | 28 |
| hsa-miR-211-3p | MIMAT0022694 | miRNA | 100 | 2 | 2 | 6 | 2 | 7 | 4 | 6 | 6 | 4 | 7 | 3 | 4 |
| hsa-miR-211-5p | MIMAT0000268 | miRNA | 100 | 13 | 22 | 31 | 15 | 28 | 19 | 34 | 25 | 19 | 21 | 26 | 21 |
| hsa-miR-2110 | MIMAT0010133 | miRNA | 100 | 10 | 23 | 14 | 17 | 21 | 22 | 13 | 25 | 29 | 19 | 13 | 26 |
| hsa-miR-2113 | MIMAT0009206 | miRNA | 100 | 6 | 8 | 15 | 10 | 9 | 11 | 11 | 14 | 14 | 20 | 13 | 11 |
| hsa-miR-2116-5p | MIMAT0011160 | miRNA | 100 | 3 | 4 | 4 | 5 | 9 | 2 | 4 | 5 | 3 | 5 | 4 | 5 |
| hsa-miR-2117 | MIMAT0011162 | miRNA | 100 | 12 | 3 | 6 | 3 | 4 | 9 | 8 | 5 | 11 | 7 | 3 | 11 |
| hsa-miR-212-3p | MIMAT0000269 | miRNA | 100 | 13 | 25 | 33 | 18 | 16 | 20 | 25 | 30 | 31 | 26 | 21 | 34 |
| hsa-miR-214-3p | MIMAT0000271 | miRNA | 100 | 28 | 9 | 8 | 28 | 16 | 22 | 13 | 6 | 14 | 7 | 10 | 21 |
| hsa-miR-215-5p | MIMAT0000272 | miRNA | 100 | 4 | 5 | 7 | 5 | 9 | 6 | 6 | 6 | 5 | 3 | 6 | 5 |
| hsa-miR-216a-5p | MIMAT0000273 | miRNA | 100 | 18 | 26 | 29 | 15 | 20 | 25 | 28 | 35 | 38 | 45 | 20 | 34 |
| hsa-miR-216b-5p | MIMAT0004959 | miRNA | 100 | 9 | 24 | 23 | 19 | 7 | 23 | 9 | 11 | 21 | 8 | 7 | 18 |
| hsa-miR-217 | MIMAT0000274 | miRNA | 100 | 8 | 14 | 13 | 19 | 20 | 16 | 13 | 14 | 12 | 11 | 9 | 16 |
| hsa-miR-218-5p | MIMAT0000275 | miRNA | 100 | 138 | 874 | 70 | 114 | 53 | 103 | 66 | 46 | 123 | 99 | 163 | 165 |
| hsa-miR-219a-1-3p | MIMAT0004567 | miRNA | 100 | 5 | 12 | 11 | 6 | 4 | 16 | 8 | 9 | 10 | 7 | 2 | 5 |
| hsa-miR-219a-2-3p | MIMAT0004675 | miRNA | 100 | 4 | 3 | 8 | 3 | 4 | 8 | 4 | 4 | 8 | 5 | 3 | 7 |
| hsa-miR-219a-5p | MIMAT0000276 | miRNA | 92 | 1 | 8 | 3 | 5 | 3 | 4 | 7 | 4 | 10 | 6 | 2 | 4 |
| hsa-miR-219b-3p | MIMAT0019748 | miRNA | 92 | 9 | 5 | 10 | 3 | 4 | 1 | 8 | 16 | 5 | 4 | 7 | 12 |
| hsa-miR-22-3p | MIMAT0000077 | miRNA | 100 | 202 | 392 | 77 | 269 | 64 | 204 | 71 | 63 | 199 | 80 | 53 | 206 |
| hsa-miR-221-3p | MIMAT0000278 | miRNA | 100 | 549 | 50 | 30 | 9 | 27 | 76 | 12 | 16 | 78 | 40 | 39 | 84 |
| hsa-miR-221-5p | MIMAT0004568 | miRNA | 100 | 13 | 5 | 7 | 7 | 8 | 7 | 2 | 4 | 8 | 9 | 6 | 7 |
| hsa-miR-222-3p | MIMAT0000279 | miRNA | 100 | 166 | 148 | 42 | 25 | 28 | 143 | 24 | 10 | 107 | 53 | 37 | 138 |
| hsa-miR-223-3p | MIMAT0000280 | miRNA | 100 | 3 | 799 | 204 | 85 | 217 | 479 | 237 | 48 | 380 | 236 | 216 | 489 |
| hsa-miR-224-5p | MIMAT0000281 | miRNA | 100 | 3 | 11 | 4 | 7 | 10 | 13 | 9 | 12 | 9 | 4 | 6 | 9 |
| hsa-miR-2278 | MIMAT0011778 | miRNA | 92 | 1 | 5 | 4 | 5 | 4 | 5 | 5 | 9 | 7 | 3 | 6 | 5 |
| hsa-miR-23a-3p | MIMAT0000078 | miRNA | 100 | 3386 | 15514 | 5937 | 6261 | 4900 | 13202 | 3960 | 2219 | 10895 | 4576 | 3928 | 9095 |
| hsa-miR-23b-3p | MIMAT0000418 | miRNA | 100 | 57 | 761 | 282 | 234 | 228 | 633 | 194 | 120 | 664 | 309 | 301 | 645 |
| hsa-miR-23c | MIMAT0018000 | miRNA | 100 | 11 | 14 | 17 | 22 | 13 | 13 | 15 | 20 | 12 | 16 | 19 | 24 |
| hsa-miR-24-3p | MIMAT0000080 | miRNA | 100 | 126 | 240 | 81 | 82 | 61 | 308 | 56 | 31 | 219 | 82 | 66 | 193 |
| hsa-miR-25-3p | MIMAT0000081 | miRNA | 100 | 350 | 1084 | 370 | 290 | 265 | 1113 | 235 | 109 | 698 | 224 | 271 | 673 |
| hsa-miR-25-5p | MIMAT0004498 | miRNA | 100 | 20 | 25 | 36 | 29 | 28 | 24 | 22 | 18 | 21 | 27 | 25 | 23 |
| hsa-miR-2682-5p | MIMAT0013517 | miRNA | 100 | 4 | 4 | 5 | 5 | 6 | 11 | 4 | 5 | 2 | 7 | 8 | 6 |
| hsa-miR-26a-5p | MIMAT0000082 | miRNA | 100 | 85 | 209 | 82 | 79 | 75 | 113 | 49 | 39 | 139 | 73 | 101 | 190 |
| hsa-miR-26b-5p | MIMAT0000083 | miRNA | 100 | 40 | 1053 | 229 | 191 | 224 | 302 | 152 | 104 | 258 | 198 | 228 | 456 |

| | | | | | | | | | | | | | | | |
|---|---|---|---|---|---|---|---|---|---|---|---|---|---|---|---|
| hsa-miR-27a-3p | MIMAT0000084 | miRNA | 100 | 19 | 18 | 28 | 15 | 15 | 9 | 19 | 14 | 23 | 25 | 16 | 23 |
| hsa-miR-27b-3p | MIMAT0000419 | miRNA | 100 | 132 | 810 | 421 | 125 | 258 | 769 | 222 | 63 | 815 | 354 | 462 | 931 |
| hsa-miR-28-3p | MIMAT0004502 | miRNA | 100 | 38 | 51 | 25 | 24 | 29 | 40 | 21 | 16 | 32 | 29 | 11 | 42 |
| hsa-miR-28-5p | MIMAT0000085 | miRNA | 100 | 90 | 191 | 60 | 70 | 53 | 73 | 44 | 49 | 68 | 58 | 66 | 90 |
| hsa-miR-296-3p | MIMAT0004679 | miRNA | 100 | 9 | 9 | 11 | 5 | 7 | 15 | 16 | 12 | 6 | 5 | 4 | 9 |
| hsa-miR-296-5p | MIMAT0000690 | miRNA | 100 | 22 | 35 | 14 | 25 | 13 | 45 | 7 | 11 | 38 | 14 | 3 | 24 |
| hsa-miR-297 | MIMAT0004450 | miRNA | 92 | 4 | 2 | 6 | 1 | 6 | 5 | 3 | 4 | 8 | 7 | 6 | 6 |
| hsa-miR-298 | MIMAT0004901 | miRNA | 100 | 2 | 14 | 13 | 8 | 12 | 11 | 16 | 8 | 15 | 9 | 8 | 9 |
| hsa-miR-299-3p | MIMAT0000687 | miRNA | 100 | 40 | 47 | 22 | 23 | 23 | 24 | 19 | 27 | 33 | 14 | 21 | 25 |
| hsa-miR-299-5p | MIMAT0002890 | miRNA | 100 | 169 | 163 | 36 | 274 | 41 | 60 | 48 | 342 | 71 | 44 | 33 | 50 |
| hsa-miR-29a-3p | MIMAT0000086 | miRNA | 100 | 719 | 1971 | 145 | 541 | 95 | 883 | 160 | 135 | 340 | 168 | 130 | 344 |
| hsa-miR-29b-3p | MIMAT0000100 | miRNA | 100 | 6255 | 3432 | 321 | 461 | 424 | 646 | 279 | 112 | 188 | 456 | 350 | 749 |
| hsa-miR-29c-3p | MIMAT0000681 | miRNA | 100 | 39 | 1286 | 63 | 140 | 99 | 125 | 79 | 44 | 123 | 147 | 105 | 215 |
| hsa-miR-300 | MIMAT0004903 | miRNA | 100 | 6 | 9 | 13 | 6 | 8 | 9 | 8 | 4 | 6 | 10 | 6 | 11 |
| hsa-miR-301a-3p | MIMAT0000688 | miRNA | 100 | 37 | 58 | 35 | 25 | 30 | 40 | 23 | 27 | 30 | 42 | 36 | 49 |
| hsa-miR-301a-5p | MIMAT0022696 | miRNA | 100 | 20 | 23 | 25 | 25 | 20 | 24 | 21 | 24 | 25 | 37 | 17 | 22 |
| hsa-miR-301b-3p | MIMAT0004958 | miRNA | 100 | 11 | 3 | 16 | 11 | 17 | 16 | 17 | 18 | 9 | 8 | 11 | 12 |
| hsa-miR-301b-5p | MIMAT0032026 | miRNA | 100 | 15 | 17 | 33 | 18 | 25 | 18 | 22 | 33 | 26 | 37 | 30 | 33 |
| hsa-miR-302a-3p | MIMAT0000684 | miRNA | 100 | 10 | 11 | 14 | 11 | 12 | 11 | 11 | 10 | 6 | 10 | 14 | 12 |
| hsa-miR-302a-5p | MIMAT0000683 | miRNA | 100 | 8 | 12 | 14 | 10 | 9 | 11 | 8 | 9 | 14 | 12 | 10 | 10 |
| hsa-miR-302b-3p | MIMAT0000715 | miRNA | 100 | 16 | 12 | 17 | 12 | 18 | 22 | 12 | 12 | 9 | 11 | 5 | 10 |
| hsa-miR-302c-3p | MIMAT0000717 | miRNA | 100 | 7 | 7 | 5 | 4 | 5 | 6 | 7 | 15 | 10 | 6 | 5 | 6 |
| hsa-miR-302d-3p | MIMAT0000718 | miRNA | 100 | 49 | 48 | 76 | 56 | 44 | 56 | 64 | 75 | 67 | 70 | 60 | 62 |
| hsa-miR-302e | MIMAT0005931 | miRNA | 100 | 9 | 5 | 12 | 3 | 7 | 16 | 8 | 7 | 14 | 8 | 8 | 11 |
| hsa-miR-302f | MIMAT0005932 | miRNA | 100 | 7 | 8 | 14 | 5 | 5 | 16 | 5 | 8 | 6 | 6 | 10 | 10 |
| hsa-miR-3065-3p | MIMAT0015378 | miRNA | 100 | 9 | 21 | 23 | 12 | 24 | 19 | 14 | 16 | 19 | 14 | 15 | 24 |
| hsa-miR-3065-5p | MIMAT0015066 | miRNA | 100 | 6 | 9 | 7 | 8 | 8 | 5 | 8 | 7 | 8 | 6 | 6 | 3 |
| hsa-miR-3074-3p | MIMAT0015027 | miRNA | 100 | 2 | 8 | 8 | 5 | 2 | 6 | 10 | 4 | 4 | 5 | 4 | 6 |
| hsa-miR-30a-3p | MIMAT0000088 | miRNA | 100 | 11 | 32 | 22 | 27 | 15 | 24 | 11 | 23 | 22 | 21 | 17 | 14 |
| hsa-miR-30a-5p | MIMAT0000087 | miRNA | 100 | 7 | 328 | 32 | 71 | 28 | 74 | 28 | 31 | 60 | 40 | 30 | 50 |
| hsa-miR-30b-5p | MIMAT0000420 | miRNA | 100 | 13 | 72 | 31 | 23 | 20 | 39 | 10 | 27 | 48 | 34 | 38 | 62 |
| hsa-miR-30c-5p | MIMAT0000244 | miRNA | 100 | 13 | 108 | 18 | 32 | 22 | 56 | 10 | 25 | 60 | 22 | 24 | 55 |
| hsa-miR-30d-5p | MIMAT0000245 | miRNA | 100 | 11 | 560 | 73 | 211 | 71 | 304 | 63 | 81 | 304 | 78 | 77 | 156 |
| hsa-miR-30e-3p | MIMAT0000693 | miRNA | 100 | 34 | 137 | 67 | 63 | 52 | 73 | 45 | 37 | 56 | 43 | 54 | 78 |
| hsa-miR-30e-5p | MIMAT0000692 | miRNA | 100 | 21 | 328 | 31 | 62 | 44 | 83 | 47 | 57 | 65 | 48 | 60 | 71 |
| hsa-miR-31-5p | MIMAT0000089 | miRNA | 92 | 10 | 3 | 3 | 6 | 7 | 8 | 1 | 6 | 6 | 6 | 5 | 9 |
| hsa-miR-3127-5p | MIMAT0014990 | miRNA | 100 | 4 | 8 | 9 | 5 | 8 | 16 | 7 | 9 | 5 | 4 | 4 | 8 |
| hsa-miR-3130-3p | MIMAT0014994 | miRNA | 100 | 4 | 11 | 8 | 4 | 8 | 10 | 5 | 9 | 10 | 10 | 5 | 7 |
| hsa-miR-3131 | MIMAT0014996 | miRNA | 100 | 8 | 12 | 16 | 11 | 10 | 11 | 11 | 14 | 4 | 14 | 11 | 9 |
| hsa-miR-3136-5p | MIMAT0015003 | miRNA | 100 | 7 | 5 | 6 | 11 | 7 | 6 | 6 | 8 | 7 | 7 | 6 | 8 |
| hsa-miR-3140-3p | MIMAT0015008 | miRNA | 75 | 1 | 1 | 4 | 5 | 1 | 3 | 2 | 3 | 3 | 2 | 4 | 3 |
| hsa-miR-3140-5p | MIMAT0019204 | miRNA | 100 | 5 | 4 | 6 | 3 | 6 | 2 | 4 | 8 | 2 | 10 | 3 | 6 |
| hsa-miR-3144-3p | MIMAT0015015 | miRNA | 100 | 12 | 14 | 25 | 29 | 22 | 32 | 17 | 22 | 16 | 12 | 13 | 14 |
| hsa-miR-3144-5p | MIMAT0015014 | miRNA | 100 | 4 | 9 | 6 | 9 | 3 | 10 | 9 | 7 | 7 | 4 | 5 | 9 |
| hsa-miR-3147 | MIMAT0015019 | miRNA | 100 | 15 | 20 | 27 | 23 | 23 | 21 | 22 | 21 | 17 | 19 | 16 | 25 |

| | | | | | | | | | | | | | | | |
|---|---|---|---|---|---|---|---|---|---|---|---|---|---|---|---|
| hsa-miR-3150b-3p | MIMAT0018194 | miRNA | 92 | 8 | 7 | 6 | 3 | 10 | 3 | 2 | 3 | 4 | 6 | 1 | 4 |
| hsa-miR-3151-5p | MIMAT0015024 | miRNA | 100 | 6 | 9 | 12 | 9 | 4 | 14 | 11 | 9 | 9 | 13 | 3 | 4 |
| hsa-miR-3158-3p | MIMAT0015032 | miRNA | 100 | 6 | 9 | 8 | 4 | 5 | 6 | 2 | 2 | 8 | 5 | 6 | 10 |
| hsa-miR-3161 | MIMAT0015035 | miRNA | 100 | 7 | 19 | 13 | 8 | 5 | 10 | 2 | 7 | 9 | 3 | 5 | 18 |
| hsa-miR-3164 | MIMAT0015038 | miRNA | 83 | 3 | 7 | 9 | 4 | 8 | 5 | 3 | 7 | 1 | 1 | 9 | 6 |
| hsa-miR-3168 | MIMAT0015043 | miRNA | 100 | 15 | 26 | 25 | 25 | 35 | 19 | 28 | 26 | 24 | 28 | 19 | 33 |
| hsa-miR-3179 | MIMAT0015056 | miRNA | 100 | 2 | 5 | 4 | 5 | 7 | 4 | 2 | 5 | 8 | 8 | 4 | 3 |
| hsa-miR-3180 | MIMAT0018178 | miRNA | 100 | 7 | 10 | 16 | 16 | 9 | 11 | 7 | 11 | 14 | 13 | 6 | 8 |
| hsa-miR-3180-3p | MIMAT0015058 | miRNA | 100 | 3 | 5 | 11 | 3 | 7 | 5 | 6 | 6 | 3 | 7 | 5 | 12 |
| hsa-miR-3180-5p | MIMAT0015057 | miRNA | 100 | 6 | 7 | 6 | 5 | 8 | 6 | 10 | 8 | 5 | 9 | 3 | 4 |
| hsa-miR-3182 | MIMAT0015062 | miRNA | 100 | 16 | 10 | 12 | 8 | 8 | 5 | 6 | 9 | 9 | 14 | 9 | 11 |
| hsa-miR-3185 | MIMAT0015065 | miRNA | 100 | 7 | 6 | 7 | 5 | 10 | 8 | 10 | 3 | 8 | 6 | 6 | 11 |
| hsa-miR-3190-3p | MIMAT0022839 | miRNA | 92 | 1 | 5 | 6 | 2 | 6 | 4 | 3 | 3 | 4 | 4 | 2 | 6 |
| hsa-miR-3192-5p | MIMAT0015076 | miRNA | 92 | 4 | 5 | 6 | 5 | 1 | 2 | 8 | 5 | 8 | 2 | 3 | 6 |
| hsa-miR-3195 | MIMAT0015079 | miRNA | 83 | 3 | 8 | 5 | 3 | 6 | 11 | 10 | 1 | 7 | 7 | 1 | 6 |
| hsa-miR-3196 | MIMAT0015080 | miRNA | 92 | 2 | 6 | 9 | 5 | 3 | 5 | 5 | 1 | 5 | 5 | 3 | 6 |
| hsa-miR-32-5p | MIMAT0000090 | miRNA | 100 | 18 | 366 | 24 | 19 | 23 | 27 | 22 | 13 | 24 | 44 | 36 | 43 |
| hsa-miR-3202 | MIMAT0015089 | miRNA | 100 | 8 | 7 | 10 | 7 | 3 | 8 | 3 | 5 | 7 | 6 | 4 | 3 |
| hsa-miR-320a | MIMAT0000510 | miRNA | 100 | 4 | 9 | 10 | 9 | 7 | 14 | 8 | 5 | 9 | 7 | 8 | 4 |
| hsa-miR-320b | MIMAT0005792 | miRNA | 100 | 2 | 5 | 3 | 3 | 2 | 6 | 4 | 4 | 3 | 5 | 2 | 6 |
| hsa-miR-320c | MIMAT0005793 | miRNA | 100 | 2 | 7 | 12 | 5 | 12 | 9 | 7 | 5 | 7 | 6 | 3 | 10 |
| hsa-miR-320d | MIMAT0006764 | miRNA | 100 | 2 | 8 | 13 | 4 | 5 | 9 | 5 | 4 | 4 | 4 | 4 | 2 |
| hsa-miR-320e | MIMAT0015072 | miRNA | 100 | 11 | 27 | 14 | 22 | 18 | 33 | 7 | 11 | 14 | 6 | 9 | 18 |
| hsa-miR-323a-3p | MIMAT0000755 | miRNA | 100 | 44 | 22 | 13 | 13 | 12 | 10 | 15 | 12 | 18 | 4 | 12 | 11 |
| hsa-miR-323a-5p | MIMAT0004696 | miRNA | 83 | 1 | 3 | 2 | 1 | 3 | 4 | 8 | 6 | 3 | 2 | 4 | 6 |
| hsa-miR-323b-3p | MIMAT0015050 | miRNA | 100 | 7 | 15 | 13 | 9 | 14 | 13 | 10 | 11 | 2 | 11 | 7 | 6 |
| hsa-miR-323b-5p | MIMAT0001630 | miRNA | 100 | 3 | 4 | 3 | 2 | 10 | 7 | 2 | 5 | 3 | 4 | 5 | 2 |
| hsa-miR-324-3p | MIMAT0000762 | miRNA | 100 | 20 | 24 | 19 | 15 | 24 | 16 | 22 | 20 | 24 | 27 | 16 | 25 |
| hsa-miR-324-5p | MIMAT0000761 | miRNA | 100 | 15 | 35 | 5 | 13 | 8 | 34 | 5 | 7 | 29 | 4 | 4 | 17 |
| hsa-miR-325 | MIMAT0000771 | miRNA | 100 | 5 | 8 | 13 | 8 | 7 | 24 | 14 | 12 | 7 | 6 | 6 | 6 |
| hsa-miR-326 | MIMAT0000756 | miRNA | 100 | 6 | 6 | 12 | 4 | 6 | 3 | 5 | 3 | 2 | 6 | 4 | 2 |
| hsa-miR-328-3p | MIMAT0000752 | miRNA | 92 | 7 | 6 | 10 | 5 | 6 | 6 | 2 | 5 | 4 | 4 | 1 | 5 |
| hsa-miR-328-5p | MIMAT0026486 | miRNA | 100 | 14 | 16 | 22 | 17 | 20 | 16 | 11 | 16 | 17 | 13 | 13 | 20 |
| hsa-miR-329-3p | MIMAT0001629 | miRNA | 100 | 19 | 12 | 10 | 7 | 4 | 10 | 11 | 16 | 5 | 6 | 3 | 5 |
| hsa-miR-329-5p | MIMAT0026555 | miRNA | 100 | 8 | 11 | 7 | 5 | 8 | 6 | 10 | 4 | 10 | 2 | 8 | 10 |
| hsa-miR-330-3p | MIMAT0000751 | miRNA | 100 | 5 | 7 | 9 | 4 | 5 | 5 | 3 | 4 | 10 | 5 | 7 | 12 |
| hsa-miR-330-5p | MIMAT0004693 | miRNA | 100 | 10 | 13 | 12 | 15 | 10 | 11 | 2 | 7 | 10 | 7 | 5 | 9 |
| hsa-miR-331-3p | MIMAT0000760 | miRNA | 100 | 21 | 18 | 11 | 14 | 13 | 26 | 14 | 11 | 26 | 12 | 11 | 29 |
| hsa-miR-331-5p | MIMAT0004700 | miRNA | 100 | 4 | 5 | 9 | 7 | 3 | 7 | 4 | 2 | 6 | 4 | 4 | 5 |
| hsa-miR-335-5p | MIMAT0000765 | miRNA | 100 | 25 | 69 | 27 | 23 | 28 | 37 | 36 | 18 | 37 | 22 | 38 | 44 |
| hsa-miR-337-3p | MIMAT0000754 | miRNA | 100 | 84 | 85 | 24 | 31 | 29 | 36 | 21 | 62 | 34 | 37 | 31 | 31 |
| hsa-miR-337-5p | MIMAT0004695 | miRNA | 100 | 147 | 66 | 38 | 34 | 28 | 24 | 21 | 61 | 38 | 29 | 38 | 30 |
| hsa-miR-338-5p | MIMAT0004701 | miRNA | 100 | 11 | 13 | 22 | 11 | 20 | 14 | 16 | 19 | 12 | 15 | 18 | 20 |
| hsa-miR-339-3p | MIMAT0004702 | miRNA | 100 | 4 | 9 | 11 | 3 | 6 | 4 | 2 | 5 | 6 | 5 | 4 | 6 |
| hsa-miR-339-5p | MIMAT0000764 | miRNA | 100 | 5 | 5 | 3 | 5 | 14 | 8 | 4 | 9 | 13 | 7 | 2 | 7 |

| | | | | | | | | | | | | | | | |
|---|---|---|---|---|---|---|---|---|---|---|---|---|---|---|---|
| hsa-miR-33a-5p | MIMAT0000091 | miRNA | 100 | 6 | 27 | 11 | 7 | 10 | 8 | 11 | 4 | 3 | 16 | 8 | 11 |
| hsa-miR-33b-5p | MIMAT0003301 | miRNA | 100 | 18 | 20 | 31 | 13 | 26 | 24 | 25 | 26 | 10 | 22 | 21 | 31 |
| hsa-miR-340-5p | MIMAT0004692 | miRNA | 100 | 35 | 189 | 37 | 53 | 37 | 51 | 27 | 23 | 38 | 33 | 38 | 56 |
| hsa-miR-342-3p | MIMAT0000753 | miRNA | 100 | 32 | 205 | 108 | 70 | 107 | 337 | 48 | 44 | 277 | 69 | 93 | 262 |
| hsa-miR-342-5p | MIMAT0004694 | miRNA | 92 | 3 | 11 | 7 | 5 | 6 | 7 | 5 | 10 | 8 | 2 | 3 | 1 |
| hsa-miR-345-3p | MIMAT0022698 | miRNA | 100 | 19 | 16 | 21 | 13 | 31 | 14 | 16 | 25 | 16 | 20 | 29 | 24 |
| hsa-miR-345-5p | MIMAT0000772 | miRNA | 100 | 11 | 17 | 4 | 7 | 9 | 10 | 8 | 11 | 10 | 5 | 12 | 14 |
| hsa-miR-346 | MIMAT0000773 | miRNA | 100 | 6 | 7 | 12 | 7 | 12 | 5 | 7 | 6 | 9 | 7 | 6 | 6 |
| hsa-miR-34a-5p | MIMAT0000255 | miRNA | 100 | 137 | 318 | 80 | 89 | 57 | 227 | 57 | 40 | 203 | 70 | 55 | 181 |
| hsa-miR-34b-3p | MIMAT0004676 | miRNA | 100 | 3 | 6 | 7 | 9 | 8 | 7 | 12 | 10 | 9 | 9 | 6 | 9 |
| hsa-miR-34c-3p | MIMAT0004677 | miRNA | 100 | 5 | 11 | 11 | 8 | 9 | 7 | 7 | 8 | 7 | 7 | 7 | 7 |
| hsa-miR-34c-5p | MIMAT0000686 | miRNA | 100 | 7 | 7 | 10 | 10 | 9 | 24 | 3 | 8 | 7 | 9 | 6 | 10 |
| hsa-miR-3605-3p | MIMAT0017982 | miRNA | 100 | 6 | 6 | 6 | 4 | 9 | 3 | 2 | 2 | 5 | 7 | 10 | 7 |
| hsa-miR-3605-5p | MIMAT0017981 | miRNA | 100 | 16 | 24 | 17 | 13 | 20 | 11 | 22 | 20 | 10 | 23 | 14 | 18 |
| hsa-miR-361-3p | MIMAT0004682 | miRNA | 100 | 8 | 81 | 18 | 26 | 16 | 47 | 14 | 14 | 34 | 9 | 13 | 32 |
| hsa-miR-361-5p | MIMAT0000703 | miRNA | 100 | 60 | 119 | 45 | 48 | 36 | 111 | 24 | 23 | 102 | 35 | 47 | 98 |
| hsa-miR-3613-3p | MIMAT0017991 | miRNA | 100 | 9 | 22 | 24 | 10 | 25 | 26 | 16 | 16 | 11 | 21 | 20 | 17 |
| hsa-miR-3613-5p | MIMAT0017990 | miRNA | 100 | 3 | 4 | 2 | 4 | 3 | 6 | 7 | 5 | 3 | 4 | 3 | 6 |
| hsa-miR-3614-3p | MIMAT0017993 | miRNA | 92 | 6 | 8 | 9 | 7 | 3 | 7 | 4 | 6 | 4 | 6 | 1 | 6 |
| hsa-miR-3614-5p | MIMAT0017992 | miRNA | 100 | 19 | 18 | 33 | 24 | 34 | 31 | 30 | 32 | 26 | 37 | 25 | 32 |
| hsa-miR-3615 | MIMAT0017994 | miRNA | 100 | 7 | 6 | 6 | 4 | 11 | 4 | 4 | 7 | 2 | 7 | 13 | 3 |
| hsa-miR-362-3p | MIMAT0004683 | miRNA | 100 | 4 | 18 | 5 | 5 | 2 | 4 | 3 | 6 | 6 | 8 | 5 | 5 |
| hsa-miR-362-5p | MIMAT0000705 | miRNA | 100 | 18 | 46 | 31 | 22 | 30 | 44 | 20 | 27 | 30 | 28 | 20 | 47 |
| hsa-miR-363-3p | MIMAT0000707 | miRNA | 100 | 19 | 82 | 41 | 36 | 37 | 43 | 34 | 23 | 35 | 31 | 26 | 46 |
| hsa-miR-363-5p | MIMAT0003385 | miRNA | 100 | 10 | 11 | 15 | 7 | 4 | 7 | 8 | 9 | 5 | 7 | 6 | 12 |
| hsa-miR-365a-3p & miR-365b-3p | MIMAT0000710 | miRNA | 100 | 43 | 70 | 36 | 58 | 32 | 40 | 28 | 30 | 57 | 52 | 37 | 68 |
| hsa-miR-365b-5p | MIMAT0022833 | miRNA | 100 | 5 | 14 | 12 | 12 | 10 | 10 | 9 | 11 | 7 | 11 | 6 | 5 |
| hsa-miR-367-3p | MIMAT0000719 | miRNA | 100 | 6 | 8 | 7 | 10 | 3 | 9 | 6 | 7 | 6 | 7 | 4 | 7 |
| hsa-miR-369-3p | MIMAT0000721 | miRNA | 100 | 26 | 22 | 33 | 26 | 32 | 22 | 33 | 25 | 24 | 31 | 32 | 21 |
| hsa-miR-369-5p | MIMAT0001621 | miRNA | 92 | 6 | 11 | 8 | 7 | 10 | 7 | 7 | 14 | 1 | 11 | 9 | 13 |
| hsa-miR-3690 | MIMAT0018119 | miRNA | 100 | 8 | 10 | 9 | 4 | 6 | 8 | 7 | 5 | 5 | 13 | 5 | 13 |
| hsa-miR-370-3p | MIMAT0000722 | miRNA | 100 | 33 | 20 | 28 | 21 | 33 | 17 | 25 | 24 | 29 | 31 | 15 | 20 |
| hsa-miR-370-5p | MIMAT0026483 | miRNA | 100 | 5 | 8 | 5 | 4 | 8 | 10 | 4 | 3 | 4 | 7 | 4 | 8 |
| hsa-miR-371a-5p | MIMAT0004687 | miRNA | 100 | 11 | 7 | 15 | 14 | 10 | 11 | 20 | 11 | 8 | 9 | 5 | 10 |
| hsa-miR-371b-5p | MIMAT0019892 | miRNA | 100 | 5 | 3 | 4 | 7 | 2 | 5 | 5 | 2 | 9 | 7 | 5 | 8 |
| hsa-miR-372-3p | MIMAT0000724 | miRNA | 100 | 2 | 3 | 8 | 4 | 5 | 10 | 2 | 5 | 3 | 6 | 2 | 7 |
| hsa-miR-373-3p | MIMAT0000726 | miRNA | 92 | 4 | 10 | 12 | 1 | 6 | 10 | 4 | 9 | 5 | 10 | 7 | 5 |
| hsa-miR-374a-3p | MIMAT0004688 | miRNA | 100 | 5 | 6 | 10 | 6 | 3 | 16 | 4 | 2 | 11 | 5 | 5 | 8 |
| hsa-miR-374a-5p | MIMAT0000727 | miRNA | 100 | 491 | 1323 | 297 | 138 | 305 | 332 | 178 | 67 | 297 | 331 | 299 | 682 |
| hsa-miR-374b-5p | MIMAT0004955 | miRNA | 100 | 18 | 52 | 15 | 19 | 15 | 28 | 5 | 11 | 25 | 11 | 9 | 31 |
| hsa-miR-374c-5p | MIMAT0018443 | miRNA | 100 | 5 | 11 | 11 | 7 | 6 | 7 | 5 | 4 | 6 | 8 | 7 | 9 |
| hsa-miR-375 | MIMAT0000728 | miRNA | 100 | 4 | 148 | 12 | 70 | 12 | 9 | 15 | 17 | 32 | 18 | 10 | 41 |
| hsa-miR-376a-2-5p | MIMAT0022928 | miRNA | 100 | 5 | 15 | 10 | 7 | 10 | 10 | 13 | 11 | 12 | 11 | 8 | 14 |
| hsa-miR-376a-3p | MIMAT0000729 | miRNA | 100 | 177 | 305 | 25 | 53 | 25 | 57 | 35 | 63 | 62 | 41 | 56 | 50 |
| hsa-miR-376b-3p | MIMAT0002172 | miRNA | 100 | 31 | 11 | 10 | 2 | 7 | 7 | 3 | 5 | 7 | 6 | 4 | 8 |

| | | | | | | | | | | | | | | | |
|---|---|---|---|---|---|---|---|---|---|---|---|---|---|---|---|
| hsa-miR-376c-3p | MIMAT0000720 | miRNA | 100 | 77 | 133 | 23 | 29 | 10 | 21 | 17 | 36 | 20 | 23 | 19 | 25 |
| hsa-miR-376c-5p | MIMAT0022861 | miRNA | 100 | 7 | 10 | 14 | 11 | 8 | 15 | 10 | 13 | 9 | 6 | 10 | 18 |
| hsa-miR-377-3p | MIMAT0000730 | miRNA | 100 | 84 | 269 | 27 | 56 | 12 | 26 | 19 | 42 | 33 | 34 | 29 | 32 |
| hsa-miR-378b | MIMAT0014999 | miRNA | 92 | 3 | 6 | 4 | 4 | 6 | 6 | 9 | 3 | 3 | 4 | 3 | 1 |
| hsa-miR-378c | MIMAT0016847 | miRNA | 100 | 10 | 4 | 3 | 8 | 3 | 8 | 3 | 6 | 8 | 4 | 4 | 7 |
| hsa-miR-378d | MIMAT0018926 | miRNA | 100 | 13 | 28 | 28 | 19 | 30 | 23 | 27 | 18 | 23 | 32 | 15 | 28 |
| hsa-miR-378e | MIMAT0018927 | miRNA | 100 | 18 | 30 | 20 | 15 | 21 | 35 | 18 | 21 | 27 | 17 | 21 | 28 |
| hsa-miR-378f | MIMAT0018932 | miRNA | 100 | 13 | 20 | 28 | 28 | 29 | 22 | 25 | 36 | 24 | 25 | 22 | 26 |
| hsa-miR-378g | MIMAT0018937 | miRNA | 100 | 8 | 50 | 27 | 44 | 32 | 43 | 24 | 32 | 44 | 31 | 21 | 43 |
| hsa-miR-378h | MIMAT0018984 | miRNA | 100 | 12 | 17 | 21 | 20 | 18 | 22 | 19 | 25 | 18 | 16 | 12 | 14 |
| hsa-miR-378i | MIMAT0019074 | miRNA | 100 | 12 | 175 | 89 | 281 | 57 | 204 | 27 | 134 | 188 | 66 | 63 | 138 |
| hsa-miR-379-5p | MIMAT0000733 | miRNA | 100 | 206 | 208 | 18 | 94 | 21 | 44 | 31 | 82 | 55 | 21 | 28 | 25 |
| hsa-miR-380-3p | MIMAT0000735 | miRNA | 100 | 7 | 10 | 18 | 5 | 8 | 14 | 13 | 3 | 14 | 10 | 6 | 12 |
| hsa-miR-381-3p | MIMAT0000736 | miRNA | 100 | 30 | 77 | 8 | 33 | 11 | 14 | 9 | 62 | 22 | 10 | 12 | 8 |
| hsa-miR-381-5p | MIMAT0022862 | miRNA | 100 | 4 | 8 | 9 | 7 | 13 | 5 | 6 | 17 | 6 | 5 | 7 | 5 |
| hsa-miR-382-3p | MIMAT0022697 | miRNA | 100 | 4 | 7 | 9 | 8 | 7 | 7 | 8 | 9 | 13 | 5 | 8 | 8 |
| hsa-miR-382-5p | MIMAT0000737 | miRNA | 100 | 197 | 98 | 16 | 58 | 8 | 30 | 16 | 55 | 35 | 15 | 31 | 25 |
| hsa-miR-383-5p | MIMAT0000738 | miRNA | 92 | 1 | 4 | 12 | 5 | 7 | 8 | 6 | 6 | 6 | 6 | 6 | 2 |
| hsa-miR-384 | MIMAT0001075 | miRNA | 100 | 7 | 8 | 6 | 5 | 7 | 4 | 7 | 5 | 5 | 6 | 6 | 8 |
| hsa-miR-3916 | MIMAT0018190 | miRNA | 92 | 6 | 10 | 7 | 9 | 9 | 6 | 1 | 9 | 5 | 7 | 9 | 4 |
| hsa-miR-3918 | MIMAT0018192 | miRNA | 100 | 2 | 7 | 5 | 7 | 5 | 5 | 7 | 5 | 4 | 5 | 5 | 7 |
| hsa-miR-3928-3p | MIMAT0018205 | miRNA | 100 | 12 | 8 | 10 | 14 | 10 | 9 | 6 | 6 | 14 | 11 | 5 | 5 |
| hsa-miR-3934-5p | MIMAT0018349 | miRNA | 100 | 16 | 7 | 14 | 2 | 5 | 16 | 10 | 14 | 13 | 7 | 6 | 9 |
| hsa-miR-409-3p | MIMAT0001639 | miRNA | 100 | 47 | 14 | 9 | 9 | 5 | 10 | 5 | 9 | 12 | 5 | 4 | 10 |
| hsa-miR-409-5p | MIMAT0001638 | miRNA | 100 | 11 | 7 | 5 | 4 | 3 | 3 | 2 | 3 | 5 | 2 | 2 | 4 |
| hsa-miR-410-3p | MIMAT0002171 | miRNA | 100 | 10 | 13 | 10 | 17 | 7 | 16 | 6 | 11 | 9 | 7 | 12 | 5 |
| hsa-miR-411-5p | MIMAT0003329 | miRNA | 100 | 102 | 124 | 22 | 49 | 13 | 22 | 10 | 60 | 40 | 12 | 10 | 14 |
| hsa-miR-412-3p | MIMAT0002170 | miRNA | 100 | 10 | 7 | 24 | 12 | 11 | 21 | 20 | 14 | 9 | 24 | 6 | 9 |
| hsa-miR-421 | MIMAT0003339 | miRNA | 100 | 57 | 50 | 66 | 32 | 53 | 66 | 38 | 32 | 46 | 53 | 49 | 68 |
| hsa-miR-422a | MIMAT0001339 | miRNA | 92 | 3 | 10 | 6 | 12 | 11 | 15 | 4 | 6 | 7 | 6 | 1 | 7 |
| hsa-miR-423-3p | MIMAT0001340 | miRNA | 100 | 36 | 29 | 17 | 24 | 14 | 33 | 8 | 5 | 33 | 11 | 7 | 28 |
| hsa-miR-423-5p | MIMAT0004748 | miRNA | 100 | 26 | 19 | 23 | 23 | 14 | 30 | 11 | 8 | 14 | 12 | 10 | 34 |
| hsa-miR-424-5p | MIMAT0001341 | miRNA | 100 | 169 | 719 | 51 | 126 | 48 | 156 | 94 | 118 | 154 | 133 | 144 | 152 |
| hsa-miR-425-5p | MIMAT0003393 | miRNA | 100 | 7 | 22 | 13 | 6 | 10 | 37 | 10 | 5 | 28 | 11 | 13 | 24 |
| hsa-miR-4284 | MIMAT0016915 | miRNA | 100 | 11 | 12 | 22 | 10 | 12 | 13 | 13 | 26 | 12 | 22 | 11 | 18 |
| hsa-miR-4286 | MIMAT0016916 | miRNA | 100 | 35 | 61 | 43 | 32 | 40 | 34 | 34 | 40 | 24 | 28 | 20 | 26 |
| hsa-miR-429 | MIMAT0001536 | miRNA | 100 | 3 | 48 | 21 | 12 | 17 | 20 | 8 | 7 | 15 | 11 | 12 | 40 |
| hsa-miR-431-5p | MIMAT0001625 | miRNA | 92 | 27 | 8 | 7 | 4 | 3 | 5 | 4 | 10 | 7 | 5 | 1 | 8 |
| hsa-miR-432-5p | MIMAT0002814 | miRNA | 100 | 83 | 16 | 16 | 23 | 11 | 8 | 6 | 11 | 14 | 7 | 10 | 15 |
| hsa-miR-433-3p | MIMAT0001627 | miRNA | 100 | 26 | 18 | 4 | 22 | 3 | 9 | 10 | 13 | 10 | 3 | 2 | 10 |
| hsa-miR-433-5p | MIMAT0026554 | miRNA | 100 | 6 | 9 | 9 | 4 | 6 | 12 | 5 | 13 | 12 | 8 | 10 | 9 |
| hsa-miR-4421 | MIMAT0018934 | miRNA | 100 | 6 | 14 | 21 | 13 | 10 | 6 | 21 | 16 | 14 | 15 | 8 | 9 |
| hsa-miR-4425 | MIMAT0018940 | miRNA | 92 | 1 | 9 | 6 | 7 | 6 | 10 | 8 | 8 | 10 | 6 | 2 | 10 |
| hsa-miR-4431 | MIMAT0018947 | miRNA | 100 | 8 | 12 | 14 | 12 | 14 | 9 | 7 | 8 | 14 | 6 | 10 | 7 |
| hsa-miR-4435 | MIMAT0018951 | miRNA | 92 | 5 | 9 | 5 | 7 | 6 | 5 | 3 | 8 | 10 | 4 | 1 | 4 |

| | | | | | | | | | | | | | | | |
|---|---|---|---|---|---|---|---|---|---|---|---|---|---|---|---|
| hsa-miR-4443 | MIMAT0018961 | miRNA | 100 | 41 | 75 | 53 | 32 | 38 | 70 | 19 | 11 | 37 | 26 | 16 | 44 |
| hsa-miR-4448 | MIMAT0018967 | miRNA | 100 | 4 | 5 | 7 | 3 | 8 | 7 | 5 | 8 | 7 | 5 | 3 | 5 |
| hsa-miR-4451 | MIMAT0018973 | miRNA | 100 | 3 | 7 | 10 | 4 | 7 | 12 | 9 | 11 | 12 | 9 | 11 | 3 |
| hsa-miR-4454 & miR-7975 | MIMAT0018976 | miRNA | 100 | 7121 | 39044 | 16199 | 15807 | 6405 | 16961 | 4028 | 3938 | 14250 | 8654 | 3714 | 14685 |
| hsa-miR-4455 | MIMAT0018977 | miRNA | 100 | 5 | 6 | 6 | 7 | 11 | 7 | 2 | 7 | 14 | 7 | 5 | 6 |
| hsa-miR-4458 | MIMAT0018980 | miRNA | 100 | 4 | 6 | 10 | 2 | 6 | 7 | 2 | 5 | 3 | 9 | 7 | 3 |
| hsa-miR-4461 | MIMAT0018983 | miRNA | 100 | 15 | 20 | 27 | 13 | 14 | 9 | 16 | 17 | 12 | 27 | 18 | 17 |
| hsa-miR-448 | MIMAT0001532 | miRNA | 100 | 16 | 11 | 25 | 16 | 14 | 12 | 17 | 21 | 7 | 14 | 8 | 17 |
| hsa-miR-4485-3p | MIMAT0019019 | miRNA | 100 | 11 | 12 | 19 | 11 | 21 | 17 | 21 | 16 | 10 | 17 | 11 | 21 |
| hsa-miR-4488 | MIMAT0019022 | miRNA | 100 | 8 | 13 | 25 | 11 | 20 | 16 | 22 | 14 | 20 | 23 | 21 | 19 |
| hsa-miR-449a | MIMAT0001541 | miRNA | 100 | 7 | 10 | 11 | 11 | 9 | 9 | 10 | 9 | 8 | 7 | 2 | 18 |
| hsa-miR-449b-5p | MIMAT0003327 | miRNA | 100 | 18 | 17 | 11 | 11 | 24 | 16 | 15 | 26 | 15 | 18 | 8 | 17 |
| hsa-miR-449c-5p | MIMAT0010251 | miRNA | 92 | 5 | 1 | 8 | 3 | 10 | 4 | 6 | 10 | 5 | 2 | 5 | 2 |
| hsa-miR-450a-1-3p | MIMAT0022700 | miRNA | 100 | 2 | 14 | 6 | 9 | 8 | 3 | 6 | 5 | 5 | 7 | 4 | 7 |
| hsa-miR-450a-2-3p | MIMAT0031074 | miRNA | 100 | 10 | 18 | 28 | 19 | 20 | 26 | 22 | 20 | 21 | 20 | 17 | 19 |
| hsa-miR-450a-5p | MIMAT0001545 | miRNA | 100 | 87 | 93 | 21 | 32 | 10 | 37 | 9 | 17 | 29 | 39 | 58 | 48 |
| hsa-miR-450b-3p | MIMAT0004910 | miRNA | 100 | 4 | 10 | 8 | 10 | 8 | 5 | 2 | 7 | 7 | 8 | 3 | 8 |
| hsa-miR-450b-5p | MIMAT0004909 | miRNA | 100 | 49 | 60 | 43 | 37 | 33 | 44 | 27 | 38 | 37 | 56 | 48 | 53 |
| hsa-miR-4516 | MIMAT0019053 | miRNA | 100 | 8 | 26 | 31 | 10 | 15 | 14 | 18 | 19 | 20 | 20 | 14 | 22 |
| hsa-miR-451a | MIMAT0001631 | miRNA | 100 | 7 | 23842 | 5576 | 1020 | 2493 | 650 | 6029 | 1125 | 811 | 2351 | 581 | 446 |
| hsa-miR-452-5p | MIMAT0001635 | miRNA | 83 | 3 | 5 | 3 | 3 | 5 | 1 | 1 | 5 | 3 | 3 | 2 | 3 |
| hsa-miR-4521 | MIMAT0019058 | miRNA | 100 | 7 | 23 | 12 | 9 | 7 | 14 | 8 | 11 | 11 | 6 | 7 | 10 |
| hsa-miR-4524a-5p | MIMAT0019062 | miRNA | 92 | 2 | 6 | 9 | 3 | 2 | 3 | 3 | 7 | 3 | 4 | 1 | 2 |
| hsa-miR-4531 | MIMAT0019070 | miRNA | 100 | 10 | 4 | 16 | 3 | 6 | 10 | 8 | 6 | 10 | 7 | 9 | 17 |
| hsa-miR-4532 | MIMAT0019071 | miRNA | 100 | 2 | 3 | 7 | 5 | 7 | 9 | 5 | 10 | 4 | 5 | 3 | 4 |
| hsa-miR-4536-3p | MIMAT0020959 | miRNA | 100 | 16 | 15 | 24 | 19 | 17 | 17 | 21 | 15 | 18 | 28 | 24 | 15 |
| hsa-miR-4536-5p | MIMAT0019078 | miRNA | 100 | 9 | 8 | 15 | 15 | 8 | 13 | 6 | 8 | 9 | 7 | 8 | 14 |
| hsa-miR-454-3p | MIMAT0003885 | miRNA | 100 | 14 | 13 | 19 | 11 | 14 | 18 | 6 | 18 | 25 | 26 | 16 | 23 |
| hsa-miR-455-3p | MIMAT0004784 | miRNA | 100 | 8 | 8 | 12 | 11 | 11 | 6 | 8 | 6 | 7 | 4 | 4 | 8 |
| hsa-miR-455-5p | MIMAT0003150 | miRNA | 100 | 30 | 67 | 20 | 16 | 28 | 37 | 19 | 23 | 32 | 27 | 35 | 39 |
| hsa-miR-4647 | MIMAT0019709 | miRNA | 100 | 6 | 22 | 26 | 15 | 9 | 12 | 15 | 19 | 15 | 24 | 13 | 19 |
| hsa-miR-4707-3p | MIMAT0019808 | miRNA | 100 | 8 | 11 | 9 | 5 | 9 | 10 | 11 | 12 | 8 | 9 | 8 | 7 |
| hsa-miR-4707-5p | MIMAT0019807 | miRNA | 100 | 23 | 14 | 21 | 26 | 20 | 12 | 23 | 14 | 14 | 18 | 19 | 22 |
| hsa-miR-4741 | MIMAT0019871 | miRNA | 100 | 10 | 10 | 11 | 7 | 9 | 6 | 7 | 9 | 8 | 7 | 8 | 12 |
| hsa-miR-4755-5p | MIMAT0019895 | miRNA | 100 | 7 | 15 | 14 | 11 | 9 | 12 | 10 | 5 | 13 | 8 | 7 | 6 |
| hsa-miR-4787-3p | MIMAT0019957 | miRNA | 92 | 4 | 1 | 5 | 2 | 6 | 7 | 7 | 4 | 4 | 6 | 5 | 8 |
| hsa-miR-4787-5p | MIMAT0019956 | miRNA | 100 | 8 | 11 | 12 | 8 | 3 | 15 | 7 | 11 | 11 | 15 | 5 | 14 |
| hsa-miR-4792 | MIMAT0019964 | miRNA | 100 | 6 | 13 | 15 | 14 | 9 | 10 | 11 | 12 | 10 | 12 | 10 | 16 |
| hsa-miR-483-3p | MIMAT0002173 | miRNA | 100 | 11 | 11 | 12 | 23 | 15 | 9 | 4 | 9 | 15 | 13 | 9 | 11 |
| hsa-miR-483-5p | MIMAT0004761 | miRNA | 100 | 2 | 3 | 6 | 7 | 2 | 3 | 2 | 4 | 2 | 6 | 2 | 5 |
| hsa-miR-484 | MIMAT0002174 | miRNA | 100 | 7 | 13 | 12 | 5 | 4 | 20 | 9 | 6 | 12 | 8 | 11 | 17 |
| hsa-miR-485-3p | MIMAT0002176 | miRNA | 100 | 68 | 32 | 15 | 15 | 11 | 15 | 13 | 9 | 15 | 13 | 7 | 11 |
| hsa-miR-485-5p | MIMAT0002175 | miRNA | 92 | 2 | 9 | 8 | 6 | 4 | 5 | 3 | 5 | 5 | 3 | 1 | 11 |
| hsa-miR-486-3p | MIMAT0004762 | miRNA | 100 | 3 | 7 | 4 | 4 | 6 | 7 | 6 | 5 | 2 | 6 | 6 | 10 |
| hsa-miR-487a-3p | MIMAT0002178 | miRNA | 100 | 35 | 22 | 17 | 20 | 20 | 15 | 15 | 20 | 22 | 12 | 14 | 15 |

| | | | | | | | | | | | | | | | |
|---|---|---|---|---|---|---|---|---|---|---|---|---|---|---|---|
| hsa-miR-487b-3p | MIMAT0003180 | miRNA | 100 | 56 | 56 | 12 | 33 | 14 | 22 | 19 | 47 | 23 | 14 | 18 | 26 |
| hsa-miR-487b-5p | MIMAT0026614 | miRNA | 100 | 16 | 19 | 22 | 13 | 27 | 19 | 17 | 22 | 14 | 21 | 8 | 16 |
| hsa-miR-488-3p | MIMAT0004763 | miRNA | 100 | 3 | 4 | 13 | 5 | 9 | 4 | 3 | 6 | 4 | 8 | 3 | 7 |
| hsa-miR-489-3p | MIMAT0002805 | miRNA | 100 | 6 | 7 | 12 | 4 | 5 | 8 | 3 | 5 | 8 | 7 | 8 | 9 |
| hsa-miR-490-3p | MIMAT0002806 | miRNA | 100 | 8 | 10 | 11 | 9 | 10 | 4 | 6 | 9 | 6 | 12 | 8 | 5 |
| hsa-miR-490-5p | MIMAT0004764 | miRNA | 92 | 4 | 3 | 4 | 3 | 1 | 10 | 9 | 3 | 4 | 5 | 6 | 2 |
| hsa-miR-491-3p | MIMAT0004765 | miRNA | 100 | 4 | 9 | 8 | 4 | 2 | 2 | 5 | 4 | 9 | 7 | 8 | 6 |
| hsa-miR-491-5p | MIMAT0002807 | miRNA | 100 | 2 | 23 | 16 | 7 | 10 | 11 | 9 | 13 | 6 | 6 | 7 | 9 |
| hsa-miR-492 | MIMAT0002812 | miRNA | 100 | 4 | 7 | 7 | 4 | 3 | 4 | 6 | 4 | 9 | 7 | 4 | 6 |
| hsa-miR-493-3p | MIMAT0003161 | miRNA | 100 | 22 | 49 | 17 | 22 | 9 | 22 | 10 | 11 | 19 | 12 | 6 | 23 |
| hsa-miR-494-3p | MIMAT0002816 | miRNA | 100 | 6 | 12 | 5 | 8 | 6 | 12 | 4 | 5 | 20 | 8 | 6 | 16 |
| hsa-miR-494-5p | MIMAT0026607 | miRNA | 92 | 1 | 7 | 11 | 4 | 8 | 2 | 7 | 5 | 8 | 7 | 6 | 3 |
| hsa-miR-495-3p | MIMAT0002817 | miRNA | 100 | 192 | 298 | 22 | 97 | 25 | 25 | 32 | 100 | 39 | 32 | 33 | 33 |
| hsa-miR-495-5p | MIMAT0022924 | miRNA | 100 | 41 | 38 | 45 | 35 | 25 | 27 | 25 | 39 | 19 | 38 | 30 | 31 |
| hsa-miR-496 | MIMAT0002818 | miRNA | 100 | 18 | 8 | 16 | 21 | 3 | 11 | 2 | 7 | 12 | 5 | 6 | 14 |
| hsa-miR-497-5p | MIMAT0002820 | miRNA | 100 | 6 | 183 | 31 | 56 | 24 | 80 | 30 | 34 | 81 | 57 | 31 | 62 |
| hsa-miR-498 | MIMAT0002824 | miRNA | 100 | 5 | 11 | 12 | 11 | 12 | 12 | 14 | 17 | 10 | 11 | 14 | 11 |
| hsa-miR-499a-3p | MIMAT0004772 | miRNA | 100 | 5 | 3 | 13 | 8 | 4 | 12 | 5 | 11 | 10 | 4 | 6 | 7 |
| hsa-miR-499a-5p | MIMAT0002870 | miRNA | 100 | 10 | 15 | 17 | 25 | 13 | 7 | 12 | 21 | 17 | 16 | 7 | 11 |
| hsa-miR-499b-3p | MIMAT0019898 | miRNA | 100 | 4 | 2 | 10 | 13 | 6 | 12 | 7 | 5 | 2 | 2 | 6 | 7 |
| hsa-miR-499b-5p | MIMAT0019897 | miRNA | 100 | 9 | 10 | 13 | 5 | 18 | 12 | 4 | 8 | 5 | 9 | 7 | 9 |
| hsa-miR-5001-3p | MIMAT0021022 | miRNA | 83 | 1 | 8 | 5 | 5 | 9 | 4 | 4 | 1 | 7 | 2 | 3 | 3 |
| hsa-miR-5001-5p | MIMAT0021021 | miRNA | 100 | 4 | 11 | 19 | 8 | 12 | 11 | 18 | 17 | 18 | 11 | 13 | 13 |
| hsa-miR-500a-5p & miR-501-5p | MIMAT0004773 | miRNA | 100 | 4 | 40 | 14 | 25 | 11 | 32 | 10 | 16 | 28 | 17 | 12 | 13 |
| hsa-miR-501-3p | MIMAT0004774 | miRNA | 100 | 7 | 6 | 3 | 6 | 4 | 8 | 4 | 6 | 7 | 4 | 8 | 2 |
| hsa-miR-5010-3p | MIMAT0021044 | miRNA | 100 | 13 | 19 | 34 | 18 | 34 | 15 | 17 | 19 | 16 | 19 | 14 | 21 |
| hsa-miR-5010-5p | MIMAT0021043 | miRNA | 83 | 1 | 10 | 4 | 3 | 2 | 5 | 1 | 4 | 3 | 5 | 3 | 3 |
| hsa-miR-502-3p | MIMAT0004775 | miRNA | 100 | 5 | 15 | 10 | 5 | 13 | 11 | 6 | 7 | 10 | 8 | 10 | 8 |
| hsa-miR-502-5p | MIMAT0002873 | miRNA | 100 | 16 | 11 | 16 | 15 | 19 | 10 | 14 | 18 | 8 | 16 | 17 | 13 |
| hsa-miR-503-3p | MIMAT0022925 | miRNA | 100 | 8 | 6 | 13 | 11 | 11 | 7 | 9 | 15 | 7 | 8 | 6 | 11 |
| hsa-miR-503-5p | MIMAT0002874 | miRNA | 100 | 145 | 42 | 19 | 28 | 18 | 34 | 11 | 11 | 16 | 22 | 16 | 34 |
| hsa-miR-504-3p | MIMAT0026612 | miRNA | 92 | 3 | 1 | 6 | 3 | 4 | 5 | 2 | 3 | 2 | 6 | 6 | 5 |
| hsa-miR-504-5p | MIMAT0002875 | miRNA | 100 | 5 | 5 | 6 | 3 | 6 | 13 | 8 | 9 | 13 | 7 | 7 | 6 |
| hsa-miR-505-3p | MIMAT0002876 | miRNA | 100 | 28 | 38 | 10 | 11 | 10 | 25 | 4 | 8 | 21 | 9 | 13 | 12 |
| hsa-miR-506-3p | MIMAT0002878 | miRNA | 100 | 5 | 23 | 8 | 9 | 10 | 7 | 8 | 7 | 5 | 11 | 4 | 8 |
| hsa-miR-506-5p | MIMAT0022701 | miRNA | 100 | 8 | 13 | 10 | 9 | 11 | 16 | 8 | 6 | 7 | 15 | 5 | 10 |
| hsa-miR-507 | MIMAT0002879 | miRNA | 92 | 1 | 7 | 4 | 3 | 8 | 5 | 5 | 9 | 8 | 9 | 4 | 4 |
| hsa-miR-508-3p | MIMAT0002880 | miRNA | 92 | 2 | 13 | 6 | 1 | 5 | 9 | 5 | 3 | 8 | 13 | 5 | 8 |
| hsa-miR-508-5p | MIMAT0004778 | miRNA | 100 | 13 | 16 | 19 | 10 | 18 | 21 | 19 | 15 | 17 | 11 | 11 | 18 |
| hsa-miR-509-3-5p | MIMAT0004975 | miRNA | 100 | 4 | 12 | 12 | 5 | 8 | 7 | 7 | 18 | 10 | 5 | 11 | 10 |
| hsa-miR-509-3p | MIMAT0002881 | miRNA | 100 | 2 | 18 | 10 | 11 | 13 | 14 | 11 | 7 | 14 | 11 | 11 | 7 |
| hsa-miR-509-5p | MIMAT0004779 | miRNA | 92 | 6 | 17 | 9 | 3 | 16 | 5 | 8 | 1 | 7 | 7 | 3 | 6 |
| hsa-miR-510-3p | MIMAT0026613 | miRNA | 100 | 9 | 13 | 22 | 15 | 20 | 11 | 13 | 25 | 16 | 14 | 14 | 18 |
| hsa-miR-510-5p | MIMAT0002882 | miRNA | 92 | 1 | 4 | 6 | 2 | 3 | 4 | 3 | 4 | 6 | 4 | 3 | 8 |
| hsa-miR-511-5p | MIMAT0002808 | miRNA | 100 | 6 | 14 | 9 | 11 | 5 | 8 | 10 | 14 | 8 | 6 | 3 | 11 |

| | | | | | | | | | | | | | | | |
|---|---|---|---|---|---|---|---|---|---|---|---|---|---|---|---|
| hsa-miR-512-3p | MIMAT0002823 | miRNA | 92 | 1 | 5 | 8 | 4 | 8 | 5 | 2 | 3 | 3 | 7 | 3 | 4 |
| hsa-miR-512-5p | MIMAT0002822 | miRNA | 100 | 11 | 21 | 26 | 19 | 24 | 15 | 21 | 18 | 15 | 22 | 22 | 27 |
| hsa-miR-513a-3p | MIMAT0004777 | miRNA | 100 | 14 | 21 | 22 | 17 | 27 | 22 | 31 | 26 | 15 | 35 | 26 | 24 |
| hsa-miR-513a-5p | MIMAT0002877 | miRNA | 100 | 9 | 11 | 21 | 19 | 12 | 22 | 23 | 23 | 17 | 17 | 19 | 23 |
| hsa-miR-513b-5p | MIMAT0005788 | miRNA | 100 | 5 | 15 | 10 | 8 | 11 | 9 | 11 | 9 | 7 | 7 | 10 | 7 |
| hsa-miR-513c-3p | MIMAT0022728 | miRNA | 100 | 5 | 10 | 11 | 6 | 4 | 6 | 14 | 12 | 6 | 3 | 7 | 8 |
| hsa-miR-513c-5p | MIMAT0005789 | miRNA | 100 | 5 | 17 | 12 | 9 | 10 | 16 | 11 | 10 | 10 | 11 | 14 | 12 |
| hsa-miR-514a-3p | MIMAT0002883 | miRNA | 100 | 9 | 20 | 23 | 15 | 30 | 24 | 21 | 20 | 12 | 22 | 21 | 21 |
| hsa-miR-514a-5p | MIMAT0022702 | miRNA | 100 | 3 | 17 | 5 | 8 | 14 | 3 | 10 | 5 | 4 | 2 | 9 | 3 |
| hsa-miR-514b-3p | MIMAT0015088 | miRNA | 100 | 5 | 4 | 5 | 5 | 4 | 7 | 5 | 9 | 2 | 6 | 8 | 7 |
| hsa-miR-514b-5p | MIMAT0015087 | miRNA | 100 | 19 | 41 | 27 | 34 | 35 | 37 | 33 | 29 | 19 | 40 | 22 | 25 |
| hsa-miR-515-3p | MIMAT0002827 | miRNA | 100 | 3 | 11 | 4 | 6 | 5 | 5 | 8 | 9 | 10 | 6 | 5 | 12 |
| hsa-miR-515-5p | MIMAT0002826 | miRNA | 92 | 4 | 4 | 10 | 3 | 9 | 2 | 7 | 3 | 8 | 4 | 1 | 4 |
| hsa-miR-516a-3p & miR-516b-3p | MIMAT0006778 | miRNA | 92 | 5 | 5 | 10 | 1 | 6 | 7 | 6 | 3 | 3 | 4 | 3 | 5 |
| hsa-miR-516a-5p | MIMAT0004770 | miRNA | 100 | 6 | 5 | 8 | 4 | 8 | 6 | 4 | 6 | 2 | 3 | 6 | 3 |
| hsa-miR-516b-5p | MIMAT0002859 | miRNA | 100 | 13 | 12 | 12 | 13 | 13 | 8 | 12 | 16 | 5 | 17 | 16 | 10 |
| hsa-miR-517a-3p | MIMAT0002852 | miRNA | 92 | 5 | 7 | 10 | 1 | 8 | 10 | 9 | 9 | 11 | 12 | 6 | 10 |
| hsa-miR-517b-3p | MIMAT0002857 | miRNA | 92 | 4 | 3 | 4 | 2 | 9 | 1 | 2 | 2 | 4 | 2 | 3 | 3 |
| hsa-miR-517c-3p & miR-519a-3p | MIMAT0002866 | miRNA | 100 | 8 | 14 | 26 | 15 | 24 | 15 | 21 | 20 | 16 | 17 | 17 | 17 |
| hsa-miR-518b | MIMAT0002844 | miRNA | 100 | 3 | 81 | 23 | 21 | 37 | 66 | 23 | 15 | 72 | 27 | 42 | 54 |
| hsa-miR-518c-3p | MIMAT0002848 | miRNA | 100 | 6 | 13 | 3 | 6 | 4 | 10 | 7 | 7 | 9 | 3 | 4 | 7 |
| hsa-miR-518d-3p | MIMAT0002864 | miRNA | 100 | 2 | 5 | 2 | 6 | 3 | 10 | 4 | 3 | 2 | 3 | 3 | 4 |
| hsa-miR-518e-3p | MIMAT0002861 | miRNA | 100 | 5 | 7 | 15 | 4 | 9 | 7 | 12 | 11 | 3 | 14 | 5 | 4 |
| hsa-miR-518f-3p | MIMAT0002842 | miRNA | 100 | 10 | 8 | 12 | 11 | 8 | 16 | 11 | 16 | 11 | 12 | 18 | 16 |
| hsa-miR-5196-3p & miR-6732-3p | MIMAT0021129 | miRNA | 100 | 19 | 7 | 9 | 9 | 9 | 11 | 10 | 11 | 6 | 8 | 6 | 15 |
| hsa-miR-5196-5p | MIMAT0021128 | miRNA | 100 | 4 | 15 | 12 | 11 | 8 | 5 | 9 | 8 | 10 | 12 | 8 | 9 |
| hsa-miR-519b-3p | MIMAT0002837 | miRNA | 100 | 7 | 14 | 12 | 7 | 10 | 10 | 10 | 6 | 9 | 7 | 3 | 11 |
| hsa-miR-519b-5p & miR-519c-5p& miR-523-5p & miR-518 | MIMAT0005454 | miRNA | 100 | 4 | 6 | 5 | 5 | 3 | 2 | 4 | 6 | 4 | 6 | 6 | 3 |
| hsa-miR-519c-3p | MIMAT0002832 | miRNA | 100 | 15 | 11 | 25 | 17 | 22 | 20 | 23 | 20 | 17 | 21 | 18 | 13 |
| hsa-miR-519d-3p | MIMAT0002853 | miRNA | 100 | 6 | 8 | 11 | 4 | 11 | 8 | 9 | 8 | 7 | 7 | 9 | 11 |
| hsa-miR-519e-3p | MIMAT0002829 | miRNA | 100 | 4 | 4 | 3 | 3 | 10 | 7 | 4 | 6 | 8 | 3 | 4 | 12 |
| hsa-miR-520a-3p | MIMAT0002834 | miRNA | 83 | 1 | 1 | 2 | 6 | 2 | 5 | 8 | 5 | 3 | 3 | 3 | 2 |
| hsa-miR-520a-5p | MIMAT0002833 | miRNA | 100 | 7 | 5 | 9 | 6 | 9 | 11 | 6 | 8 | 6 | 7 | 9 | 3 |
| hsa-miR-520b | MIMAT0002843 | miRNA | 100 | 2 | 5 | 9 | 6 | 4 | 2 | 8 | 3 | 5 | 4 | 5 | 12 |
| hsa-miR-520c-3p | MIMAT0002846 | miRNA | 100 | 5 | 9 | 9 | 10 | 8 | 10 | 14 | 8 | 13 | 11 | 14 | 9 |
| hsa-miR-520d-3p | MIMAT0002856 | miRNA | 100 | 9 | 10 | 10 | 7 | 15 | 7 | 13 | 8 | 10 | 9 | 7 | 15 |
| hsa-miR-520d-5p & miR-527 & miR-518a-5p | MIMAT0002855 | miRNA | 92 | 3 | 6 | 5 | 7 | 2 | 10 | 1 | 3 | 8 | 6 | 3 | 2 |
| hsa-miR-520e | MIMAT0002825 | miRNA | 92 | 2 | 3 | 4 | 6 | 3 | 6 | 6 | 5 | 3 | 6 | 7 | 1 |
| hsa-miR-520f-3p | MIMAT0002830 | miRNA | 92 | 1 | 6 | 9 | 3 | 6 | 8 | 3 | 4 | 3 | 4 | 3 | 3 |
| hsa-miR-520g-3p | MIMAT0002858 | miRNA | 100 | 4 | 7 | 6 | 6 | 5 | 4 | 7 | 4 | 6 | 7 | 3 | 11 |
| hsa-miR-520h | MIMAT0002867 | miRNA | 100 | 3 | 14 | 10 | 8 | 7 | 12 | 9 | 13 | 6 | 11 | 3 | 12 |
| hsa-miR-521 | MIMAT0002854 | miRNA | 100 | 10 | 10 | 21 | 10 | 14 | 6 | 18 | 10 | 4 | 12 | 14 | 10 |
| hsa-miR-522-3p | MIMAT0002868 | miRNA | 100 | 12 | 17 | 17 | 17 | 22 | 10 | 26 | 20 | 8 | 18 | 24 | 22 |
| hsa-miR-523-3p | MIMAT0002840 | miRNA | 100 | 7 | 4 | 5 | 2 | 4 | 3 | 7 | 3 | 4 | 5 | 2 | 3 |
| hsa-miR-524-3p | MIMAT0002850 | miRNA | 100 | 2 | 9 | 9 | 6 | 7 | 8 | 4 | 5 | 3 | 8 | 3 | 6 |

| | | | | | | | | | | | | | | | |
|---|---|---|---|---|---|---|---|---|---|---|---|---|---|---|---|
| hsa-miR-525-3p | MIMAT0002839 | miRNA | 83 | 1 | 7 | 9 | 3 | 10 | 8 | 9 | 5 | 5 | 6 | 2 | 1 |
| hsa-miR-525-5p | MIMAT0002838 | miRNA | 100 | 11 | 25 | 20 | 19 | 22 | 21 | 33 | 16 | 26 | 30 | 17 | 14 |
| hsa-miR-526a & miR-518c-5p & miR-518d-5p | MIMAT0002845 | miRNA | 100 | 4 | 8 | 8 | 9 | 15 | 11 | 9 | 11 | 6 | 10 | 7 | 11 |
| hsa-miR-526b-5p | MIMAT0002835 | miRNA | 100 | 6 | 17 | 17 | 18 | 22 | 11 | 29 | 20 | 10 | 31 | 22 | 15 |
| hsa-miR-532-3p | MIMAT0004780 | miRNA | 100 | 13 | 31 | 17 | 18 | 27 | 17 | 25 | 18 | 12 | 17 | 19 | 12 |
| hsa-miR-532-5p | MIMAT0002888 | miRNA | 100 | 3 | 9 | 6 | 9 | 2 | 3 | 4 | 9 | 6 | 8 | 2 | 7 |
| hsa-miR-539-3p | MIMAT0022705 | miRNA | 100 | 5 | 7 | 10 | 5 | 7 | 5 | 5 | 5 | 10 | 10 | 5 | 4 |
| hsa-miR-539-5p | MIMAT0003163 | miRNA | 100 | 5 | 39 | 17 | 14 | 9 | 16 | 6 | 12 | 13 | 11 | 8 | 17 |
| hsa-miR-541-3p | MIMAT0004920 | miRNA | 100 | 4 | 3 | 8 | 5 | 3 | 7 | 4 | 7 | 7 | 4 | 3 | 9 |
| hsa-miR-542-3p | MIMAT0003389 | miRNA | 100 | 18 | 29 | 35 | 15 | 22 | 20 | 23 | 15 | 27 | 24 | 22 | 12 |
| hsa-miR-542-5p | MIMAT0003340 | miRNA | 100 | 11 | 25 | 6 | 15 | 8 | 17 | 14 | 17 | 16 | 13 | 13 | 13 |
| hsa-miR-543 | MIMAT0004954 | miRNA | 100 | 162 | 104 | 30 | 95 | 29 | 30 | 40 | 53 | 44 | 32 | 28 | 34 |
| hsa-miR-544a | MIMAT0003164 | miRNA | 100 | 6 | 8 | 10 | 5 | 6 | 9 | 10 | 11 | 10 | 8 | 4 | 10 |
| hsa-miR-545-3p | MIMAT0003165 | miRNA | 100 | 2 | 14 | 3 | 3 | 3 | 5 | 2 | 4 | 13 | 8 | 5 | 7 |
| hsa-miR-548a-3p | MIMAT0003251 | miRNA | 83 | 7 | 6 | 7 | 4 | 1 | 2 | 5 | 5 | 2 | 2 | 1 | 6 |
| hsa-miR-548a-5p | MIMAT0004803 | miRNA | 100 | 16 | 14 | 25 | 28 | 18 | 10 | 29 | 24 | 23 | 24 | 23 | 25 |
| hsa-miR-548aa & miR-548t-3p | MIMAT0018447 | miRNA | 100 | 4 | 3 | 5 | 7 | 5 | 9 | 6 | 3 | 7 | 4 | 6 | 11 |
| hsa-miR-548ad-3p | MIMAT0018946 | miRNA | 92 | 1 | 11 | 6 | 6 | 8 | 6 | 5 | 4 | 4 | 5 | 5 | 3 |
| hsa-miR-548ah-5p | MIMAT0018972 | miRNA | 100 | 10 | 18 | 16 | 14 | 15 | 19 | 13 | 11 | 13 | 12 | 11 | 11 |
| hsa-miR-548ai & miR-570-5p | MIMAT0018989 | miRNA | 83 | 1 | 4 | 3 | 3 | 3 | 4 | 4 | 1 | 5 | 2 | 8 | 13 |
| hsa-miR-548ak | MIMAT0019013 | miRNA | 100 | 8 | 12 | 17 | 11 | 17 | 14 | 7 | 7 | 6 | 12 | 7 | 15 |
| hsa-miR-548al | MIMAT0019024 | miRNA | 100 | 8 | 12 | 10 | 6 | 5 | 10 | 6 | 10 | 6 | 11 | 12 | 13 |
| hsa-miR-548ar-3p | MIMAT0022266 | miRNA | 100 | 16 | 17 | 22 | 22 | 15 | 19 | 21 | 20 | 12 | 25 | 21 | 21 |
| hsa-miR-548ar-5p | MIMAT0022265 | miRNA | 100 | 21 | 17 | 18 | 17 | 15 | 25 | 14 | 17 | 24 | 17 | 8 | 27 |
| hsa-miR-548b-3p | MIMAT0003254 | miRNA | 100 | 4 | 5 | 6 | 6 | 7 | 8 | 2 | 2 | 6 | 3 | 5 | 4 |
| hsa-miR-548c-5p & miR-548o-5p & miR-548am-5p | MIMAT0004806 | miRNA | 100 | 2 | 3 | 8 | 5 | 2 | 9 | 3 | 3 | 7 | 5 | 2 | 4 |
| hsa-miR-548d-3p | MIMAT0003323 | miRNA | 100 | 2 | 6 | 11 | 7 | 5 | 7 | 7 | 5 | 7 | 2 | 10 | 8 |
| hsa-miR-548d-5p | MIMAT0004812 | miRNA | 100 | 10 | 17 | 10 | 5 | 8 | 9 | 3 | 9 | 13 | 10 | 8 | 14 |
| hsa-miR-548e-3p | MIMAT0005874 | miRNA | 92 | 6 | 7 | 5 | 4 | 8 | 6 | 6 | 2 | 6 | 5 | 1 | 6 |
| hsa-miR-548e-5p | MIMAT0026736 | miRNA | 100 | 16 | 34 | 27 | 28 | 37 | 29 | 36 | 39 | 23 | 42 | 36 | 26 |
| hsa-miR-548g-3p | MIMAT0005912 | miRNA | 100 | 15 | 25 | 15 | 12 | 27 | 17 | 21 | 13 | 15 | 23 | 21 | 34 |
| hsa-miR-548h-5p | MIMAT0005928 | miRNA | 100 | 2 | 6 | 12 | 2 | 10 | 8 | 10 | 3 | 4 | 7 | 6 | 5 |
| hsa-miR-548i | MIMAT0005935 | miRNA | 100 | 6 | 14 | 9 | 5 | 14 | 11 | 5 | 10 | 9 | 2 | 9 | 2 |
| hsa-miR-548j-3p | MIMAT0026737 | miRNA | 100 | 8 | 11 | 13 | 8 | 8 | 6 | 5 | 8 | 7 | 7 | 5 | 8 |
| hsa-miR-548j-5p | MIMAT0005875 | miRNA | 100 | 3 | 6 | 12 | 7 | 5 | 7 | 11 | 7 | 6 | 11 | 7 | 10 |
| hsa-miR-548k | MIMAT0005882 | miRNA | 100 | 12 | 15 | 18 | 10 | 17 | 16 | 15 | 12 | 11 | 14 | 10 | 18 |
| hsa-miR-548l | MIMAT0005889 | miRNA | 100 | 5 | 9 | 9 | 3 | 8 | 7 | 8 | 5 | 5 | 6 | 2 | 8 |
| hsa-miR-548m | MIMAT0005917 | miRNA | 100 | 5 | 24 | 20 | 15 | 26 | 18 | 20 | 21 | 18 | 15 | 13 | 18 |
| hsa-miR-548n | MIMAT0005916 | miRNA | 92 | 1 | 8 | 9 | 7 | 6 | 6 | 7 | 10 | 9 | 6 | 9 | 9 |
| hsa-miR-548o-3p & miR-548ah-3p & miR-548av-3p | MIMAT0005919 | miRNA | 92 | 3 | 7 | 12 | 14 | 5 | 8 | 9 | 7 | 9 | 9 | 1 | 7 |
| hsa-miR-548q | MIMAT0011163 | miRNA | 100 | 8 | 5 | 4 | 7 | 11 | 4 | 5 | 5 | 8 | 7 | 4 | 10 |
| hsa-miR-548v | MIMAT0015020 | miRNA | 100 | 13 | 15 | 18 | 18 | 20 | 16 | 14 | 25 | 15 | 21 | 22 | 20 |
| hsa-miR-548y | MIMAT0018354 | miRNA | 100 | 15 | 29 | 32 | 12 | 22 | 19 | 19 | 24 | 13 | 14 | 23 | 19 |
| hsa-miR-548z & miR-548h-3p | MIMAT0018446 | miRNA | 92 | 1 | 7 | 9 | 6 | 4 | 10 | 14 | 7 | 9 | 10 | 2 | 9 |
| hsa-miR-549a | MIMAT0003333 | miRNA | 92 | 1 | 8 | 9 | 7 | 2 | 7 | 10 | 8 | 5 | 3 | 3 | 6 |

| | | | | | | | | | | | | | | | |
|---|---|---|---|---|---|---|---|---|---|---|---|---|---|---|---|
| hsa-miR-550a-5p | MIMAT0004800 | miRNA | 75 | 2 | 3 | 3 | 3 | 1 | 3 | 4 | 1 | 3 | 2 | 1 | 3 |
| hsa-miR-551a | MIMAT0003214 | miRNA | 100 | 3 | 12 | 13 | 7 | 8 | 12 | 7 | 6 | 15 | 9 | 2 | 2 |
| hsa-miR-551b-3p | MIMAT0003233 | miRNA | 100 | 11 | 23 | 16 | 15 | 18 | 19 | 21 | 18 | 16 | 22 | 20 | 22 |
| hsa-miR-552-3p | MIMAT0003215 | miRNA | 100 | 6 | 13 | 10 | 11 | 9 | 10 | 7 | 9 | 6 | 8 | 9 | 7 |
| hsa-miR-553 | MIMAT0003216 | miRNA | 92 | 7 | 6 | 8 | 1 | 3 | 4 | 5 | 9 | 7 | 5 | 3 | 3 |
| hsa-miR-554 | MIMAT0003217 | miRNA | 92 | 5 | 7 | 9 | 2 | 5 | 6 | 1 | 3 | 4 | 4 | 4 | 4 |
| hsa-miR-555 | MIMAT0003219 | miRNA | 100 | 6 | 4 | 4 | 5 | 5 | 10 | 2 | 4 | 4 | 5 | 6 | 4 |
| hsa-miR-556-3p | MIMAT0004793 | miRNA | 100 | 5 | 12 | 7 | 4 | 6 | 3 | 5 | 10 | 5 | 6 | 6 | 5 |
| hsa-miR-556-5p | MIMAT0003220 | miRNA | 100 | 12 | 24 | 18 | 11 | 28 | 14 | 21 | 20 | 16 | 16 | 11 | 16 |
| hsa-miR-561-3p | MIMAT0003225 | miRNA | 83 | 2 | 5 | 2 | 3 | 3 | 8 | 6 | 1 | 4 | 2 | 1 | 5 |
| hsa-miR-561-5p | MIMAT0022706 | miRNA | 100 | 7 | 17 | 13 | 12 | 7 | 12 | 10 | 15 | 13 | 7 | 9 | 13 |
| hsa-miR-562 | MIMAT0003226 | miRNA | 100 | 3 | 6 | 12 | 2 | 9 | 6 | 8 | 11 | 7 | 10 | 6 | 4 |
| hsa-miR-563 | MIMAT0003227 | miRNA | 100 | 9 | 13 | 21 | 10 | 16 | 17 | 23 | 21 | 12 | 19 | 19 | 12 |
| hsa-miR-564 | MIMAT0003228 | miRNA | 100 | 2 | 6 | 6 | 10 | 7 | 12 | 8 | 4 | 4 | 3 | 2 | 7 |
| hsa-miR-566 | MIMAT0003230 | miRNA | 92 | 1 | 8 | 7 | 7 | 7 | 6 | 10 | 6 | 9 | 10 | 14 | 10 |
| hsa-miR-567 | MIMAT0003231 | miRNA | 92 | 2 | 9 | 8 | 4 | 7 | 6 | 4 | 6 | 3 | 4 | 5 | 1 |
| hsa-miR-568 | MIMAT0003232 | miRNA | 100 | 5 | 7 | 6 | 3 | 4 | 11 | 10 | 2 | 3 | 4 | 6 | 8 |
| hsa-miR-570-3p | MIMAT0003235 | miRNA | 100 | 7 | 12 | 14 | 11 | 10 | 6 | 4 | 12 | 10 | 10 | 9 | 6 |
| hsa-miR-571 | MIMAT0003236 | miRNA | 100 | 3 | 6 | 8 | 3 | 5 | 7 | 4 | 7 | 9 | 5 | 3 | 5 |
| hsa-miR-572 | MIMAT0003237 | miRNA | 100 | 3 | 18 | 15 | 11 | 15 | 12 | 13 | 12 | 7 | 17 | 10 | 15 |
| hsa-miR-573 | MIMAT0003238 | miRNA | 100 | 6 | 3 | 12 | 5 | 6 | 10 | 5 | 6 | 7 | 6 | 6 | 9 |
| hsa-miR-574-3p | MIMAT0003239 | miRNA | 100 | 8 | 24 | 15 | 16 | 9 | 12 | 8 | 17 | 12 | 19 | 14 | 17 |
| hsa-miR-574-5p | MIMAT0004795 | miRNA | 100 | 134 | 277 | 44 | 120 | 41 | 122 | 18 | 25 | 91 | 14 | 18 | 53 |
| hsa-miR-575 | MIMAT0003240 | miRNA | 100 | 23 | 44 | 27 | 17 | 21 | 39 | 21 | 15 | 31 | 13 | 12 | 35 |
| hsa-miR-576-3p | MIMAT0004796 | miRNA | 100 | 9 | 6 | 23 | 20 | 31 | 14 | 20 | 19 | 19 | 24 | 24 | 17 |
| hsa-miR-576-5p | MIMAT0003241 | miRNA | 100 | 4 | 15 | 11 | 6 | 7 | 11 | 13 | 13 | 8 | 10 | 2 | 11 |
| hsa-miR-577 | MIMAT0003242 | miRNA | 100 | 10 | 17 | 9 | 6 | 14 | 8 | 12 | 9 | 7 | 8 | 8 | 10 |
| hsa-miR-578 | MIMAT0003243 | miRNA | 100 | 5 | 5 | 8 | 5 | 5 | 4 | 3 | 6 | 9 | 4 | 4 | 3 |
| hsa-miR-579-3p | MIMAT0003244 | miRNA | 0 | 1 | 1 | 1 | 1 | 1 | 1 | 1 | 1 | 1 | 1 | 1 | 1 |
| hsa-miR-579-5p | MIMAT0026616 | miRNA | 100 | 6 | 9 | 20 | 9 | 14 | 9 | 8 | 10 | 6 | 13 | 10 | 9 |
| hsa-miR-580-3p | MIMAT0003245 | miRNA | 92 | 7 | 7 | 8 | 8 | 12 | 1 | 9 | 9 | 4 | 4 | 7 | 9 |
| hsa-miR-582-3p | MIMAT0004797 | miRNA | 100 | 7 | 12 | 17 | 8 | 21 | 8 | 17 | 14 | 19 | 16 | 12 | 15 |
| hsa-miR-582-5p | MIMAT0003247 | miRNA | 100 | 23 | 265 | 46 | 42 | 62 | 60 | 52 | 36 | 56 | 53 | 59 | 91 |
| hsa-miR-584-3p | MIMAT0022708 | miRNA | 100 | 13 | 16 | 27 | 24 | 29 | 21 | 24 | 27 | 15 | 32 | 29 | 18 |
| hsa-miR-584-5p | MIMAT0003249 | miRNA | 100 | 10 | 16 | 19 | 13 | 13 | 11 | 18 | 11 | 16 | 23 | 11 | 12 |
| hsa-miR-585-3p | MIMAT0003250 | miRNA | 100 | 19 | 32 | 29 | 23 | 26 | 34 | 31 | 42 | 31 | 38 | 22 | 37 |
| hsa-miR-587 | MIMAT0003253 | miRNA | 100 | 8 | 12 | 13 | 12 | 6 | 15 | 15 | 10 | 22 | 7 | 7 | 11 |
| hsa-miR-589-5p | MIMAT0004799 | miRNA | 100 | 6 | 4 | 8 | 7 | 6 | 12 | 4 | 9 | 6 | 2 | 7 | 7 |
| hsa-miR-590-3p | MIMAT0004801 | miRNA | 100 | 5 | 9 | 8 | 5 | 5 | 8 | 5 | 10 | 6 | 8 | 7 | 8 |
| hsa-miR-590-5p | MIMAT0003258 | miRNA | 100 | 21 | 22 | 18 | 7 | 13 | 12 | 8 | 17 | 8 | 18 | 14 | 22 |
| hsa-miR-591 | MIMAT0003259 | miRNA | 100 | 5 | 7 | 15 | 7 | 8 | 7 | 13 | 6 | 3 | 12 | 6 | 7 |
| hsa-miR-592 | MIMAT0003260 | miRNA | 100 | 5 | 17 | 12 | 10 | 14 | 11 | 11 | 8 | 9 | 11 | 9 | 10 |
| hsa-miR-593-3p | MIMAT0004802 | miRNA | 100 | 19 | 3 | 16 | 7 | 7 | 12 | 8 | 6 | 7 | 9 | 7 | 6 |
| hsa-miR-595 | MIMAT0003263 | miRNA | 100 | 2 | 3 | 8 | 7 | 7 | 3 | 2 | 2 | 13 | 7 | 3 | 6 |
| hsa-miR-596 | MIMAT0003264 | miRNA | 100 | 2 | 2 | 9 | 5 | 6 | 9 | 5 | 3 | 10 | 3 | 5 | 6 |

| | | | | | | | | | | | | | | | |
|---|---|---|---|---|---|---|---|---|---|---|---|---|---|---|---|
| hsa-miR-597-5p | MIMAT0003265 | miRNA | 100 | 6 | 27 | 30 | 18 | 14 | 17 | 14 | 21 | 17 | 12 | 13 | 22 |
| hsa-miR-598-3p | MIMAT0003266 | miRNA | 100 | 31 | 50 | 24 | 23 | 28 | 30 | 30 | 31 | 34 | 28 | 23 | 32 |
| hsa-miR-599 | MIMAT0003267 | miRNA | 100 | 3 | 6 | 5 | 3 | 7 | 6 | 2 | 6 | 4 | 4 | 2 | 6 |
| hsa-miR-600 | MIMAT0003268 | miRNA | 100 | 5 | 2 | 4 | 3 | 5 | 3 | 2 | 5 | 4 | 3 | 2 | 4 |
| hsa-miR-601 | MIMAT0003269 | miRNA | 100 | 5 | 3 | 6 | 4 | 3 | 6 | 3 | 11 | 3 | 6 | 8 | 8 |
| hsa-miR-603 | MIMAT0003271 | miRNA | 100 | 12 | 8 | 14 | 6 | 11 | 8 | 12 | 12 | 12 | 7 | 11 | 10 |
| hsa-miR-604 | MIMAT0003272 | miRNA | 100 | 5 | 6 | 8 | 3 | 5 | 5 | 8 | 4 | 2 | 7 | 2 | 8 |
| hsa-miR-605-5p | MIMAT0003273 | miRNA | 100 | 6 | 10 | 13 | 11 | 14 | 15 | 14 | 11 | 12 | 11 | 5 | 15 |
| hsa-miR-606 | MIMAT0003274 | miRNA | 83 | 7 | 4 | 8 | 1 | 5 | 7 | 1 | 4 | 5 | 4 | 2 | 3 |
| hsa-miR-607 | MIMAT0003275 | miRNA | 100 | 20 | 27 | 31 | 18 | 25 | 20 | 29 | 27 | 19 | 29 | 19 | 22 |
| hsa-miR-608 | MIMAT0003276 | miRNA | 100 | 4 | 12 | 12 | 12 | 19 | 31 | 9 | 11 | 8 | 11 | 3 | 14 |
| hsa-miR-610 | MIMAT0003278 | miRNA | 100 | 4 | 19 | 11 | 7 | 9 | 8 | 5 | 4 | 7 | 7 | 5 | 6 |
| hsa-miR-612 | MIMAT0003280 | miRNA | 100 | 6 | 17 | 27 | 16 | 14 | 17 | 12 | 22 | 10 | 20 | 4 | 16 |
| hsa-miR-613 | MIMAT0003281 | miRNA | 100 | 5 | 12 | 10 | 5 | 4 | 12 | 8 | 8 | 4 | 6 | 3 | 11 |
| hsa-miR-614 | MIMAT0003282 | miRNA | 92 | 5 | 11 | 7 | 4 | 4 | 6 | 6 | 7 | 7 | 10 | 1 | 5 |
| hsa-miR-615-3p | MIMAT0003283 | miRNA | 100 | 14 | 17 | 36 | 15 | 21 | 14 | 24 | 29 | 18 | 16 | 15 | 25 |
| hsa-miR-615-5p | MIMAT0004804 | miRNA | 100 | 5 | 8 | 10 | 4 | 9 | 5 | 7 | 10 | 4 | 5 | 5 | 7 |
| hsa-miR-616-3p | MIMAT0004805 | miRNA | 100 | 13 | 18 | 24 | 14 | 19 | 27 | 11 | 21 | 29 | 17 | 12 | 16 |
| hsa-miR-617 | MIMAT0003286 | miRNA | 92 | 4 | 8 | 5 | 5 | 6 | 2 | 2 | 4 | 4 | 6 | 1 | 4 |
| hsa-miR-619-3p | MIMAT0003288 | miRNA | 100 | 5 | 9 | 5 | 2 | 7 | 5 | 4 | 2 | 3 | 4 | 4 | 7 |
| hsa-miR-620 | MIMAT0003289 | miRNA | 100 | 5 | 12 | 11 | 8 | 5 | 5 | 5 | 11 | 6 | 4 | 5 | 5 |
| hsa-miR-624-3p | MIMAT0004807 | miRNA | 92 | 4 | 6 | 7 | 3 | 2 | 3 | 11 | 5 | 1 | 4 | 4 | 6 |
| hsa-miR-625-5p | MIMAT0003294 | miRNA | 100 | 9 | 25 | 10 | 11 | 5 | 14 | 7 | 7 | 15 | 10 | 9 | 9 |
| hsa-miR-626 | MIMAT0003295 | miRNA | 100 | 17 | 26 | 31 | 24 | 23 | 24 | 24 | 26 | 21 | 32 | 20 | 15 |
| hsa-miR-627-3p | MIMAT0026623 | miRNA | 100 | 10 | 27 | 25 | 10 | 19 | 11 | 26 | 15 | 18 | 24 | 14 | 21 |
| hsa-miR-627-5p | MIMAT0003296 | miRNA | 100 | 13 | 24 | 27 | 21 | 20 | 11 | 16 | 20 | 19 | 15 | 14 | 14 |
| hsa-miR-628-3p | MIMAT0003297 | miRNA | 100 | 2 | 6 | 5 | 9 | 5 | 14 | 11 | 6 | 7 | 3 | 5 | 12 |
| hsa-miR-628-5p | MIMAT0004809 | miRNA | 92 | 7 | 14 | 14 | 1 | 6 | 6 | 5 | 8 | 8 | 7 | 3 | 10 |
| hsa-miR-629-5p | MIMAT0004810 | miRNA | 100 | 9 | 8 | 6 | 5 | 11 | 11 | 8 | 7 | 6 | 8 | 8 | 9 |
| hsa-miR-630 | MIMAT0003299 | miRNA | 100 | 26 | 21 | 13 | 14 | 16 | 13 | 14 | 7 | 8 | 6 | 5 | 12 |
| hsa-miR-631 | MIMAT0003300 | miRNA | 100 | 7 | 9 | 7 | 4 | 7 | 13 | 7 | 12 | 12 | 4 | 4 | 15 |
| hsa-miR-637 | MIMAT0003307 | miRNA | 83 | 4 | 2 | 6 | 4 | 6 | 4 | 2 | 1 | 1 | 4 | 7 | 4 |
| hsa-miR-638 | MIMAT0003308 | miRNA | 100 | 7 | 9 | 10 | 5 | 10 | 7 | 9 | 8 | 7 | 6 | 3 | 3 |
| hsa-miR-639 | MIMAT0003309 | miRNA | 100 | 15 | 23 | 24 | 14 | 19 | 13 | 23 | 21 | 13 | 24 | 20 | 10 |
| hsa-miR-640 | MIMAT0003310 | miRNA | 100 | 6 | 5 | 13 | 13 | 13 | 9 | 6 | 3 | 10 | 13 | 6 | 13 |
| hsa-miR-641 | MIMAT0003311 | miRNA | 83 | 7 | 5 | 3 | 5 | 2 | 8 | 5 | 3 | 1 | 1 | 4 | 6 |
| hsa-miR-642a-3p | MIMAT0020924 | miRNA | 83 | 1 | 5 | 4 | 4 | 5 | 7 | 8 | 5 | 8 | 1 | 3 | 4 |
| hsa-miR-642a-5p | MIMAT0003312 | miRNA | 100 | 15 | 39 | 22 | 31 | 22 | 18 | 20 | 14 | 15 | 20 | 14 | 19 |
| hsa-miR-643 | MIMAT0003313 | miRNA | 100 | 5 | 9 | 15 | 10 | 13 | 14 | 5 | 9 | 5 | 11 | 7 | 14 |
| hsa-miR-644a | MIMAT0003314 | miRNA | 100 | 6 | 10 | 11 | 8 | 6 | 12 | 8 | 9 | 7 | 7 | 9 | 17 |
| hsa-miR-648 | MIMAT0003318 | miRNA | 100 | 8 | 10 | 19 | 9 | 12 | 8 | 10 | 13 | 16 | 12 | 16 | 15 |
| hsa-miR-649 | MIMAT0003319 | miRNA | 100 | 5 | 8 | 15 | 9 | 7 | 8 | 10 | 7 | 8 | 5 | 11 | 8 |
| hsa-miR-650 | MIMAT0003320 | miRNA | 100 | 9 | 8 | 20 | 13 | 18 | 17 | 11 | 17 | 15 | 14 | 17 | 12 |
| hsa-miR-6503-3p | MIMAT0025463 | miRNA | 92 | 2 | 4 | 7 | 3 | 4 | 1 | 3 | 3 | 5 | 10 | 5 | 5 |
| hsa-miR-6503-5p | MIMAT0025462 | miRNA | 100 | 20 | 28 | 32 | 19 | 26 | 23 | 27 | 28 | 19 | 21 | 18 | 31 |

| | | | | | | | | | | | | | | |
|---|---|---|---|---|---|---|---|---|---|---|---|---|---|---|
| hsa-miR-651-3p | MIMAT0026624 | miRNA | 100 | 6 | 5 | 8 | 6 | 3 | 7 | 3 | 9 | 11 | 3 | 5 | 9 |
| hsa-miR-651-5p | MIMAT0003321 | miRNA | 100 | 11 | 20 | 22 | 10 | 17 | 11 | 8 | 11 | 20 | 14 | 15 | 11 |
| hsa-miR-6511a-3p | MIMAT0025479 | miRNA | 100 | 4 | 7 | 7 | 5 | 11 | 9 | 10 | 4 | 6 | 6 | 5 | 5 |
| hsa-miR-6511a-5p | MIMAT0025478 | miRNA | 100 | 2 | 4 | 5 | 6 | 7 | 9 | 8 | 4 | 9 | 5 | 5 | 5 |
| hsa-miR-652-3p | MIMAT0003322 | miRNA | 100 | 3 | 8 | 5 | 5 | 3 | 7 | 12 | 3 | 6 | 4 | 7 | 9 |
| hsa-miR-652-5p | MIMAT0022709 | miRNA | 100 | 4 | 7 | 9 | 5 | 11 | 7 | 6 | 3 | 2 | 6 | 2 | 10 |
| hsa-miR-654-3p | MIMAT0004814 | miRNA | 92 | 8 | 8 | 4 | 2 | 2 | 1 | 2 | 6 | 3 | 3 | 7 | 3 |
| hsa-miR-654-5p | MIMAT0003330 | miRNA | 100 | 6 | 3 | 4 | 5 | 7 | 8 | 5 | 6 | 5 | 7 | 10 | 8 |
| hsa-miR-655-3p | MIMAT0003331 | miRNA | 100 | 6 | 15 | 10 | 9 | 6 | 8 | 6 | 9 | 8 | 6 | 13 | 6 |
| hsa-miR-656-3p | MIMAT0003332 | miRNA | 100 | 29 | 41 | 15 | 17 | 16 | 10 | 14 | 15 | 15 | 22 | 14 | 21 |
| hsa-miR-660-3p | MIMAT0022711 | miRNA | 100 | 6 | 8 | 10 | 10 | 9 | 5 | 11 | 9 | 8 | 18 | 8 | 13 |
| hsa-miR-660-5p | MIMAT0003338 | miRNA | 100 | 2 | 35 | 15 | 5 | 13 | 21 | 11 | 10 | 18 | 16 | 9 | 21 |
| hsa-miR-661 | MIMAT0003324 | miRNA | 100 | 3 | 9 | 12 | 15 | 7 | 10 | 8 | 8 | 6 | 12 | 11 | 7 |
| hsa-miR-663a | MIMAT0003326 | miRNA | 100 | 6 | 10 | 8 | 8 | 8 | 8 | 8 | 4 | 6 | 3 | 8 | 8 |
| hsa-miR-664a-3p | MIMAT0005949 | miRNA | 100 | 17 | 148 | 40 | 78 | 31 | 71 | 33 | 39 | 53 | 45 | 31 | 53 |
| hsa-miR-664b-3p | MIMAT0022272 | miRNA | 100 | 11 | 14 | 9 | 17 | 17 | 14 | 10 | 19 | 13 | 9 | 10 | 9 |
| hsa-miR-664b-5p | MIMAT0022271 | miRNA | 100 | 4 | 5 | 8 | 4 | 10 | 4 | 3 | 4 | 3 | 3 | 6 | 7 |
| hsa-miR-665 | MIMAT0004952 | miRNA | 100 | 7 | 11 | 15 | 9 | 9 | 7 | 7 | 8 | 10 | 10 | 11 | 14 |
| hsa-miR-671-3p | MIMAT0004819 | miRNA | 100 | 4 | 11 | 8 | 5 | 3 | 9 | 3 | 9 | 5 | 6 | 4 | 6 |
| hsa-miR-671-5p | MIMAT0003880 | miRNA | 100 | 3 | 10 | 7 | 8 | 7 | 12 | 4 | 7 | 4 | 7 | 6 | 7 |
| hsa-miR-6720-3p | MIMAT0025851 | miRNA | 100 | 4 | 12 | 9 | 13 | 10 | 10 | 8 | 8 | 11 | 15 | 14 | 12 |
| hsa-miR-6721-5p | MIMAT0025852 | miRNA | 100 | 7 | 10 | 14 | 14 | 6 | 10 | 8 | 6 | 13 | 12 | 9 | 12 |
| hsa-miR-6724-5p | MIMAT0025856 | miRNA | 100 | 13 | 12 | 13 | 9 | 6 | 14 | 10 | 9 | 8 | 21 | 10 | 13 |
| hsa-miR-675-5p | MIMAT0004284 | miRNA | 100 | 6 | 9 | 10 | 7 | 9 | 10 | 8 | 8 | 8 | 5 | 4 | 8 |
| hsa-miR-7-5p | MIMAT0000252 | miRNA | 100 | 62 | 64 | 23 | 15 | 20 | 27 | 16 | 10 | 17 | 17 | 17 | 24 |
| hsa-miR-708-5p | MIMAT0004926 | miRNA | 100 | 2 | 10 | 2 | 5 | 2 | 10 | 2 | 5 | 10 | 2 | 3 | 6 |
| hsa-miR-744-5p | MIMAT0004945 | miRNA | 100 | 4 | 12 | 5 | 10 | 7 | 7 | 5 | 9 | 10 | 4 | 6 | 7 |
| hsa-miR-758-3p & miR-411-3p | MIMAT0003879 | miRNA | 100 | 43 | 19 | 17 | 16 | 9 | 21 | 11 | 12 | 6 | 7 | 12 | 17 |
| hsa-miR-758-5p | MIMAT0022929 | miRNA | 100 | 7 | 6 | 3 | 2 | 8 | 6 | 3 | 3 | 9 | 8 | 8 | 6 |
| hsa-miR-760 | MIMAT0004957 | miRNA | 100 | 4 | 10 | 7 | 5 | 9 | 11 | 7 | 6 | 8 | 10 | 9 | 8 |
| hsa-miR-761 | MIMAT0010364 | miRNA | 100 | 6 | 9 | 7 | 10 | 8 | 10 | 8 | 10 | 13 | 13 | 10 | 13 |
| hsa-miR-764 | MIMAT0010367 | miRNA | 100 | 3 | 13 | 28 | 12 | 19 | 19 | 18 | 13 | 16 | 16 | 18 | 14 |
| hsa-miR-765 | MIMAT0003945 | miRNA | 100 | 15 | 15 | 23 | 15 | 16 | 12 | 16 | 21 | 24 | 13 | 21 | 21 |
| hsa-miR-766-3p | MIMAT0003888 | miRNA | 100 | 4 | 3 | 6 | 6 | 4 | 4 | 3 | 7 | 4 | 10 | 4 | 6 |
| hsa-miR-766-5p | MIMAT0022714 | miRNA | 92 | 2 | 6 | 6 | 3 | 6 | 5 | 2 | 5 | 4 | 2 | 1 | 6 |
| hsa-miR-767-3p | MIMAT0003883 | miRNA | 92 | 3 | 9 | 7 | 4 | 8 | 6 | 1 | 5 | 7 | 7 | 3 | 6 |
| hsa-miR-767-5p | MIMAT0003882 | miRNA | 100 | 12 | 21 | 28 | 24 | 26 | 12 | 23 | 24 | 24 | 21 | 21 | 25 |
| hsa-miR-769-3p | MIMAT0003887 | miRNA | 100 | 11 | 16 | 19 | 10 | 11 | 12 | 15 | 17 | 16 | 11 | 8 | 19 |
| hsa-miR-769-5p | MIMAT0003886 | miRNA | 100 | 10 | 30 | 11 | 23 | 13 | 26 | 8 | 12 | 24 | 17 | 13 | 15 |
| hsa-miR-770-5p | MIMAT0003948 | miRNA | 100 | 10 | 7 | 10 | 2 | 9 | 8 | 7 | 7 | 10 | 6 | 6 | 8 |
| hsa-miR-802 | MIMAT0004185 | miRNA | 100 | 9 | 17 | 17 | 26 | 24 | 11 | 22 | 25 | 22 | 32 | 15 | 28 |
| hsa-miR-873-3p | MIMAT0022717 | miRNA | 100 | 24 | 25 | 33 | 26 | 24 | 21 | 26 | 24 | 30 | 37 | 29 | 28 |
| hsa-miR-873-5p | MIMAT0004953 | miRNA | 83 | 2 | 5 | 5 | 2 | 4 | 6 | 4 | 4 | 1 | 6 | 1 | 2 |
| hsa-miR-874-3p | MIMAT0004911 | miRNA | 100 | 7 | 14 | 11 | 9 | 8 | 14 | 9 | 10 | 12 | 11 | 6 | 11 |
| hsa-miR-874-5p | MIMAT0026718 | miRNA | 100 | 3 | 21 | 17 | 7 | 9 | 9 | 6 | 6 | 12 | 7 | 8 | 14 |

| | | | | | | | | | | | | | | | |
|---|---|---|---|---|---|---|---|---|---|---|---|---|---|---|---|
| hsa-miR-875-3p | MIMAT0004923 | miRNA | 100 | 2 | 13 | 2 | 6 | 8 | 7 | 2 | 4 | 3 | 3 | 4 | 2 |
| hsa-miR-876-3p | MIMAT0004925 | miRNA | 100 | 2 | 7 | 10 | 8 | 5 | 7 | 4 | 4 | 5 | 10 | 5 | 6 |
| hsa-miR-876-5p | MIMAT0004924 | miRNA | 100 | 8 | 11 | 12 | 7 | 5 | 6 | 14 | 13 | 10 | 18 | 10 | 20 |
| hsa-miR-877-5p | MIMAT0004949 | miRNA | 100 | 3 | 6 | 3 | 6 | 8 | 8 | 7 | 5 | 4 | 7 | 5 | 7 |
| hsa-miR-885-3p | MIMAT0004948 | miRNA | 83 | 1 | 10 | 3 | 1 | 6 | 7 | 8 | 6 | 5 | 13 | 2 | 2 |
| hsa-miR-885-5p | MIMAT0004947 | miRNA | 100 | 2 | 16 | 23 | 4 | 11 | 9 | 12 | 8 | 15 | 9 | 5 | 11 |
| hsa-miR-887-3p | MIMAT0004951 | miRNA | 83 | 4 | 7 | 8 | 3 | 6 | 7 | 4 | 4 | 1 | 4 | 1 | 4 |
| hsa-miR-887-5p | MIMAT0026720 | miRNA | 100 | 14 | 21 | 23 | 20 | 19 | 12 | 31 | 22 | 10 | 21 | 24 | 23 |
| hsa-miR-888-5p | MIMAT0004916 | miRNA | 100 | 16 | 20 | 21 | 14 | 26 | 14 | 12 | 19 | 20 | 14 | 13 | 16 |
| hsa-miR-889-3p | MIMAT0004921 | miRNA | 100 | 22 | 12 | 17 | 6 | 10 | 13 | 13 | 6 | 6 | 8 | 5 | 12 |
| hsa-miR-890 | MIMAT0004912 | miRNA | 100 | 6 | 8 | 11 | 3 | 8 | 2 | 5 | 4 | 3 | 2 | 9 | 7 |
| hsa-miR-891a-5p | MIMAT0004902 | miRNA | 100 | 13 | 24 | 28 | 16 | 20 | 17 | 26 | 29 | 8 | 20 | 23 | 27 |
| hsa-miR-891b | MIMAT0004913 | miRNA | 100 | 8 | 8 | 11 | 11 | 13 | 8 | 8 | 7 | 7 | 12 | 5 | 9 |
| hsa-miR-892a | MIMAT0004907 | miRNA | 100 | 5 | 3 | 8 | 7 | 6 | 7 | 9 | 6 | 4 | 4 | 10 | 5 |
| hsa-miR-892b | MIMAT0004918 | miRNA | 100 | 2 | 8 | 7 | 3 | 6 | 8 | 6 | 8 | 3 | 7 | 6 | 6 |
| hsa-miR-9-5p | MIMAT0000441 | miRNA | 100 | 3 | 103 | 4 | 25 | 6 | 3 | 10 | 5 | 7 | 4 | 4 | 4 |
| hsa-miR-922 | MIMAT0004972 | miRNA | 100 | 9 | 14 | 18 | 14 | 17 | 9 | 15 | 16 | 14 | 28 | 18 | 13 |
| hsa-miR-924 | MIMAT0004974 | miRNA | 75 | 2 | 3 | 6 | 1 | 2 | 1 | 2 | 4 | 5 | 4 | 2 | 1 |
| hsa-miR-92a-1-5p | MIMAT0004507 | miRNA | 92 | 13 | 8 | 9 | 17 | 7 | 6 | 3 | 4 | 8 | 3 | 13 | 1 |
| hsa-miR-92a-3p | MIMAT0000092 | miRNA | 100 | 70 | 30 | 39 | 26 | 24 | 32 | 11 | 11 | 51 | 22 | 35 | 89 |
| hsa-miR-92b-3p | MIMAT0003218 | miRNA | 100 | 19 | 12 | 11 | 9 | 6 | 12 | 9 | 8 | 6 | 5 | 7 | 13 |
| hsa-miR-93-5p | MIMAT0000093 | miRNA | 100 | 434 | 587 | 276 | 128 | 186 | 902 | 138 | 39 | 558 | 193 | 182 | 528 |
| hsa-miR-933 | MIMAT0004976 | miRNA | 100 | 9 | 16 | 22 | 10 | 14 | 17 | 18 | 15 | 10 | 30 | 16 | 21 |
| hsa-miR-934 | MIMAT0004977 | miRNA | 100 | 6 | 5 | 12 | 8 | 5 | 7 | 10 | 4 | 4 | 7 | 4 | 5 |
| hsa-miR-935 | MIMAT0004978 | miRNA | 100 | 3 | 8 | 7 | 4 | 4 | 8 | 6 | 5 | 5 | 4 | 4 | 2 |
| hsa-miR-936 | MIMAT0004979 | miRNA | 100 | 4 | 8 | 15 | 7 | 6 | 13 | 10 | 11 | 4 | 13 | 10 | 12 |
| hsa-miR-937-3p | MIMAT0004980 | miRNA | 100 | 5 | 12 | 16 | 12 | 20 | 16 | 14 | 11 | 5 | 8 | 15 | 12 |
| hsa-miR-939-5p | MIMAT0004982 | miRNA | 100 | 9 | 21 | 15 | 9 | 19 | 12 | 20 | 11 | 8 | 14 | 11 | 18 |
| hsa-miR-940 | MIMAT0004983 | miRNA | 100 | 6 | 5 | 9 | 4 | 2 | 3 | 4 | 3 | 5 | 3 | 3 | 5 |
| hsa-miR-941 | MIMAT0004984 | miRNA | 100 | 4 | 11 | 9 | 6 | 7 | 16 | 10 | 11 | 9 | 10 | 3 | 7 |
| hsa-miR-942-3p | MIMAT0026734 | miRNA | 75 | 1 | 7 | 2 | 3 | 1 | 4 | 1 | 2 | 3 | 7 | 2 | 3 |
| hsa-miR-942-5p | MIMAT0004985 | miRNA | 100 | 11 | 19 | 27 | 17 | 23 | 11 | 16 | 16 | 13 | 15 | 18 | 21 |
| hsa-miR-944 | MIMAT0004987 | miRNA | 92 | 4 | 7 | 8 | 10 | 5 | 8 | 1 | 8 | 12 | 6 | 9 | 9 |
| hsa-miR-95-3p | MIMAT0000094 | miRNA | 100 | 13 | 49 | 37 | 37 | 36 | 45 | 24 | 46 | 41 | 44 | 41 | 68 |
| hsa-miR-96-5p | MIMAT0000095 | miRNA | 100 | 3 | 24 | 13 | 6 | 15 | 13 | 16 | 7 | 13 | 21 | 10 | 27 |
| hsa-miR-98-3p | MIMAT0022842 | miRNA | 100 | 4 | 8 | 3 | 4 | 5 | 4 | 5 | 5 | 5 | 6 | 4 | 2 |
| hsa-miR-98-5p | MIMAT0000096 | miRNA | 100 | 291 | 433 | 150 | 179 | 147 | 296 | 88 | 67 | 275 | 144 | 171 | 431 |
| hsa-miR-99a-5p | MIMAT0000097 | miRNA | 100 | 289 | 3000 | 1022 | 1078 | 927 | 2387 | 675 | 419 | 2588 | 757 | 1384 | 2709 |
| hsa-miR-99b-5p | MIMAT0000689 | miRNA | 100 | 85 | 72 | 30 | 38 | 27 | 47 | 26 | 13 | 42 | 19 | 30 | 53 |
| ACTB | NM_001101.2 | miRNA | 100 | 6726 | 725 | 4951 | 270 | 4118 | 3808 | 788 | 175 | 3112 | 2299 | 1464 | 2185 |
| B2M | NM_004048.2 | miRNA | 100 | 1852 | 2971 | 4545 | 1201 | 4452 | 15096 | 781 | 279 | 6152 | 2150 | 1351 | 3101 |
| GAPDH | NM_002046.3 | miRNA | 100 | 7341 | 1268 | 3831 | 498 | 3571 | 4792 | 515 | 221 | 2466 | 1491 | 1423 | 2162 |
| RPL19 | NM_000981.3 | miRNA | 100 | 3644 | 2797 | 3651 | 825 | 2632 | 4899 | 674 | 173 | 3561 | 1216 | 970 | 1283 |
| RPLP0 | NM_001002.3 | miRNA | 100 | 5049 | 2085 | 6393 | 756 | 5295 | 6868 | 1234 | 281 | 4576 | 2311 | 2225 | 3184 |
| LIG_NEG_A | nmiR00810.1 | miRNA | 100 | 2 | 6 | 7 | 7 | 3 | 7 | 3 | 4 | 3 | 3 | 3 | 2 |

| | | | | | | | | | | | | | | | |
|---|---|---|---|---|---|---|---|---|---|---|---|---|---|---|---|
| LIG_NEG_B | nmiR00828.1 | miRNA | 100 | 10 | 8 | 9 | 9 | 8 | 7 | 6 | 9 | 7 | 5 | 4 | 5 |
| LIG_NEG_C | nmiR00803.1 | miRNA | 92 | 4 | 10 | 7 | 4 | 6 | 5 | 7 | 6 | 1 | 6 | 6 | 7 |
| LIG_POS_A | nmiR00813.1 | miRNA | 100 | 10792 | 6909 | 5845 | 5225 | 2980 | 9552 | 1296 | 3662 | 8410 | 1985 | 2772 | 6814 |
| LIG_POS_B | nmiR00809.1 | miRNA | 100 | 1462 | 849 | 737 | 723 | 340 | 1139 | 164 | 501 | 960 | 270 | 419 | 1012 |
| LIG_POS_C | nmiR00811.1 | miRNA | 100 | 299 | 245 | 140 | 125 | 94 | 333 | 39 | 112 | 274 | 58 | 118 | 201 |
| NEG_A | ERCC_00096.1 | SYSTEM | 100 | 4 | 10 | 11 | 7 | 9 | 12 | 12 | 14 | 13 | 7 | 12 | 7 |
| NEG_B | ERCC_00041.1 | SYSTEM | 100 | 3 | 9 | 5 | 8 | 11 | 9 | 9 | 4 | 8 | 3 | 4 | 10 |
| NEG_C | ERCC_00019.1 | SYSTEM | 100 | 3 | 9 | 5 | 7 | 7 | 8 | 10 | 6 | 5 | 5 | 5 | 3 |
| NEG_D | ERCC_00076.1 | SYSTEM | 100 | 10 | 7 | 18 | 7 | 9 | 7 | 3 | 5 | 6 | 3 | 2 | 9 |
| NEG_E | ERCC_00098.1 | SYSTEM | 100 | 11 | 18 | 14 | 16 | 16 | 10 | 8 | 11 | 12 | 12 | 11 | 17 |
| NEG_F | ERCC_00126.1 | SYSTEM | 100 | 9 | 22 | 15 | 15 | 13 | 8 | 9 | 14 | 8 | 12 | 13 | 16 |
| NEG_G | ERCC_00144.1 | SYSTEM | 100 | 2 | 4 | 12 | 6 | 11 | 7 | 4 | 6 | 7 | 5 | 3 | 5 |
| NEG_H | ERCC_00154.1 | SYSTEM | 100 | 6 | 11 | 15 | 10 | 18 | 10 | 14 | 18 | 6 | 12 | 15 | 16 |
| POS_A | ERCC_00117.1 | SYSTEM | 100 | 11548 | 10062 | 14634 | 11499 | 12580 | 10731 | 11396 | 12775 | 16998 | 11785 | 9713 | 11082 |
| POS_B | ERCC_00112.1 | SYSTEM | 100 | 5243 | 5110 | 7089 | 5252 | 6115 | 5286 | 5115 | 6209 | 6938 | 5546 | 4348 | 4977 |
| POS_C | ERCC_00002.1 | SYSTEM | 100 | 1407 | 1348 | 1884 | 1518 | 1586 | 1392 | 1473 | 1722 | 1647 | 1494 | 1152 | 1383 |
| POS_D | ERCC_00092.1 | SYSTEM | 100 | 383 | 301 | 446 | 329 | 391 | 331 | 323 | 353 | 442 | 348 | 300 | 327 |
| POS_E | ERCC_00035.1 | SYSTEM | 100 | 43 | 33 | 64 | 45 | 41 | 38 | 41 | 49 | 56 | 39 | 44 | 42 |
| POS_F | ERCC_00034.1 | SYSTEM | 100 | 31 | 24 | 54 | 32 | 38 | 31 | 34 | 36 | 41 | 28 | 31 | 37 |
| ath-miR159a | MIMAT0000177 | miRNA | 100 | 4 | 3 | 15 | 11 | 9 | 11 | 6 | 5 | 7 | 8 | 9 | 5 |
| cel-miR-248 | MIMAT0000304 | miRNA | 100 | 4 | 9 | 14 | 8 | 6 | 5 | 16 | 5 | 5 | 8 | 7 | 12 |
| cel-miR-254 | MIMAT0000310 | miRNA | 100 | 10 | 29 | 24 | 20 | 19 | 26 | 25 | 25 | 12 | 31 | 30 | 30 |
| osa-miR414 | MIMAT0001330 | miRNA | 100 | 17 | 31 | 24 | 23 | 30 | 19 | 19 | 19 | 23 | 22 | 20 | 21 |
| osa-miR442 | MIMAT0001605 | miRNA | 100 | 14 | 40 | 14 | 11 | 19 | 16 | 7 | 15 | 14 | 12 | 15 | 14 |
