# Supplementary material for: Toward Understanding the Role of miRNAs in Cleft Palate Only: Observations from Patient Tissues and In Vitro Assays
Source: Int J Mol Sci. 2026 Feb 24;27(5):2088. doi: 10.3390/ijms27052088 (PMC12985306; doi:10.3390/ijms27052088)
Supplement: Supplementary file 1 [file ijms-27-02088-s001.zip › Table S2.pdf]

Table S2. Primer sequences for qPCR analysis

miRNA primers: For NanoString® validation in primary CPO patient cell cultures (RNU44 reference; five deregulated miRNAs).

Target gene primers: For predicted miRNA targets (RPL13 reference).

Transfection control primers: For monitoring transfection efficiency.

Note: Only primers yielding successful amplification are shown; failed assays were excluded.

**miRNA**	**Forward**	**Reverse**
hsa-miR-205-5p	ccttcattccaccggagt	ggtccagtttttttttttttttcaga
hsa-miR-200c-3p	agtaatactgccgggtaatga	ggtccagtttttttttttttttcca
hsa-miR-125a-5p	ccctgagaccctttaacct	ggtccagtttttttttttttttcaca
hsa-miR-29b-3p	cagtagcaccatttgaaatcag	ggtccagtttttttttttttttaacac
hsa-miR-100-5p	cagaacccgtagatccga	gtccagtttttttttttttttcacaag
RNU44	cctggatgatgatagcaaatgc	gtccagtttttttttttttttagtcagt
**Target gene**	**Forward**	**Reverse**
AXIN2	CTTGACCCTGGGCCACTTTA	TTCATACATCGGGAGCACCG
CCNJ	CCAACGTGGCCTACAAGACT	TGCTTGCCCTCTCTGTTTGT
CDK19	GATTTCAAGGCGAAGCTGGC	TCTCTCTACAAGCCGACATGG
JADE1	GTGTGCAAAGTCCAGGAACAG	TCAGGGCCAACAGAAGGACT
PAX9	CTTGAACCGTGAGGCTCCTT	CAGGAAACTCCTTCCGCTGT
HIPK3	TGAGTACCAGCTCCTCAGGG	CCGGAAGAGTCAGATGTCGG
NR5A2	TGCACAGGAGTTAGTGGCAA	GCGGCATTGACTTGTTCCTG
RECK	TGATGTATGTGAACAGATTTTCTCC	ACCCAGCCATCAGACTTCTT
TBK1	TGAGTCTCGAGGAGGCCG	AAGTGCTCTGCATCTTGGCT
VASH2	ACCCGAGAGTCCTTGCCTAT	CTGATGGGGAACCGCTCAAT
ZEB1	GCAGTCCAAGAACCACCCTT	CGCATTTTCTTTTTGGGCGG
DVL3	ACATCTTCGGTGACCTCTGC	GGGTACTGGTACGGGAAAGC
MAP6	AGGAACCCCCAAAGGTGGAA	AGTGCAGGGAAATTTACTCTTTCG
PRSS35	GCTGAAGCGTGCTCACAAAA	GTCGGACACACTGCAAAACC
PRTG	CAGCCGCAGAGCAGAGAG	TAAAGCACCACACTCCTGGC
TNFSF4	TGGTGGTTGGGGTCACATTG	AGGATACCGATGTGATACCATGAG
RPL13	ATTCACAAGAAGGGAGACAG	GAAATTCTTCTCTTCCTCAGTG
**Reference transfection gene**	**Forward**	**Reverse**
HMGA2	GAAAAACGGCCAAGAGGCAG	GATCCAACTGCTGCTGAGGT
PTK9	GCAGTATAAGAGAGCGGATGCT	ACTCATCCCCATTGTCTATCTCG
